# A longitudinal DNA methylation atlas and its link to brain structure and mental health

**DOI:** 10.1038/s41380-026-03554-y

**Published:** 2026-03-31

**Authors:** Di Chen, Xinyang Yu, Wei Cheng, Linbo Wang, Shitong Xiang, Wei Zhang, Tobias Banaschewski, Arun L. W. Bokde, Herta Flor, Antoine Grigis, Hugh Garavan, Penny Gowland, Andreas Heinz, Rüdiger Brühl, Jean-Luc Martinot, Marie-Laure Paillère Martinot, Eric Artiges, Frauke Nees, Dimitri Papadopoulos Orfanos, Hervé Lemaître, Tomáš Paus, Luise Poustka, Sarah Hohmann, Nathalie Holz, Christian Bäuchl, Michael N. Smolka, Nilakshi Vaidya, Henrik Walter, Robert Whelan, Jianfeng Feng, Paul M. Thompson, Gunter Schumann, Tianye Jia, Sylvane Desrivières

**Affiliations:** 1 Shanghai Key Laboratory of Mental Health and Psychological Crisis Intervention, School of Psychology and Cognitive Science, East China Normal University, Shanghai, China; 2 Institute of Science and Technology for Brain-Inspired Intelligence, Fudan University, Shanghai, China; 3 Key Laboratory of Computational Neuroscience and Brain-Inspired Intelligence (Fudan University), Ministry of Education, Shanghai, China; 4 Social, Genetic and Developmental Psychiatry Centre, Institute of Psychiatry, Psychology & Neuroscience, King’s College London, London, UK; 5 Department of Child and Adolescent Psychiatry and Psychotherapy, Central Institute of Mental Health, Medical Faculty Mannheim, Heidelberg University, Mannheim, Germany; 6 Discipline of Psychiatry, School of Medicine and Trinity College Institute of Neuroscience, Trinity College Dublin, Dublin, Ireland; 7 Institute of Cognitive and Clinical Neuroscience, Central Institute of Mental Health, Medical Faculty Mannheim, Heidelberg University, Mannheim, Germany; 8 Department of Psychology, School of Social Sciences, University of Mannheim, Mannheim, Germany; 9 NeuroSpin, CEA, Université Paris-Saclay, F-91191 Gif-sur-Yvette, France; 10 Departments of Psychiatry and Psychology, University of Vermont, Burlington, VT USA; 11 Sir Peter Mansfield Imaging Centre School of Physics and Astronomy, University of Nottingham, Nottingham, UK; 12 Department of Psychiatry and Psychotherapy, University of Tübingen, and German Center for Mental Health (DZPG), Site Tübingen, Tübingen, Germany; 13 Department of Psychiatry and Psychotherapy, Technische Universität Dresden, Dresden, Germany; 14 Physikalisch-Technische Bundesanstalt (PTB), Braunschweig and Berlin, Germany; 15 Institut National de la Santé et de la Recherche Médicale, INSERM U1299 “Developmental trajectories & psychiatry”; Université Paris-Saclay, Université Paris Cité, Ecole Normale supérieure Paris-Saclay, CNRS; Centre Borelli, UMR9010 Gif-sur-Yvette, France; 16 Psychiatry Department, Lad-D-Psy, EPS Barthélémy Durand, Etampes, France; 17 AP-HP. Sorbonne Université, Department of Child and Adolescent Psychiatry, Pitié-Salpêtrière Hospital, Paris, France; 18 Institute of Medical Psychology, Ludwig-Maximilians-Universität (LMU) in Munich, Munich, Germany; 19 Institut des Maladies Neurodégénératives, UMR 5293, CNRS, CEA, Université de Bordeaux, 33076 Bordeaux, France; 20 Departments of Psychiatry and Neuroscience, Faculty of Medicine and Centre Hosptalier Universitaire Sainte-Justine, University of Montreal, Montreal, QC Canada; 21 Department of Child and Adolescent Psychiatry and Psychotherapy, Heidelberg University Hospital, Blumenstrasse 8, 69115 Heidelberg, Germany; 22 Department of Child and Adolescent Psychiatry, Psychotherapy and Psychosomatics, University Medical Center Hamburg-Eppendorf, Hamburg, Germany; 23 Centre for Population Neuroscience and Stratified Medicine (PONS), Department of Psychiatry and Psychotherapy, Charité Universitätsmedizin Berlin, Berlin, Germany; 24 Charité–Universitätsmedizin Berlin, corporate member of Freie Universität Berlin, Humboldt-Universität zu Berlin, and Berlin Institute of Health, Department of Psychiatry and Psychotherapy, Campus Charité Mitte, Charitéplatz 1, Berlin, Germany; 25 School of Psychology and Global Brain Health Institute, Trinity College Dublin, Dublin, Ireland; 26 Department of Computer Science, University of Warwick, Coventry, UK; 27 Imaging Genetics Center, Stevens Neuroimaging & Informatics Institute, Keck School of Medicine of University of Southern California, Marina del Rey, CA USA; 28 Centre for Population Neuroscience and Stratified Medicine (PONS), Department of Psychiatry and Neuroscience, Charité Universitätsmedizin Berlin, Berlin, Germany; 29 Centre for Population Neuroscience and Precision Medicine (PONS), Institute for Science and Technology of Brain-inspired Intelligence (ISTBI), Fudan University, Shanghai, China; 30 National Institute on Drug Dependence and Beijing Key Laboratory of Drug Dependence Research, Peking University, Beijing, China; 31 School of Psychology, Southampton University, Southampton, UK

**Keywords:** Genetics, Psychiatric disorders

## Abstract

Epigenetic mechanisms are thought to contribute to neurodevelopmental vulnerability for psychiatric disorders, yet longitudinal evidence linking DNA methylation (DNAm) to brain maturation and psychopathology is limited. Using epigenome-wide DNAm (372,582 CpGs) and whole-brain structural MRI data from the IMAGEN cohort (*n* = 506, ages 14–19), we identified 18 co-regulated DNAm clusters, ten enriched for brain-expressed genes involved in neuronal development and signalling. The clusters showed consistent longitudinal change and were reproducible in independent adult samples (PPMI, *n* = 513; ADNI, *n* = 606). Multivariate analyses revealed coordinated coupling between DNAm change and cortical-subcortical maturation, such that greater DNAm reductions in brain-related clusters associated with greater cortical thinning and subcortical volume changes in the fronto-limbic-striatal axis. Increases in depressive symptoms, and frequency of cannabis use and binge drinking were linked to DNAm changes, with mediation models supporting DNAm as a mechanistic bridge between behaviour and brain change. Two clusters (C1 and C7) were associated with depressive and negative psychotic symptoms across adolescence. Associations of these clusters with depressive symptoms replicated in the PPMI dataset. Their coupling with amygdala–striatal maturation suggests that these DNAm signatures index environmentally shaped affective–motivational neurodevelopment.

## Introduction

Epigenetic modifications, particularly DNA methylation (DNAm) at cytosine-phosphate-guanine (CpG) dinucleotides, regulate gene expression and cellular identity across development and in response to environmental exposures [1]. Through this mechanism, genetic predispositions interact with developmental and environmental influences to shape trajectories of health and disease [2]. Although DNAm variation has been examined extensively in relation to disease states, less is known about its dynamic organisation during major neurodevelopmental transitions, such as adolescence, and how such changes relate to brain maturation and emerging mental health problems.

Blood-based DNAm has proven to be a useful proxy for investigating brain-relevant epigenetic processes. Despite tissue-specific differences, convergent studies have shown that subsets of CpG sites display correlated methylation patterns between blood and cortical brain regions [3], and that blood-based DNAm has been associated with MRI-derived brain structure in regions such as the hippocampus and cortical association areas [4–6]. However, studies investigating associations between DNAm and brain measures are still too scarce and lack systematic coverage, as most have been cross-sectional or region-specific, providing only limited insight into coordinated DNAm-brain associations at the whole-brain level.

Environmental exposures, including prenatal stress, early adversity, or substance use, can leave lasting DNAm signatures [7, 8], and DNAm variation has been associated with multiple psychiatric disorders, including ADHD, depression and schizophrenia [6, 9, 10]. Importantly, DNAm exhibits systematic age-related changes, especially in adolescence –a period characterised by greater susceptibility to stress, extensive brain maturation [11, 12], and heightened emergence of affective and substance-use disorders [13, 14]. Thus, adolescence may represent a critical window during which epigenetic processes influence neurodevelopmental vulnerability. Yet, the scarcity of longitudinal DNAm datasets has limited the identification of developmentally co-regulated DNAm patterns and their relevance for brain maturation and mental health.

Adolescence is also a developmental window during which depressive symptoms, psychotic-like experiences, and substance use frequently co-occur. Depression is one of the most common internalising conditions emerging during this period, and altered DNAm has been consistently linked to major depressive disorder [15]. Similarly, DNAm differences have been observed in individuals at clinical high risk for psychosis and in schizophrenia [16, 17]. Notably, depressive symptoms and negative psychosis-like symptoms show substantial phenomenological and mechanistic overlap [18, 19], which are closely tied to neural circuits involved in affect regulation and reward processing [20]. In parallel, cannabis use is a risk factor for both depressive and psychotic outcomes [21] and contributes to long-term alterations in adolescent brain maturation [22]. Together, these converging evidence justify joint examination of depression, psychosis-like symptoms, and substance use within a coherent neurodevelopmental framework grounded in DNAm plasticity and brain maturation.

Here, we address this by constructing a longitudinal DNAm atlas using IMAGEN [23], a deeply phenotyped adolescent cohort. Applying weighted gene co-expression network analysis (WGCNA) [24] on DNAm data acquired at ages 14 and 19, we identified clusters of CpG sites that show coordinated developmental patterns. We validated the stability of these clusters in two independent samples of older adults: PPMI [25] and ADNI [26]. We then examined whether longitudinal DNAm change in adolescence relates to structural brain maturation (changes in cortical thickness, surface area, and subcortical volumes) from ages 14 to 19, and whether these DNAm-brain patterns are associated with changes in depressive symptoms, psychosis symptoms, and frequency of substance use (alcohol, binge drinking, cigarette smoking, cannabis) across adolescence. An overview of the study design is provided in Fig. S1.

## Results

### Identification of longitudinal DNA methylation patterns

Our main analyses included data from IMAGEN, a longitudinal cohort of typically developing adolescents (*n* = 506, 47% males). As expected for this developmental period, the prevalence of risky behaviours (*i.e*., substance use) increased from ages 14 to 19 (Table S1).

**Fig. 1:**
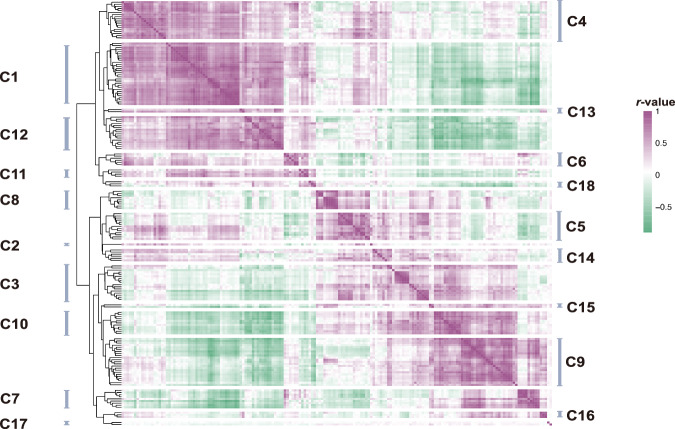
Longitudinal human DNA methylation (DNAm) atlas. Correlation matrix of longitudinal DNAm change (ΔDNAm = DNAm_19_ *-* DNAm_14_) across 175 consensus co-methylation modules identified using WGCNA. Each cell reflects the Pearson correlation between longitudinal change scores of two modules. Hierarchical clustering of this matrix yielded 18 higher-order DNAm clusters (C1–C18), representing coordinated methylation trajectories across adolescence.

To identify coordinated patterns of DNAm change across adolescence, we applied a two-step approach. First, WGCNA was applied to identify consensus modules –defined as consistently co-methylated at ages 14 and 19, which yielded 175 DNAm modules. For each module, we calculated longitudinal change in DNAm (ΔDNAm = age19 - age14) and then clustered modules based on similarity in these change patterns. This hierarchical clustering procedure identified 18 DNAm clusters (*i.e*., C1-C18) that showed consistent patterns of coordinated methylation change across adolescence, and spanning all 22 autosomal chromosomes (Fig. 1; Fig. S2; Table S2).

**Fig. 2:**
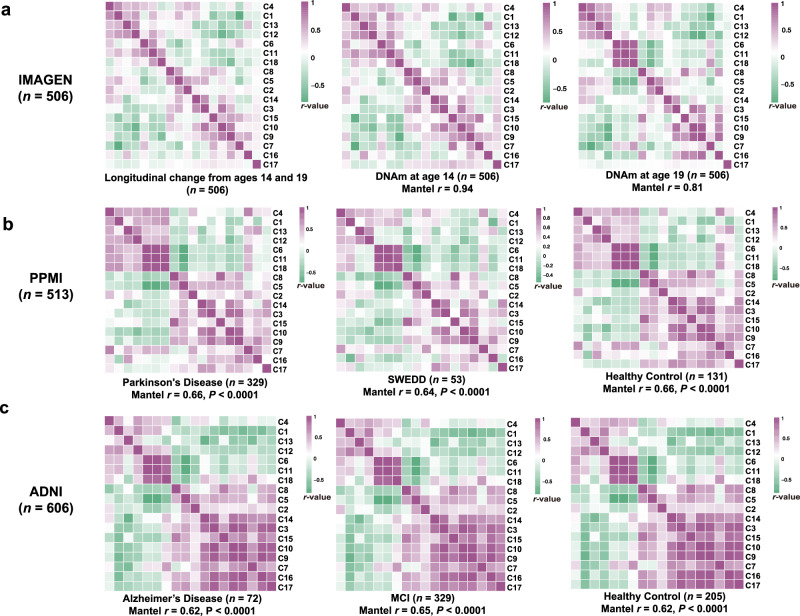
Robustness and lifespan generalizability of the DNAm clusters. Similarity matrices (Pearson *r*) summarising inter-cluster DNAm relationships across cohorts. **a** IMAGEN, similarity matrices derived from ΔDNAm, DNAm_14_, and DNAm_19_ demonstrate stable cluster structure across time. **b** Replication in PPMI and (**c**) ADNI: similarity matrices computed in each cohort show significant correspondence to the IMAGEN reference pattern (Mantel correlations; 10,000 permutations; Bonferroni-corrected one-tailed P-values). SWEDD “scans without evidence of dopaminergic deficit”, MCI mild cognitive impairment.

To evaluate whether these longitudinal DNAm patterns captured variance shared with DNAm measured at either age, we applied Mantel correlation [27] to compare similarity matrices (inter-cluster *r*-values) across timepoints. DNAm patterns at both age 14 and age 19 closely resembled the longitudinal covariation pattern (Mantel *r* = 0.94 at age 14; Mantel *r* = 0.81 at age 19; Fig. 2a). These results indicate that the identified clusters reflect stable, developmentally conserved DNAm organisation that is observable at each age as well as in their longitudinal change.

### Robustness of the DNAm pattern

Importantly, the identified DNAm clusters were technically robust, with consensus-based longitudinal clustering demonstrating significantly higher internal consistency across array platforms and batches than non-consensus approaches (see Methods and Fig. S3), supporting that the observed patterns reflect biological rather than methodological signal.

We further tested the robustness of the DNAm pattern in two independent datasets of older adults (PPMI and ADNI; Table S1). The cluster similarity structure identified in adolescence was preserved across diagnostic and healthy groups in both datasets: PPMI: Mantel *r* = 0.66 (Parkinson’s disease), *r* = 0.64 (Parkinson’s disease - SWEDD), *r* = 0.66 (healthy controls) (all *P*_perm_ < 0.0001; Fig. 2b); ADNI: Mantel *r* = 0.62 (Alzheimer’s disease), *r* = 0.65 (mild cognitive impairment), *r* = 0.62 (healthy controls) (all *P*_perm_ < 0.0001; Fig. 2c).

This cross-cohort indicates that the identified DNAm clusters represent a stable organisational architecture of the methylome that extends beyond adolescence into later life.

### DNAm levels within clusters and associations with sex, pubertal development, and socioeconomic status

Table S3 summarises DNAm levels within each cluster at ages 14 and 19, the magnitude of developmental change, and associations with sex, pubertal development (PD), and socioeconomic stress (SES). Most clusters showed small-to-moderate decreases in DNAm from age 14 to 19, with the largest reductions observed in C13, C6, C7, and C11. Several clusters exhibited significant sex differences (*P*_FDR_ < 0.05; |Cohen’s *d*| = 0.21–0.95). Notably, C1 and C2 showed higher methylation in males at both ages, with effects stronger at age 14. In contrast, C4 and C8 displayed a directional reversal across adolescence (C4: female>male at age 14, male>female at age 19; C8: male>female at age 14, female>male at age 19). Associations with SES were minimal: only C12 showed a significant association, with higher SES associated with lower DNAm (*r* = −0.15, *P*_FDR_ = 0.0135). No DNAm cluster showed significant associations with pubertal development (Table S3).

Overall, adolescent DNAm variation was shaped primarily by sex and longitudinal developmental processes, with little evidence for systematic associations with socioeconomic factors.

### Functional and tissue-specific characterisation of DNAm clusters

**Fig. 3:**
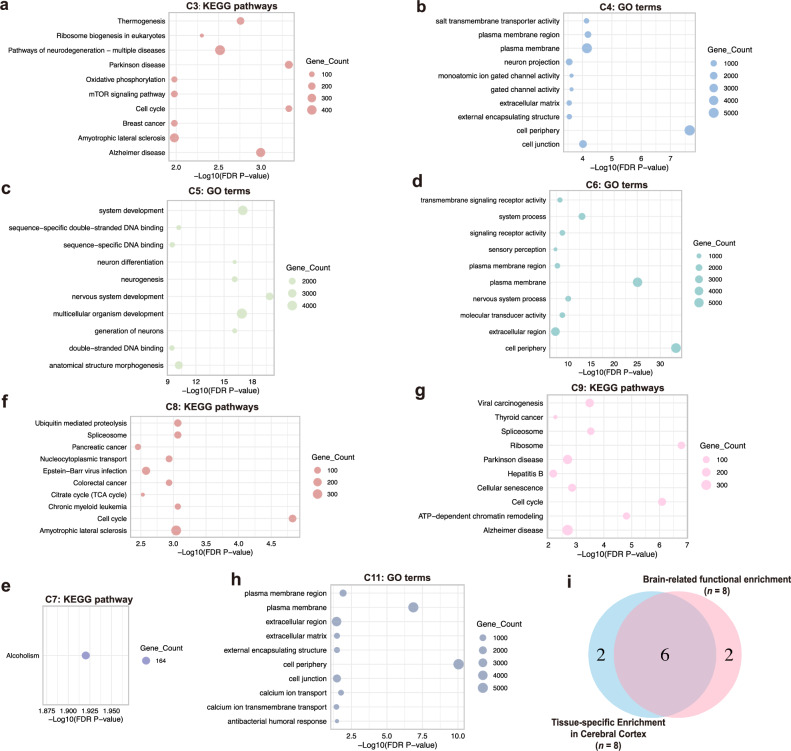
Functional enrichment analyses identify brain-related epigenetic clusters. **a**–**h** Illustrations of the top ten significantly enriched items for KEGG pathways or GO terms in the clusters related to brain function (*n* = 8) (*i.e*., Clusters-3,4,5,6,7,8,9,11). FDR-corrected P-values were used for statistical significance. **i** Venn diagram displaying the numbers of brain-related DNAm clusters identified in the tissue-specific (*n* = 8) and functional (*n* = 8) enrichment analyses. A total of 10 brain-related DNAm clusters were identified, including six identified by both enrichment analyses and 4 identified by either one of the enrichment analyses. KEGG Kyoto encyclopedia of genes and genomes; GO gene ontology.

To infer the biological systems represented within each DNAm cluster, we performed tissue-specific gene expression enrichment (Table S4), Gene Ontology (GO), and KEGG pathway analyses (Table S5, Fig. 3). These analyses revealed marked functional differentiation across clusters, pointing to coordinated epigenetic regulation of neural, immune, and chromatin-remodelling processes during adolescence.

A subset of ten clusters (C1, C3, C4, C5, C6, C7, C8, C9, C11, and C12) showed convergent evidence for central nervous system relevance (Fig. 3i). Most of these clusters (C1, C3, C4, C5, C6, C7, C11, and C12) were enriched for cerebral cortex-expressed genes, suggesting that they index methylation-based coordination of neuronal structural and functional programmes. Within this group, C4, C6 and C11 were enriched for plasma membrane organisation, synaptic signalling, ion channel activity, and neuron projection, pointing to regulation of cell-cell communication, connectivity, and excitability. C5 was distinguished by strong enrichment for neurodevelopmental processes, including nervous system development, neurogenesis, and neuron differentiation, indicating a core axis of developmentally patterned methylation that aligns with brain maturation in adolescence. Other clusters within the brain-related group pointed toward cellular maintenance and metabolic regulation. C1 showed enrichment for ATP- and nucleotide-binding functions, consistent with neuronal energy mobilisation and biosynthetic regulation, whereas C3 and C9 were enriched for KEGG pathways related to neurodegeneration (Parkinson’s disease, Alzheimer’s disease, amyotrophic lateral sclerosis) and cell-cycle and senescence pathways, suggesting that these clusters may reflect molecular processes involved in long-term neuronal maintenance, vulnerability, or resilience. C8, while not enriched for cortex-specific expression, also showed enrichment for pathways related to neurodegeneration (amyotrophic lateral sclerosis) and for cell-cycle control, RNA splicing, ubiquitin-mediated turnover, and nucleocytoplasmic transport, pointing to regulation of proteostasis and nuclear-cytoplasmic communication. In contrast, C2 showed strong enrichment for peripheral immune tissues (spleen, lymph node, bone marrow) and for GO/KEGG pathways involving leukocyte activation, cytokine signalling, NF-κB and HIF-1 pathways, indicating a systemic immune signalling axis that is distinct from the brain-enriched clusters. Finally, C10 and C15 were enriched for chromatin regulation, RNA processing, and nuclear protein complex assembly, reflecting broad transcriptional regulatory machinery rather than region- or system-specific biology.

Together, these findings suggest that adolescent DNAm organisation reflects three coordinated molecular domains: (*i*) a neuronal and synaptic patterning axis (C1, C3, C4, C5, C6, C7, C8, C9, C11, C12), (*ii*) a peripheral immune regulatory axis (C2), and (*iii*) a global chromatin–transcriptional regulation axis (C10, C15). Because our central aim was to test how adolescent brain maturation relates to epigenetic variation, we focused subsequent analyses on the ten brain-related DNAm clusters (*i.e*., C1, C3, C4, C5, C6, C7, C8, C9, C11, C12) in the neuroimaging and psychopathology models presented below. These clusters were characterised by their differing overall DNAm levels and consistent patterns of change across adolescence (Table S3).

### Developmental changes in cortical and subcortical morphology from ages 14 to 19

To relate the identified DNA methylation clusters to adolescent brain maturation, we first investigated changes in cortical and subcortical structure from ages 14 to 19 (*i.e*., Δbrain = brain_19_ - brain_14_). We observed widespread and regionally patterned changes. At the cortical level, the majority of brain regions showed significant reductions in cortical thickness (CT) over time (Table S6). The largest decreases were observed in bilateral fronto-parietal association cortices, including the rostral middle frontal, superior and inferior parietal, supramarginal, precuneus, and superior frontal regions (Cohen’s *d* = −0.7 to −1.0, *P*_FDR_ < 0.001). Additional cortical thinning was evident in the cingulate cortex and sensorimotor regions, though with comparatively smaller effect sizes (Cohen’s *d* = −0.3 to −0.6). In contrast, a small subset of temporal and paralimbic regions exhibited relative preservation or increases in cortical thickness across the same period. These included the bilateral entorhinal cortex and temporal pole, as well as the left inferior temporal cortex (Cohen’s *d* = 0.23 to 0.39, all *P*_FDR_ < 0.01). Fusiform and middle temporal regions showed no significant change. This pattern is consistent with a non-uniform maturational trajectory, with higher-order association cortices and comparatively later structural development in temporal regions [28, 29].

Subcortical grey matter volume (SubCortVol) changes were more regionally specific and of smaller magnitude. Significant reductions were observed in the bilateral caudate and putamen (all, *P*_FDR_ < 0.001), and thalamus (both sides, *P*_FDR_ < 0.05). A decrease was also observed in the left nucleus accumbens (*P*_FDR_ = 0.0039), with a trend in the same direction on the right. In contrast, bilateral amygdala volumes increased modestly, whereas hippocampal and pallidal volumes remained stable. Bilateral lateral ventricles increased in size (both *P*_FDR_ < 0.05), consistent with the observed cortical reductions [30, 31].

Taken together, these results indicate broad cortical thinning concentrated in fronto-parietal association networks, accompanied by targeted volumetric reductions in striatal and thalamic structures and selective increases in amygdala and anterior temporal cortices across adolescence. The overall pattern reflects continued structural refinement of networks supporting cognitive control, reward processing, and socio-emotional function during the transition from mid- to late adolescence [13].

### Associations between changes in DNAm and brain maturation

We next investigated relationships between changes in DNAm from ages 14 to 19. All variables were entered as change scores (Δ = age_19_ – age_14_), such that more negative Δvalues reflect greater age-related reduction (e.g., cortical thinning or relative demethylation), whereas more positive values reflect relative preservation or expansion. In this framework, positive canonical loadings indicate DNAm clusters or brain regions where individuals showing relative preservation load more strongly on the CCA component, and negative loadings indicate clusters or regions where individuals showing greater age-related reduction contribute more strongly.

**Fig. 4:**
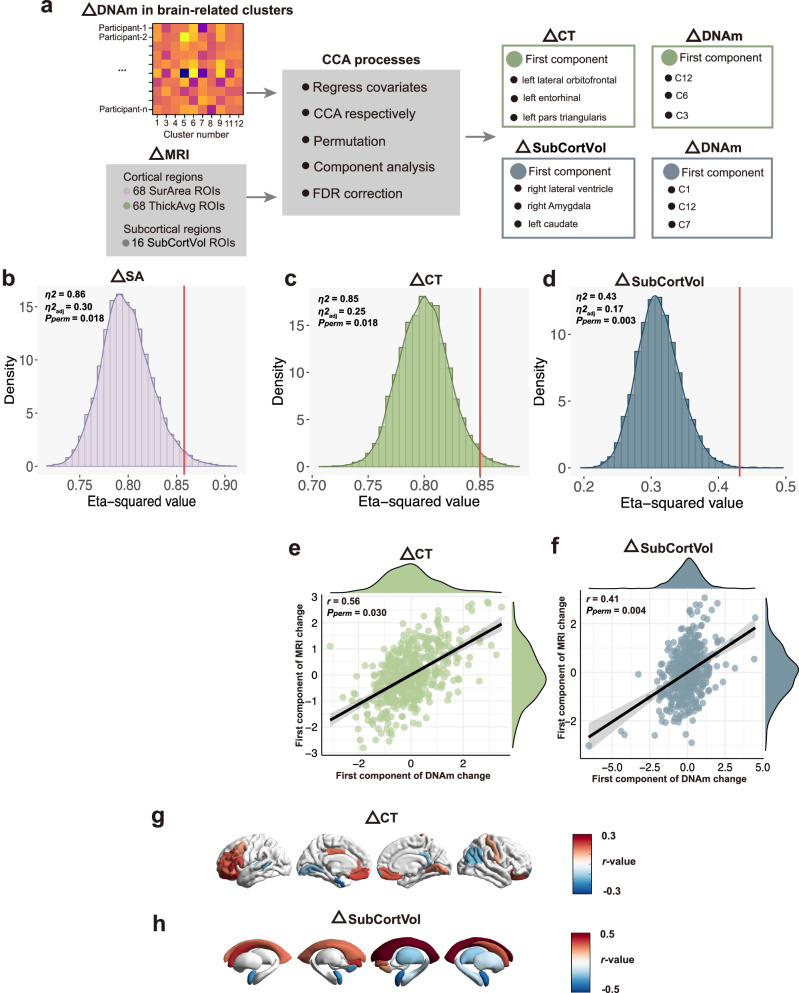
Multivariate associations between DNAm change and brain maturation. **a** Schematic overview of the canonical correlation analysis (CCA) linking ΔDNAm in the 10 brain-related clusters to longitudinal brain structural change (ΔMRI: Δcortical thickness (CT), Δcortical surface area (SA), Δsubcortical volume). **b**–**d** Permutation-derived η² distributions (10,000 permutations) for each CCA model. **e,** **f** Canonical correlations for the first significant component. **g,** **h** Brain maps displaying regional loadings (CCA weights) for Δcortical thickness and Δsubcortical volume components; FDR-corrected *P* < 0.05. Warmer colours indicate regions that load positively, cooler colours indicate regions that load negatively.

We ran three separate CCAs examining the association between changes in DNAm and changes in (i) cortical surface area (ΔSA), (ii) cortical thickness (ΔCT), and subcortical volumes (ΔSubCortVol) (Fig. 4a). Significant multivariate associations were found for ΔSA, ΔCT, and ΔSubCortVol (Fig. 4b–d). Statistical significances were observed for the first canonical components for the ΔCT (*r* = 0.56, *P*_perm_ = 0.030, Fig. 4e) and ΔSubCortVol (*r* = 0.41, *P*_perm_ = 0.004, Fig. 4f) analyses, but not for ΔSA (*P*_perm_ = 0.089). We therefore focused on the interpretation of the first canonical components of the ΔCT and ΔSubCortVol analyses (Tables S7–S8; Fig. 4g, h).

#### Cortical thickness

For ΔCT, seven DNAm clusters contributed significantly to the canonical component (|*r*| ≥ 0.12, *P*_FDR_ < 0.05). The most influential were C12 (*r* = −0.51) and C6 (*r* = 0.44), with additional contributions from C3, C7, C9 and C11 (*r* ≥ 0.12), and C5 (*r* = −0.18). C12 -which showed a moderate decrease in methylation (Table S3)– loaded negatively, indicating that greater decreases in methylation in this cluster were associated with greater cortical thinning. In contrast, C6, which also decreased substantially in methylation, loaded positively, indicating that individuals with more preserved methylation levels in this cluster showed less cortical thinning. On the brain side, positive loadings (*r* ≥ 0.11) were observed in medial and lateral prefrontal (e.g., lateral orbitofrontal cortex, medial orbitofrontal cortex, pars triangularis, pars opercularis, and rostral/caudal middle frontal cortex) and cingulate regions. In contrast, negative loadings (*r* ≤ −0.12) were observed in temporal and parietal-association regions, including the entorhinal cortex and banks of the superior temporal sulcus.

Thus, the cortical CCA identifies two coordinated maturational profiles: (1) a profile of greater DNA methylation decrease (*e.g*., C12 and C5) co-occurring with greater thinning in temporal-paralimbic regions, and (2) a relative preservation profile linking less DNAm decrease (in C6, C3, C7, C9 and C11) with more stable cortical thickness in medial and lateral prefrontal regions.

#### Subcortical Volumes

For ΔSubCortVol, seven DNAm clusters again contributed significantly (all |*r*| ≥ 0.11, *P*_FDR_ < 0.05). Key contributors included C1 (*r* = 0.44), Cluster 7 (*r* = −0.36), C11 (*r* = 0.23), and C12 (*r* = 0.40). Here, greater DNAm decrease in negatively loaded clusters (C7 and C6) were associated with greater volume reductions in the bilateral amygdala, left nucleus accumbens and right thalamus, hippocampus and putamen, while relative maintenance of methylation in C1, C11, C12 and C8 was associated with relative volume preservation in the bilateral caudate, and right nucleus accumbens, as well as reduced ventricular enlargement.

### Longitudinal associations between DNAm change, cortical maturation, and adolescent psychopathology

The fronto-limbic-striatal axis highlighted above –encompassing prefrontal regions supporting cognitive control and valuation (lateral and medial orbitofrontal cortices), temporal-paralimbic regions involved in socio-emotional processing (entorhinal cortex, superior temporal sulcus), and striatal structures involved in reward learning and motivation– is central to affect regulation and reward sensitivity. Regions in this network have been repeatedly implicated in risk for depression and substance-use escalation, particularly in adolescence [14, 32–34].

Descriptive data (Table S1) show significant increases in lifetime substance use from ages 14 to 19 (all *P* < 0.0001). Accordingly, we next examined whether changes in depressive symptoms and lifetime frequency of substance use across adolescence were associated with the coordinated patterns of DNAm and cortical and subcortical changes identified in the first CCA components (Table S9). Significant associations were observed with the DNAm and cortical changes captured by the first ΔCT CCA component: Increased frequency in cannabis use and binge drinking were associated with greater DNAm reduction (*r* = −0.16) and greater cortical thinning (*r* < −0.10). Increases in depressive symptoms were also associated with greater DNAm reduction (*r* = −0.12) and greater cortical thinning (*r* = −0.14) (all *P*_*FDR*_ < 0.05). In contrast, changes in alcohol use frequency and tobacco smoking showed non-significant associations. No significant associations were observed for subcortical changes identified by the ΔSubCortVol CCA. Taken together, these results suggest that adolescents who show greater increases in depressive symptoms, cannabis use, and binge drinking also exhibit greater reductions in DNAm and greater cortical thinning within a shared fronto-limbic maturation axis.

**Fig. 5:**
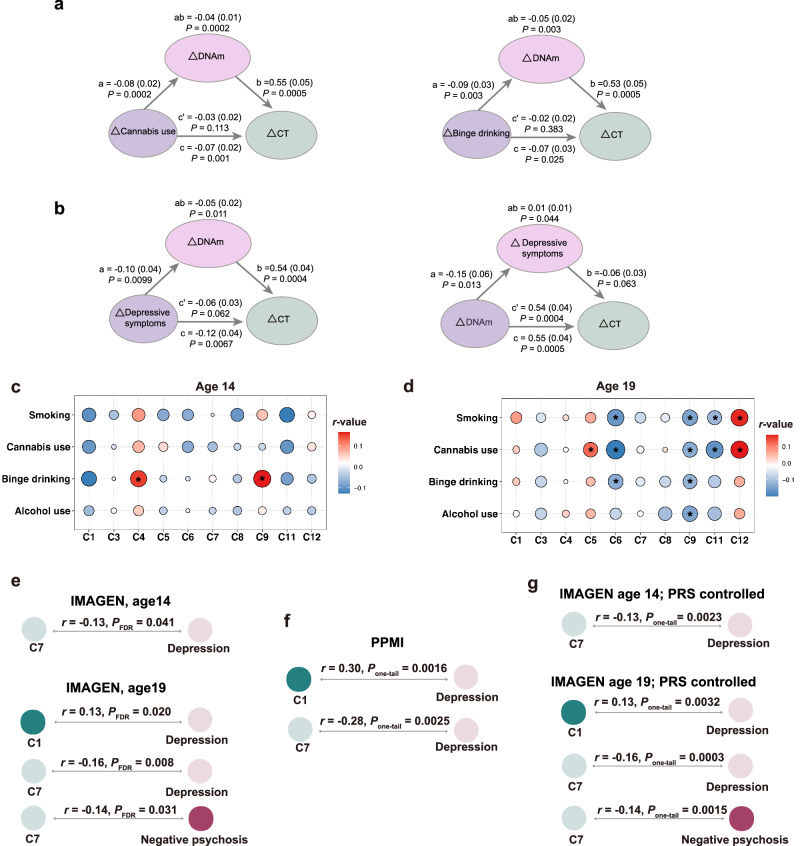
Mediation models and cluster-specific DNAm associations with psychopathology. **a** ΔSubstance use → ΔDNAm → ΔCortical Thickness: Indirect effects shown separately for cannabis use (left) and binge drinking (right), indicating full mediation. **b** Bidirectional models involving depressive symptoms: Left, ΔDepressive symptoms → ΔDNAm → ΔCortical Thickness; Right, ΔDNAm → ΔDepressive symptoms → ΔCortical Thickness. Path coefficients and P-values are shown. **c,** **d** Associations between DNAm levels in brain-related clusters and substance use at ages 14 and 19 (two-tailed *P*_FDR_ < 0.05). **e** Significant associations of clusters C1 and C7 with depressive symptoms and negative psychotic symptom dimensions across adolescence (Pearson *r*; FDR-corrected two-tailed P-values). **f** Replication of C1 and C7 associations with depressive symptoms in PPMI (Pearson *r*; one-tailed P-values). **g** Associations of C1 and C7 with depressive symptoms and negative psychotic symptoms in IMAGEN after adjusting for depression or schizophrenia PRS, respectively (Pearson *r*; one-tailed P-values).

We used longitudinal mediation models to test whether DNAm change mediates the link between behavioural changes and cortical maturation, examining directional pathways among changes in DNAm, depressive symptoms, cannabis use, binge drinking and cortical thickness. Given the known effects of substances of abuse (e.g., tobacco, cannabis and alcohol) both on DNA methylation [35] and adolescent brain maturation [22, 36, 37], we tested mediation models where Δsubstance use predicted ΔCT through ΔDNAm. Both Δcannabis use and Δbinge drinking showed a significant indirect association with ΔCT via ΔDNAm (Δcannabis use: indirect = −0.04, *P* = 0.0002; Δbinge drinking: indirect = −0.05, *P* = 0.003; Fig. 5a), such that increases in cannabis use/binge drinking were associated with greater DNAm reduction, which in turn related to greater cortical thinning. The direct effects were non-significant (Δcannabis use: c′ = −0.03, *P* = 0.113; Δbinge drinking: c′ = −0.02, *P* = 0.383), indicating complete mediation.

In a similar model in which Δdepressive symptoms predicted ΔCT via ΔDNAm (Fig. 5b), we again observed a significant indirect effect (indirect = −0.05, *P* = 0.011), such that increases in depressive symptoms predicted greater DNAm reduction (*P* = 0.0099), which was in turn associated with greater cortical thinning. Here, the direct effect of Δdepressive symptoms on ΔCT was attenuated and non-significant (c′ = −0.06, *P* = 0.062), consistent with full mediation. In the reverse model where ΔDNAm predicted ΔCT through Δdepressive symptoms, we observed a significant indirect effect (indirect = 0.01, *P* = 0.044), indicating that adolescents showing greater DNAm reductions tended to report increases in depressive symptoms, which in turn were associated with greater cortical thinning. The direct association between ΔDNAm and ΔCT remained strong (c′ = 0.54, *P* = 0.0004), suggesting partial mediation.

Together, these models suggest that DNAm changes serve as a biological pathway linking affective distress and cannabis-use or binge drinking escalation to accelerated fronto-limbic cortical maturation, consistent with frameworks in which stress- and reward-related molecular mechanisms shape neurodevelopmental risk trajectories.

### Associations between cluster-defined DNAm patterns and psychopathology

Having identified coordinated DNAm patterns linking cortical maturation to psychopathology, we next examined cluster-specific DNAm signatures associated with substance use and depressive symptoms at ages 14 and 19.

#### Associations with substance use behaviours

As shown in Table S10 and Fig. 5c, d, associations with substance use differed across clusters and ages. At age 14, the only significant correlations (all *P*_*FDR*_ < 0.05) were positive associations between C4 and C9 and binge drinking. By age 19, more associations emerged, specifically with C5, C6, C9, C11, and C12. Notably, C6 and C11, which showed an overall decrease in methylation from ages 14 to 19, were negatively associated with smoking and cannabis use (Table S10); associations with cannabis use, remaining significant even after controlling for smoking. C6 was also negatively associated with binge drinking. This indicates that lower methylation levels in these clusters were linked to greater substance use involvement. In contrast, C12 showed positive associations with smoking and cannabis use. None of the smoking-related associations remained significant after controlling for cannabis use, reflecting unique effects of cannabis exposure to DNAm in these clusters. C9 was the only cluster associated with all substance-related behaviours, although none of the age 19 associations remained significant after controlling for smoking or cannabis use, suggesting a distinct molecular profile related to polysubstance exposure.

#### Associations with mental health symptoms

Two clusters displayed significant associations with depressive symptoms (*P*_*FDR*_ < 0.05; Fig. 5e; Table S10). C7 demonstrated a negative association with depressive symptoms at both ages, while C1 was positively associated with symptoms at age 19. Given the potential shared aetiology between depressive symptoms and negative psychotic experiences [18, 19], we also tested associations between ten brain-related DNAm clusters with dimensions of positive, negative and depressive psychotic symptoms at age 19. Significant associations were found for C7, which specifically associated with negative (*r* = −0.14, *P*_FDR_ = 0.031), but not depressive or positive psychosis symptoms (*P*_FDR_ > 0.05).

We next tested whether the associations between C1 and C7 and depressive symptoms replicated in the PPMI cohort. Both clusters again showed significant associations with depressive symptoms in this independent, older adult sample (*P*_one-tail_ < 0.005, Fig. 5f), demonstrating that these DNAm-symptom relationships are robust across cohorts, developmental stages, and clinical contexts. This cross-cohort replication suggests that C1 and C7 capture stable, developmentally persistent epigenetic variation relevant to depressive phenotype expression from adolescence through older adulthood.

### Genetic and epigenetic liability to depression and psychosis

To investigate the respective contributions of epigenetic and genetic factors to the risk for depression and psychosis, we complemented the above analyses with analyses of polygenic risk score (PRS) in IMAGEN. As expected, depression PRS showed significant (*P*_Bonferroni,one-tailed_ < 0.05) positive associations with depressive symptoms at both ages (age 14: *r* = 0.09, 95% CI [0.03, +∞), Power = 0.68; age 19: *r* = 0.16, 95% CI [0.09, +∞), Power = 0.97). Similarly, schizophrenia PRS was significantly associated with positive (*r* = 0.12, 95% CI [0.05, +∞), Power = 0.86), negative (*r* = 0.09, 95% CI [0.01, +∞), Power = 0.61), and depressive (*r* = 0.18, 95% CI [0.11, +∞), Power = 0.99) psychosis symptom dimensions at age 19.

We next tested whether the associations between C1 and C7 and mental health symptoms, as identified in the section above, remained significant when controlling for genetic effects (*i.e*., controlling the depression PRS or schizophrenia PRS in analyses of depressive and psychosis symptoms, respectively), and did observe so (Fig. 5g). This supports a model in which epigenetics (*i.e*., DNAm) and genetics represent distinct risk factors for depression and psychosis.

## Discussion

In this longitudinal study, we identified coordinated patterns of DNA methylation change during adolescence and demonstrated their relevance for brain maturation and mental health symptoms. Using data-driven clustering of epigenome-wide DNAm across ages 14 and 19, we derived an atlas of 18 developmentally co-regulated DNAm clusters. These clusters were robustly replicated across six independent samples of older adults, indicating that the developmental coordination captured in adolescence reflects stable organisational structure in the human methylome rather than cohort-specific effects. The atlas therefore provides a foundational resource for studying how epigenetic patterning unfolds across the lifespan.

Although prior DNAm atlas studies have been predominantly cross-sectional [38–40], our longitudinal design allowed us to characterise developmental trajectories of methylation change during adolescence, a neurodevelopmental window marked by pronounced cortical and subcortical reorganisation [11–13]. Crucially, we found that the magnitude and direction of DNAm change in specific brain-related clusters closely tracked the pace of cortical and subcortical maturation, particularly within prefrontal-limbic-striatal circuits supporting emotion regulation, reward learning, and stress sensitivity [14, 32, 34].

Several clusters enriched for genes expressed in the cortex and neurodevelopmental signalling pathways –including C3, C5, C6, C7, C9, C11 and C12– showed the strongest associations with longitudinal cortical thickness change. These clusters mapped onto two coordinated maturational profiles. First, greater DNAm reduction in clusters such as C12 and C5 (the latter enriched neuron differentiation pathways) was associated with greater cortical thinning in temporal and parietal association regions (*e.g*., entorhinal cortex, banks of the superior temporal sulcus). Given that DNA methylation typically represses transcription, this pattern is consistent with developmental derepression of neuronal differentiation and plasticity-related genes as paralimbic association cortices refine sensory-affective integration during adolescence [41–43]. Conversely, relative preservation of DNAm in clusters C6, C3, C7, C9 and C11 was linked to relative maintenance of cortical thickness in medial and lateral prefrontal regions (lateral/medial orbitofrontal cortex, pars opercularis, pars triangularis, and rostral and caudal middle frontal cortex) –regions central to valuation, cognitive control, and affective regulation [44, 45].

In addition to cortical changes, DNAm variation was also linked to subcortical maturation. Greater demethylation in clusters such as C7 and C6 was associated with more pronounced volume reductions in the amygdala, nucleus accumbens, thalamus, hippocampus and putamen, whereas relative maintenance of methylation in C1, C12, C11 and C8 was associated with relative volume preservation in the caudate and nucleus accumbens and attenuated lateral ventricular enlargement. Together, these findings indicate that the degree of methylation change in specific DNAm clusters systematically covaries with the tempo of structural brain maturation. The association between DNAm change and lateral ventricular enlargement likely reflects individual variation in the pace of normative neurodevelopmental tissue remodelling, as ventricular expansion in childhood and adolescence is a well-established marker of ongoing cortical-subcortical maturation [46], and later in life, of neurostructural vulnerability [47, 48]. Thus, the degree of DNAm change in these clusters may index individual differences in the tempo of fronto-limbic–striatal structural refinement, wherein greater demethylation corresponds to a faster maturational trajectory, while more stable methylation corresponds to relative structural preservation.

The mediation analyses further suggest a mechanistic role for DNAm in linking behavioural and affective experiences to neurodevelopment. In the case of substance use, increases in cannabis use and binge drinking predicted greater reductions in DNAm, which in turn were associated with greater cortical thinning, yielding significant indirect effects in the absence of direct behavioural-brain associations, consistent with a unidirectional, fully mediated pathway from substance use to brain maturation via DNAm change. In contrast, depressive symptoms showed evidence of bidirectional coupling with DNAm: increases in depressive symptoms predicted greater DNAm reduction, which subsequently related to greater cortical thinning, while DNAm reductions also predicted increases in depressive symptoms, partially accounting for additional variance in cortical change. Together, these findings support a model in which substance use drives neurodevelopmental change through epigenetic mechanisms, whereas affective symptom change and DNAm influence each other reciprocally, consistent with feedback regulation in stress- and reward-related molecular pathways [49].

Our cluster-specific analyses extend prior work linking DNAm to adolescent substance use [8, 50] and reveal both shared and substance-specific epigenetic signatures. C9 was associated with all substance-use behaviours at age 19, suggesting a general liability mechanism, whereas other clusters, such as C11 and C12 showed stronger specificity for cannabis or smoking. The neurobiological interpretation of these findings is strengthened by the observation that DNAm changes in these clusters relate to maturation of the prefrontal cortex, amygdala, caudate, and nucleus accumbens –regions central to reward learning and motivational salience [51–53].

Aligning with evidence of disrupted DNAm in schizophrenia [54–56] and depression [57, 58], two clusters, C1 and C7, showed consistent associations with depressive symptoms in both adolescence and older adulthood, including replication in PPMI. C7 was additionally associated with negative psychotic symptomatology, suggesting an epigenetic signature that may index transdiagnostic affective-blunting phenotypes [56] across disorders. The persistence of these associations following adjustment for polygenic risk scores supports models in which epigenetic variation constitutes an environmentally sensitive pathway, partly independent of inherited liability.

That DNA methylation variation within C1 and C7 tracked with the maturation of the amygdala and striatal nuclei is consistent with extensive evidence that these regions are highly plastic, environmentally responsive neural hubs involved in emotion regulation, stress responsivity, and reward processing [59]. Notably, C7, which loads on OFC and ACC and on amygdala/hippocampus, also shows negative associations with depressive symptoms (and with negative psychosis-like symptoms), aligning with cortical and subcortical loci repeatedly implicated in MDD [60, 61] and with OFC/ACC-centred depression subtypes [62]. The amygdala-prefrontal system is particularly sensitive to early life and social adversity, with epigenetic mechanisms proposed to mediate long-term effects on synaptic plasticity and circuit reactivity [63].

Taken together, these findings suggest that coordinated DNAm-brain maturation patterns index individual variation in the developmental tuning of affective and reward-circuitry systems. Clusters C1 and C7, in particular, capture stable, developmentally persistent epigenetic signatures that track with amygdala-striatal maturation and emerging depressive and negative-symptom phenotypes. Their reproducibility across cohorts and independence from genetic liability indicate potential utility as biomarkers capable of capturing environmentally shaped risk trajectories, informing stratification and early intervention strategies.

A key strength of this study is the integration of longitudinal DNA methylation, neuroimaging, and behavioural data within the same individuals across a developmentally critical window. The IMAGEN cohort provides a relatively large sample with repeated blood-based DNAm profiling and whole-brain MRI, alongside repeated assessment of depressive symptoms and substance use. This unique multimodal, longitudinal design enabled us to delineate coordinated trajectories of methylation change, cortical-subcortical maturation, and emerging psychopathology, offering rare insight into the dynamic coupling between molecular and neural development during adolescence. Nonetheless, several limitations warrant consideration. Although our longitudinal mediation analyses support a model in which substance-use and depressive symptoms escalation are statistically linked to DNAm change and cortical maturation, these associations cannot establish causality. The observed mediation effects suggest potential mechanistic pathways through which behavioural experiences may relate to neurodevelopmental trajectories, but the directionality of these processes may also reflect reverse causation or shared environmental influences. Effect sizes linking DNAm to behavioural symptoms were modest, in line with the polygenic and multifactorial nature of psychiatric traits. PRS-symptom associations were similarly modest in magnitude and should be interpreted as indexing broad liability rather than individual-level predictive accuracy. Replication in clinical or high-risk cohorts will be essential to determine sensitivity and predictive utility. Our metric of DNAm change reflects absolute β-value differences rather than proportional change, and while biologically interpretable, alternative representations may yield complementary information. Cannabis use was relatively uncommon at age 14; therefore early-adolescent associations should be interpreted cautiously, as non-significant findings at this age may reflect limited statistical power rather than absence of underlying effects. Finally, translation will be strengthened by future studies incorporating richer clinical phenotyping, higher temporal resolution, and experimental or interventional designs to further test mechanistic pathways.

In conclusion, this study presents the first longitudinal, whole-brain DNA methylation atlas of adolescence, revealing coordinated methylation-brain-behaviour coupling that links environmental experience to neurodevelopmental risk. By systematically integrating epigenetic, neuroimaging, and behavioural trajectories, it identifies reproducible DNAm signatures as potential biomarkers for early detection and prevention of affective and substance-use disorders in youth.

## Materials and methods

### Participants

#### IMAGEN cohort

Primary analyses were conducted in the IMAGEN study, a longitudinal population-based cohort followed from early to late adolescence [23]. The present study included 506 participants with DNAm and behavioural data available at both ages 14 and 19 (Table S1). Self-reported sex was used, reflecting biological assignment at birth. A detailed participant flow diagram for each analytical step is provided in Fig. S4.

#### Replication cohorts

To evaluate whether the longitudinal DNAm pattern identified in adolescence generalises across the lifespan, we examined two independent adult cohorts: (i) the Parkinson’s Progression Markers Initiative (PPMI) cohort [25] (*n* = 513; 32.9% female; mean age = 61.57 ± 10.04 years), consisting of individuals with Parkinson’s disease (PD, *n* = 329), PD and scans without evidence for dopaminergic deficit (SWEDD, *n* = 53), or neurologically healthy controls (*n* = 131) (Table S1), and (ii) the Alzheimer’s Disease Neuroimaging Initiative (ADNI) cohort (*n* = 606; 44.1% female; mean age = 73.16 ± 7.07 years), including participants with Alzheimer’s disease (AD, *n* = 72), mild cognitive impairment (MCI, *n* = 329), or healthy controls (*n* = 205) (Table S1). These datasets were used to test the robustness and reproducibility of the DNAm clustering solution.

### DNA methylation preprocessing

Genome-wide DNAm was assayed in peripheral whole blood using Illumina methylation microarrays (450 K and EPIC-850K) [5, 64], according to the manufacturer’s protocols. DNAm in IMAGEN was conducted in four processing waves: ages 14 DNAm included three waves (*n* = 238 Wave 1; *n* = 207 Wave 2; *n* = 61 Wave 4), and ages 19 DNAm included two (*n* = 325 Wave 3; *n* = 181 Wave 4). Waves 1 & 2 were processed with the 450 K, and waves 3 & 4 with the 850 K array. Participant allocation to each wave was randomised to avoid introducing systematic bias. For a subset of individuals (*n* = 61), DNAm at ages 14 and 19 was processed on the same platform (850 K array) within a single wave (wave 4) to specifically evaluate the impact of technical confounders such as platform and batch effects.

Preprocessing was performed using the Bioconductor package “minfi” (version: 1.46.0) [65]. To ensure comparability across arrays, we restricted analyses to the 372,582 CpG sites present on both the 450 K and 850 K platforms. Quantile normalisation (“preprocessQuantile”) was applied to IMAGEN, and Illumina normalisation (“preprocessIllumina”) to PPMI and ADNI, to accommodate case–control global methylation differences.

For IMAGEN DNAm analyses, we regressed out technical confounders (recruitment sites and waves) before conducting the WGCNA, hierarchical clustering, and pattern similarity analyses. All other DNAm analyses in IMAGEN were adjusted for sex, recruitment site, cell-type composition (first two PCs, capturing most of the variability [5, 6]), the first four DNAm PCs (capturing major technical and biological variance) [6], the first four genotype PCs (population stratification), and wave. For PPMI, we controlled for age, sex, race, education, recruitment site, cell types, and the first four DNAm PCs in analyses of depressive symptoms.

### Clustering analysis of DNAm data

A two-stage dimensionality reduction procedure was used for the DNAm data (workflow in Fig. S5).

#### Step 1: Consensus WGCNA modules

To minimise the impacts of technical confounders (*i.e*., platforms and waves) while retaining retained biological differences (i.e., changes related to age), consensus Weighted gene co-expression network analysis (WGCNA) [24] (R package “WGCNA”, version 1.72-5) was applied to DNAm matrices at ages 14 and 19 (506 ×372,582 CpGs for each timepoint), using the default and recommended settings. For adjacency matrices, soft-thresholding (β = 5) was selected using scale-free topology criteria for both age 14 and age 19 networks (Fig. S6). This resulted in the identification of 175 consensus modules, defined as sets of CpGs identified as co-methylated at both age 14 and age 19. We excluded CpGs assigned to Module 0 (*i.e*., unclustered CpGs) because such CpGs lack coherent co-methylation structure and therefore do not support biologically interpretable network-based inference [24, 66].

#### Step 2: Hierarchical clustering of longitudinal DNAm change

For each of the 175 modules, we computed the mean change in DNAm (ΔDNAm = age19 - age14). Hierarchical clustering of these ΔDNAm values was conducted using the R package “flashClust” (version: 1.01-2), using default settings. Euclidean distance [67] was used to quantify how dissimilar these ΔDNAm values were (i.e., how differently DNAm modules changed from ages 14 to 19), and average linkage to iteratively merge modules into higher-order clusters based on their mean pairwise distance, thresholding the number of clusters to 18 (*i.e*., 10% of 175 modules). These 18 higher-order clusters therefore capture shared developmental trajectories. Cluster-level DNAm scores (506 × 18 matrix) were computed as the mean DNAm across modules within each cluster. Batch effects (recruitment site, wave) were used as covariates during both the WGCNA and clustering steps.

### Robustness of the DNAm pattern

To assess the robustness of the identified DNAm pattern, we conducted a series of internal and external validation analyses. First, we compared our consensus-based clustering approach (modules required to be present at both ages 14 and 19) with a non-consensus approach in which WGCNA was applied directly to ΔDNAm values (Fig. S7). The non-consensus approach produced 190 modules; however, when we compared the similarity matrices of longitudinal change across subsets of the IMAGEN cohort, the consensus approach demonstrated significantly greater internal stability. Specifically, correlation matrices derived from the full cohort (450 K → 850 K data) and the subset of 61 adolescents measured on the same platform and wave (850 K → 850 K) showed higher similarity for the consensus structure than for the non-consensus structure (*r*_consensus_ = 0.86 vs. *r*_non-consensus_ = 0.79; Z_diff_ = 2.24; *P*_two-tailed_ = 0.025; Fig. S3), indicating that consensus-based clustering is more robust to technical heterogeneity.

To verify that longitudinal DNAm change within each module reflects coordinated biological signal rather than noise, we calculated the variance explained by the first principal component of CpG methylation change within each module. Across 175 modules, the first principal component accounted for an average of 43% of variance (95%CI: 0.40–0.45), demonstrating a strong, systematic pattern of co-methylation within modules.

External robustness and generalizability of the 18-cluster DNAm pattern were assessed in independent datasets (PPMI and ADNI). For each cohort, we computed cluster-wise similarity matrices (cluster × cluster Pearson correlations) and compared these to the IMAGEN longitudinal similarity matrix using the Mantel test (R package *vegan*, v2.6-10). Statistical significance was determined using 10,000 permutations (*P*_FDR_ < 0.05, FDR corrected). Significant Mantel correlations indicated that the inter-cluster co-variation structure was preserved across cohorts. Together, these analyses demonstrate that the 18-cluster DNAm architecture reflects biologically meaningful longitudinal co-variation rather than technical artefact.

### Functional and tissue-specific Enrichment analyses

Gene Ontology (GO) analysis identifies biological processes and molecular functions that are statistically overrepresented within a given gene set, while Kyoto Encyclopedia of Genes and Genomes (KEGG) analysis maps these genes to known biological pathways to infer potential functional mechanisms. Using the *gometh* function in the *missMethyl* R package (v1.34.0), we performed GO and KEGG enrichment for each DNAm cluster, accounting for probe-number bias. Genes were assigned to CpG sites based on proximity to the nearest transcription start site using the *getNearestTSS* function. Significance was determined using FDR correction (*P*_*FDR*_ < 0.05).

To assess tissue relevance, we applied the *teEnrichment* function of the R package“*TissueEnrich”* (v1.20.0) to test whether genes in each cluster were disproportionately expressed in specific tissues. Clusters were defined as brain-related if they showed (i) significantly enriched expression in brain tissues (*P*_*FDR*_ < 0.05) or (ii) major function (top ten) enriched for neurodevelopmental, synaptic, neuronal signalling, or neurodegeneration-related pathways (*P*_*FDR*_ < 0.05). These clusters were subsequently used in brain–epigenome and psychopathology analyses.

### Structural MRI processing

Structural MRI data in IMAGEN were acquired at eight sites using 3 T scanners from Siemens, Philips, General Electric, and Bruker, with harmonised acquisition protocols as previously described [23]. T1-weighted images were processed using the standard FreeSurfer pipeline (version 6.0.0), including intensity normalisation, skull stripping, surface reconstruction, and cortical parcellation [68]. Cortical thickness (CT), cortical surface area (SA), and regional grey matter volumes were derived from the Desikan–Killiany atlas (68 cortical parcels) and subcortical volumes were extracted using FreeSurfer’s *aseg* segmentation (16 subcortical regions) [69].

Quality control involved exclusion of regional measurements exceeding ±5 SD from the sample mean. Final analytic samples included *n* = 465 for SA, *n* = 463 for CT, and *n* = 443 for subcortical volume measures. Total intracranial volume (ICV), estimated via *aseg*, was included as a covariate in all structural analyses to account for global brain size differences.

### Phenotypic assessments

#### Substance use behaviours

Substance use was assessed using the European School Survey Project on Alcohol and Other Drugs (ESPAD; http://espad.org), a standardised and validated questionnaire widely used in European adolescent cohorts. Four substance use indicators were examined: cigarette smoking, cannabis use, alcohol use, and binge drinking. Participants reported the number of occasions they had engaged in each behaviour over their lifetime using integer response scales. Specifically, smoking was assessed with the item *“On how many occasions during your lifetime have you smoked cigarettes?”*; cannabis use was assessed as the number of occasions of using *“cannabis (grass, pot) or hashish”*; alcohol use as the number of occasions of consuming *“any alcoholic beverage”*; and binge drinking as the number of occasions of *“having five or more drinks in a row”* during the lifetime. Prevalence rates of each behaviour at ages 14 and 19 are provided in Table S1.

#### Socioeconomic stress (SES)

Parents’ reports of socioeconomic stress were measured using the family stress scale, as part of the Development and Well‐Being Assessment (DAWBA) [70]. A total score, which measures the degree to which unemployment, financial difficulties, home inadequacy, and neighbourhood problems made family life stressful, was used. High scores indicate more socioeconomic stress in the family. Only data at age 14 years was available for this measure.

#### Pubertal development (PD)

This was assessed at age 14 in IMAGEN using the Puberty Development Scale (PDS) [71], which provides a self-report measure of physical development based on Tanner stages. Total scores were calculated separately for males and from five characteristics: growth spurt in height, pubic hair, and skin change for both boys and girls; facial hair growth and voice change in boys only; and breast development and menarche in girls only.

### Psychiatric symptoms

#### Depressive symptoms

In IMAGEN, depressive symptoms at age 14 were assessed using the average score of self- and parents-reports from the development and well-being assessment (DAWBA) [70]. At age 19, depressive symptoms were assessed using the Adolescent Depression Rating Scale (ADRS), a 10-item self-report scale specifically developed to evaluate adolescent depression [72]. We used total scores, with higher scores reflecting higher symptom severity. To assess changes in depressive symptoms in longitudinal analyses, scores at each age were standardised (z = (X–mean)/SD), and longitudinal change was calculated as Δz (*i.e*., z_age19_−z_age14_).

In PPMI, depressive symptoms were assessed using the MDS-Unified Parkinson’s Disease Rating Scale (MDS-UPDRS) depressed mood item [73]. Only healthy controls were included to avoid confounding by Parkinson’s disease pathology.

#### Psychotic experiences

Three dimensions of psychotic-like experiences –positive, negative and depressive symptom domains– were assessed in IMAGEN at age 19 using the Community Assessment of Psychic Experience (CAPE) [55], a validated self-report questionnaire with good internal consistency [74]. Because psychotic experiences were not assessed at age 14, analyses of psychosis-related outcomes were restricted to age 19.

### Polygenic risk scores of psychiatric disorders

Polygenic risk scores (PRS) for major depressive disorder and schizophrenia were derived using summary statistics from the largest available GWAS meta-analyses for depression [75] and schizophrenia [76]. PRS were computed using PRSice (v1.25) with standard clumping (*r²* = 0.1, 250-kb window) [77] and across 100 *P*-value thresholds (0 to 0.5 in increments of 0.005). For downstream analyses, we selected the PRS derived using the conventional threshold of *P* < 0.05.

### Statistical analyses

All statistical analyses were conducted in R (version 4.3.1) or Matlab (version R2023a). False discovery rate (FDR) correction (*P*_FDR_ < 0.05) was applied to all analyses involving multiple parallel tests (*e.g*., enrichment, CCA, and behavioural associations across clusters or symptom domains), whereas hypothesis-driven confirmatory tests (*e.g*., replication analyses) were evaluated without further correction.

### Canonical correlation analyses (CCA)

These analyses involved the 10 DNAm clusters with evidence of central nervous system relevance (Clusters 1, 3, 4, 5, 6, 7, 8, 9, 11 and 12; totalling 276,980 CpGs) and structural MRI data from 68 cortical regions and 16 subcortical regions. We applied CCA [78, 79] (R package “CCA” version 1.2.2) to assess associations between averaged DNAm within clusters and MRI. Specifically, we investigated associations between changes in DNAm from ages 14 to 19 (*i.e*., △_DNAm_ = DNAm_19_-DNAm_14_) and changes in brain structure during the same period (*i.e*., △_brain_ = brain_19_-brain_14_). Three CCA were run: two investigated changes in cortical measures (*i.e*., surface area (SA) and average cortical thickness (CT)) within 68 cortical regions, and one, volumetric changes within 16 subcortical regions (SubCortVol). For each CCA, we first investigated the overall correlation between changes in DNAm within clusters with changes in the brain MRI, using permutation tests with 10,000 permutations to calculate exact P-values. FDR correction (*P*_*FDR*_ < 0.05) was applied throughout, including when (i) testing the overall significance, (ii) analysing across the full set of DNAm clusters within the first canonical component, and (iii) analysing across the full set of brain ROIs within the first canonical component.

Within the first canonical components, brain regions were considered most relevant if they showed the largest absolute canonical loading ( | *R* | )—indicating the strongest contribution to the shared DNAm-brain covariation pattern, and the smallest associated p-value within the CCA. A similar approach was taken when considering the most relevant DNAm clusters.

### Associations between DNAm, cortical maturation, and adolescent psychopathology

#### Longitudinal analyses

We examined whether developmental changes in substance use (binge drinking, alcohol use, cannabis use, and tobacco smoking) and mental health symptoms (depressive symptoms and psychotic experiences) were associated with the coordinated DNAm-brain maturation pattern. We focused on the first canonical variate pair from the CCAs, which captured the principal mode of covariation between DNAm change (Y₁; weighted composite of brain-related DNAm clusters) and cortical or subcortical maturation (X₁; weighted composite of cortical thickness (or subcortical volumes) changes across contributing regions). Linear regression models were estimated using change scores (Δ = age19 – age14), with ΔCCA brain or ΔCCA DNAm scores as the dependent variables.

#### Cross-sectional analyses

To characterise cluster-specific molecular signatures of psychopathology, we conducted linear regression analyses testing associations between average DNAm within each of the 10 brain-related clusters and behavioural measures across three domains: substance use, depressive symptoms, and psychotic symptom dimensions.

All analyses controlled for sex and recruitment site. Sensitivity models additionally controlled for pubertal development, socioeconomic status [80], and co-use of other substances to evaluate potential confounding (see Table S10). Multiple comparisons were addressed using FDR correction within each behavioural domain.

#### Replication in PPMI

Associations between DNAm clusters and depressive symptoms were tested in the PPMI dataset to assess reproducibility. Models controlled for age, sex, race, years of education, recruitment site, estimated blood cell composition, and the first four DNAm principal components. As the validation analysis tested a predefined directional hypothesis based on discovery results, one-tailed tests were applied to evaluate replication in the expected direction (*P*_one-tail_ < 0.05).

#### Mediation analyses

To further examine whether DNAm change may act as a mechanistic pathway linking behavioural and affective changes to cortical maturation, we conducted longitudinal mediation analyses. All variables were entered as change scores (Δ = age19–age14). Mediation models were implemented in MATLAB using the “MediationToolbox” [81], with non-parametric bootstrapping with 10,000 iterations to estimate indirect effects. We focused on the first canonical variate pair from the ΔCT CCA. Thus, these analyses tested whether ΔDNAm statistically mediated the associations between changes in depressive symptoms or substance-use behaviours and the shared cortical thinning pattern identified in the CCA. Conversely, we also tested whether depressive symptom change mediated the relationship between ΔDNAm and ΔCT, reflecting possible bidirectional coupling between DNAm and affect. To evaluate robustness, models were re-estimated controlling for change in cannabis use and baseline SES (age 14). The inclusion of these covariates did not alter the significance or direction of indirect effects, indicating that the mediation pathways observed were not attributable to socioeconomic or substance-use confounding.

### Associations with Polygenic Risk Scores

Associations between PRS and behavioural phenotypes were tested using linear regression. One-tailed tests were used where the expected direction of effect was specified a priori based on prior literature (*i.e*., higher PRS predicting higher symptom severity). To quantify uncertainty, 95% confidence intervals for correlation coefficients were estimated using 10,000 bootstrap resamples to reflect the one-sided hypothesis. Achieved statistical power was estimated using G*Power (v3.1) [82]. Because only two PRS–depressive symptom tests were conducted (ages 14 and 19), Bonferroni correction was applied to control Type I error. For schizophrenia, PRS associations with three psychosis symptom dimensions, FDR correction was applied within the domain.

### Ethical approval

The IMAGEN project received approval from the local ethics research committees at each research site [23]: King’s College London, University of Nottingham, Trinity College Dublin, University of Heidelberg, Technische Universität Dresden, Commissaria de l’Energie Atomique et aux Energies Alternatives and University Medical Center. Informed consent was provided by all participants or by a parent/guardian. All methods were performed in accordance with the committees’ relevant guidelines and regulations.

## Supplementary information

Supplementary Materials

## Data Availability

The IMAGEN data is available from a dedicated database at https://imagen2.cea.fr. The PPMI data is available from the website (https://www.ppmi-info.org/). The ADNI data is available from the website (https://adni.loni.usc.edu/).

## Code availability

Clustering analyses were conducted using the R package “WGCNA” (version 1.72-5). Functional enrichment analyses were performed with the R package “missMethyl” (version 1.34.0). Tissue-specific enrichment was assessed using the R package “TissueEnrich” (version 1.20.0). T1-weighted MRI images were processed with FreeSurfer (version 6.0; http://surfer.nmr.mgh.harvard.edu/). Canonical correlation analysis (CCA) was carried out using the R package “CCA” (version 1.2.2). CpGs corresponding to the 18 DNAm Clusters are available on Github (https://github.com/dichen27/DNAm_pattern/tree/main/Methylation_Running/Cluster18).
