# Supplementary material for: A longitudinal DNA methylation atlas and its link to brain structure and mental health
Source: Mol Psychiatry. 2026 Mar 31;31(8):4535–49. doi: 10.1038/s41380-026-03554-y (PMC13364678; doi:10.1038/s41380-026-03554-y)
Supplement: Supplementary file 1 — Supplementary Materials [file 41380_2026_3554_MOESM1_ESM.pdf]

Supplementary Materials

Part-1: Supplemental Tables 2

Table S1. Samples characteristics 2

Table S2. Composition and chromosomal distribution of the DNAm clusters 3

Table S3. DNAm levels within identified clusters, and their associations with sex, PD, and SES 4

Table S4. Tissue-specific gene expression enrichment analyses 5

Table S5. Functional enrichment analyses 7

Table S6. Developmental changes in cortical and subcortical morphology from ages 14 to 19 11

Table S7. CCA results: DNAm clusters contributions to the first component 13

Table S8. CCA results: Brain regions contributions to the first component 14

Table S9. Longitudinal associations between mental health, DNAm and MRI 17

Table S10. Associations between DNAm and psychopathology 18

Part-2: Supplementary Figures 21

Figure S1. Schematic overview of the study. 21

Figure S2. Genomic distribution plot of DNAm clusters. 39

Figure S3. Robustness of the consensus approach. 40

Figure S4. Flowchart of participants included. 41

Figure S5. Flowchart highlighting the dimensionality reduction. 42

Figure S6. Determination of the soft-threshold parameters in the WGCNA. 43

Figure S7. Soft-threshold of DNAm changes for no-consensus approach. 44

Part-1: Supplemental Tables

Table S1. Samples characteristics

Datasets	IMAGEN					PPMI			ADNI		
	Age 14	Age 19	Age 19 - Age 14								
Diagnosis	n.a	n.a				PD	SWEDD	HC	PD	MCI	HC
N	506	506				329	53	131	72	329	205
Sex: N males/females	240/266	240/266				218/111	33/20	93/38	50/22	187/142	102/103
Age: mean (SD)	14.43 (0.40)	18.94 (0.72)				61.72 (9.63)	60.79 (10.10)	61.50 (11.06)	74.31 (8.12)	71.98 (7.28)	74.66 (5.91)
Mental health symptoms											
Depressive symptoms: mean (SD),variability*	0.48 (0.77), 1.61	1.41 (2.17), 1.54				n.a	n.a	0.15 (0.40), 2.62	n.a		
Psychotic symptoms: mean (SD)	n.a	118.50 (22.48)				n.a			n.a		
Substance use behaviours (lifetime frequency)			Cohen's *d*	*t*	*P*	n.a			n.a		
Alcohol use: mean (SD)	1.95 (1.73)	5.14 (1.39)	2.08	32.77	< 0.0001						
Alcohol use: prevalance	75.64%	97.98%									
Binge drinking: mean (SD)	0.52 (1.15)	3.52 (1.82)	2.00	31.52	< 0.0001						
Binge drinking: prevalance	31.68%	86.09%									
Tobacco smoking: mean (SD)	0.68 (1.52)	2.88 (2.52)	1.07	16.78	< 0.0001						
Tobacco smoking: prevalance	25.54%	69.96%									
Cannabis use: mean (SD)	0.12 (0.60)	1.73 (2.21)	1.02	16.01	< 0.0001						
Cannabis use: prevalance	5.94%	51.21%									

*variability = coefficient of variation

Abbreviations: HC, Healthy controls; PD, Parkinson's Disease; SWEDD, scans without evidence for dopaminergic deficit; MCI, mild cognitive impairment; SD: standard deviation

Table S2. Composition and chromosomal distribution of the DNAm clusters

Cluster number	Number of modules	Number of CpGs	Chromosomal distribution of CpGs within each cluster																					
			Chr 1	Chr 2	Chr 3	Chr 4	Chr 5	Chr 6	Chr 7	Chr 8	Chr 9	Chr 10	Chr 11	Chr 12	Chr 13	Chr 14	Chr 15	Chr 16	Chr 17	Chr 18	Chr 19	Chr 20	Chr 21	Chr 22
C1	30	103204	9.4%	8.3%	6.3%	5.3%	5.7%	8.5%	6.7%	4.9%	1.9%	5.6%	5.6%	5.4%	3.3%	3.3%	3.3%	3.7%	4.7%	1.2%	3.4%	1.6%	0.8%	1.2%
C2	1	23511	11.0%	6.9%	5.4%	3.4%	4.3%	7.1%	5.4%	4.1%	2.3%	4.5%	7.0%	5.5%	2.2%	3.2%	2.8%	5.2%	7.9%	1.0%	5.4%	2.4%	1.0%	2.0%
C3	17	30123	9.8%	6.6%	5.4%	4.1%	4.5%	6.4%	5.8%	3.9%	1.4%	4.8%	6.5%	5.3%	2.3%	3.3%	3.3%	5.3%	7.0%	1.6%	6.8%	2.9%	1.0%	2.3%
C4	18	29907	10.5%	6.1%	4.2%	3.2%	4.3%	6.7%	6.6%	4.2%	2.7%	4.6%	6.7%	4.3%	1.9%	3.1%	2.9%	6.4%	7.0%	1.1%	7.5%	2.8%	1.0%	2.4%
C5	13	40229	9.7%	7.3%	5.8%	4.6%	5.7%	6.4%	5.2%	4.0%	2.1%	4.8%	6.2%	5.5%	2.4%	3.4%	3.4%	3.9%	6.1%	1.7%	6.2%	2.9%	0.8%	2.0%
C6	6	12653	11.2%	7.3%	4.9%	3.2%	4.9%	6.3%	5.6%	4.3%	2.4%	6.1%	7.7%	4.7%	2.2%	3.4%	3.6%	5.0%	6.5%	0.9%	3.9%	2.5%	1.1%	2.2%
C7	9	18769	9.7%	8.1%	5.7%	5.4%	6.1%	8.8%	6.2%	4.8%	2.0%	5.2%	5.9%	6.1%	3.1%	3.3%	3.5%	3.1%	4.3%	1.5%	3.4%	2.0%	0.7%	1.1%
C8	9	12103	9.9%	7.7%	6.0%	3.9%	4.9%	6.4%	4.3%	3.3%	2.6%	4.2%	6.1%	5.6%	2.4%	3.5%	4.0%	4.3%	6.6%	1.6%	6.9%	2.6%	0.9%	2.4%
C9	23	15475	10.9%	6.7%	5.3%	3.5%	4.6%	6.9%	4.4%	3.1%	2.0%	4.4%	6.1%	5.4%	1.8%	3.5%	3.2%	4.8%	7.2%	1.2%	9.0%	2.7%	1.0%	2.4%
C10	11	9769	9.8%	7.2%	5.8%	3.5%	4.4%	6.9%	5.3%	3.9%	1.5%	4.2%	6.2%	5.4%	1.8%	3.2%	3.4%	4.5%	7.3%	1.6%	7.6%	3.0%	1.0%	2.6%
C11	4	5404	10.5%	8.6%	5.9%	3.7%	5.1%	6.6%	5.4%	4.1%	2.1%	5.4%	7.2%	5.4%	2.8%	4.0%	4.2%	3.7%	6.0%	1.1%	3.0%	2.6%	1.2%	1.6%
C12	16	9113	9.4%	5.9%	3.7%	4.0%	3.9%	6.3%	7.3%	4.5%	1.8%	5.2%	6.3%	5.2%	2.9%	3.0%	2.6%	7.3%	7.7%	1.2%	7.8%	1.8%	0.9%	1.6%
C13	2	655	10.5%	6.7%	5.8%	3.1%	5.0%	6.6%	7.3%	3.8%	2.4%	4.0%	6.3%	6.4%	2.8%	1.8%	4.4%	4.0%	5.8%	1.1%	6.7%	2.4%	0.9%	2.1%
C14	6	1147	11.0%	6.8%	5.4%	4.4%	4.3%	6.0%	5.5%	4.5%	2.0%	4.5%	7.5%	4.5%	1.8%	2.7%	3.0%	3.4%	6.1%	1.8%	7.0%	4.6%	0.9%	2.3%
C15	2	718	9.9%	8.4%	4.9%	5.2%	6.6%	7.9%	3.1%	4.6%	1.5%	5.6%	7.0%	6.1%	2.1%	3.2%	3.5%	4.5%	5.7%	1.3%	5.2%	1.8%	0.6%	1.7%
C16	3	824	11.8%	6.8%	5.1%	4.3%	5.1%	4.1%	5.6%	4.0%	2.4%	3.5%	6.3%	4.7%	1.8%	4.5%	3.3%	4.0%	7.0%	1.5%	9.0%	4.0%	0.4%	0.9%
C17	2	226	9.7%	6.6%	6.6%	4.4%	4.4%	8.9%	4.9%	3.5%	2.2%	4.4%	5.8%	6.6%	1.3%	1.8%	3.5%	3.5%	9.3%	0.4%	5.8%	2.2%	1.8%	2.2%
C18	3	253	13.4%	5.5%	4.0%	1.6%	8.7%	7.1%	7.5%	2.8%	1.6%	2.8%	7.1%	4.0%	4.0%	3.2%	4.0%	6.3%	6.3%	0.4%	5.9%	1.6%	0.4%	2.0%

Table S3. DNAm levels within identified clusters, and their associations with sex, PD, and SES

DNAm cluster	DNAm levels				Associations with sex*						Associations with PD**		Associations with SES***	
	Age 14		Age 19		Age 14			Age 19			Age 14		Age 14	
	mean	std	mean	std	*t*	Cohen's *d*	P_{FDR}	*t*	Cohen's *d*	P_{FDR}	*r*	P_{FDR}	*r*	P_{FDR}
C1	0.8208	0.0309	0.8148	0.0204	5.56	0.51	**2.74E-07**	2.35	0.21	**4.29E-02**	-0.05	0.7816	0.02	0.8377
C2	0.506	0.0264	0.4902	0.0203	7.34	0.67	**8.33E-12**	4.00	0.36	**4.37E-04**	0.01	0.9268	0.05	0.6033
C3	0.2099	0.0028	0.2035	0.0013	-0.97	-0.09	0.4632	3.83	0.35	**6.47E-04**	-0.01	0.9268	-0.04	0.6817
C4	0.7025	0.016	0.6878	0.0112	-10.50	-0.95	**0.00**	6.65	0.60	**1.45E-09**	0.05	0.7816	0.06	0.4598
C5	0.102	0.0099	0.1056	0.0081	0.22	0.02	0.9300	3.04	0.28	**7.57E-03**	-0.07	0.5916	0.02	0.8377
C6	0.8197	0.0194	0.7935	0.0145	2.60	0.24	**2.89E-02**	1.39	0.13	0.2306	-0.02	0.9268	-0.07	0.3750
C7	0.3599	0.0202	0.3394	0.012	-0.09	-0.01	0.9593	1.87	0.17	0.1123	0.03	0.9029	-0.03	0.6817
C8	0.0462	0.0073	0.0513	0.0035	2.82	0.26	**1.83E-02**	-3.71	-0.34	**8.31E-04**	-0.09	0.5916	0.00	0.9717
C9	0.0799	0.0057	0.0715	0.0023	-0.68	-0.06	0.6033	1.28	0.12	0.2579	0.04	0.7816	0.08	0.3750
C10	0.0541	0.003	0.051	0.0019	-0.97	-0.09	0.4632	1.12	0.10	0.3164	-0.01	0.9268	0.06	0.4598
C11	0.8709	0.0148	0.8508	0.0134	4.78	0.43	**1.04E-05**	-1.03	-0.09	0.3427	-0.07	0.5916	-0.05	0.6033
C12	0.7785	0.0101	0.761	0.0082	2.49	0.23	**3.41E-02**	1.96	0.18	0.1020	-0.07	0.5916	-0.15	**1.35E-02**
C13	0.5845	0.0473	0.5404	0.0179	0.67	0.06	0.6033	1.74	0.16	0.1251	-0.02	0.9268	-0.11	0.0928
C14	0.2638	0.0068	0.2647	0.0065	-1.63	-0.15	0.2285	4.56	0.41	**5.96E-05**	0.00	0.9473	0.04	0.6817
C15	0.2458	0.0051	0.2456	0.0032	1.58	0.14	0.2285	-1.73	-0.16	0.1251	-0.01	0.9268	-0.02	0.8377
C16	0.0697	0.0152	0.0525	0.0038	-1.20	-0.11	0.3763	0.19	0.02	0.8488	0.03	0.9029	0.00	0.9717
C17	0.1312	0.0079	0.1271	0.0086	-0.05	0.00	0.9593	0.36	0.03	0.7648	0.02	0.9268	-0.01	0.9276
C18	0.5432	0.017	0.5494	0.0056	1.36	0.12	0.3130	2.59	0.24	**2.52E-02**	-0.05	0.7816	-0.12	0.0928

*Positive *t*- values indicate higher methylation in males compared to females. Positive *r*- values indicate higher methylation associated with more advanced pubertal development (**) or higher socioeconomic stress (***)

Table S4. Tissue-specific gene expression enrichment analyses

Tissue	Number of tissue-specific genes	*P*	P_{FDR}
Cluster 1			
Cerebral Cortex	1259	6.22E-05	2.18E-03
Cluster 2			
Spleen	191	1.41E-09	3.82E-08
Lymph Node	160	2.18E-09	3.82E-08
Bone Marrow	179	5.96E-09	6.95E-08
Appendix	112	5.14E-06	4.49E-05
Adipose Tissue	93	3.01E-03	2.10E-02
Cluster 3			
Cerebral Cortex	932	9.31E-08	3.26E-06
Cluster 4			
Cerebral Cortex	893	1.38E-45	4.84E-44
Skeletal Muscle	220	7.35E-19	1.29E-17
Skin	277	5.76E-12	6.72E-11
Heart Muscle	126	7.77E-08	6.31E-07
Adipose Tissue	107	9.01E-08	6.31E-07
Adrenal Gland	148	2.69E-06	1.57E-05
Prostate	113	3.34E-05	1.67E-04
Esophagus	141	4.23E-05	1.85E-04
Small Intestine	183	2.38E-04	9.25E-04
Lung	106	2.92E-04	1.02E-03
Duodenum	171	7.64E-04	2.43E-03
Ovary	78	1.37E-03	3.99E-03
Thyroid Gland	141	2.06E-03	5.56E-03
Endometrium	68	2.58E-03	6.45E-03
Smooth Muscle	52	3.09E-03	7.03E-03
Seminal Vesicle	94	3.21E-03	7.03E-03
Kidney	172	3.82E-03	7.87E-03
Placenta	175	1.38E-02	2.68E-02
Colon	83	2.07E-02	3.82E-02
Gallbladder	76	2.46E-02	4.10E-02
Spleen	152	2.46E-02	4.10E-02
Cluster 5			
Cerebral Cortex	1128	1.98E-60	6.94E-59
Adrenal Gland	181	1.90E-05	3.33E-04
Endometrium	88	7.69E-04	8.98E-03

Cluster 6			
Cerebral Cortex	643	7.26E-41	2.54E-39
Skin	218	4.32E-17	7.55E-16
Esophagus	119	2.64E-10	3.09E-09
Heart Muscle	99	7.04E-10	6.16E-09
Lung	91	7.93E-09	5.55E-08
Skeletal Muscle	139	1.64E-08	9.56E-08
Small Intestine	144	7.03E-08	3.51E-07
Duodenum	137	9.29E-08	4.07E-07
Kidney	140	2.38E-07	9.27E-07
Adipose Tissue	73	1.81E-05	6.34E-05
Thyroid Gland	108	2.30E-05	7.32E-05
Rectum	71	6.31E-05	1.75E-04
Adrenal Gland	101	6.49E-05	1.75E-04
Cervix, uterine	67	8.12E-05	1.89E-04
Seminal Vesicle	73	8.12E-05	1.89E-04
Colon	68	9.20E-05	2.01E-04
Gallbladder	63	1.03E-04	2.12E-04
Ovary	59	1.48E-04	2.87E-04
Liver	152	2.13E-04	3.93E-04
Salivary Gland	40	6.43E-04	1.13E-03
Lymph Node	94	1.35E-03	2.06E-03
Spleen	112	1.35E-03	2.06E-03
Placenta	126	1.35E-03	2.06E-03
Stomach	61	8.63E-03	1.25E-02
Endometrium	46	8.90E-03	1.25E-02
Tonsil	65	9.39E-03	1.26E-02
Fallopian Tube	147	2.24E-02	2.90E-02
Smooth Muscle	33	3.56E-02	4.45E-02
Cluster 7			
Cerebral Cortex	787	7.55E-17	2.64E-15
Cluster 11			
Cerebral Cortex	412	4.77E-26	1.67E-24
Kidney	96	3.54E-06	6.20E-05
Adrenal Gland	75	8.12E-06	9.47E-05
Placenta	93	6.70E-05	3.91E-04
Skeletal Muscle	86	6.70E-05	3.91E-04
Small Intestine	91	6.70E-05	3.91E-04
Lung	54	2.27E-04	1.14E-03
Cervix, uterine	45	7.29E-04	2.84E-03
Colon	46	7.29E-04	2.84E-03

Ovary	40	1.02E-03	3.35E-03
Heart Muscle	54	1.05E-03	3.35E-03
Gallbladder	41	1.91E-03	4.46E-03
Prostate	51	1.91E-03	4.46E-03
Rectum	45	1.91E-03	4.46E-03
Skin	107	1.91E-03	4.46E-03
Duodenum	78	2.29E-03	5.01E-03
Thyroid Gland	66	2.46E-03	5.07E-03
Esophagus	61	4.20E-03	7.99E-03
Stomach	43	4.34E-03	7.99E-03
Salivary Gland	25	1.12E-02	1.97E-02
Cluster 12			
Cerebral Cortex	453	4.94E-10	1.73E-08

Table S5. Functional enrichment analyses (top ten)

		ONTOLOGOGY	TERM (top ten)	N	DE	P	P_{FDR}
GO enrichment	*Cluster 1*						
	GO:0043167	MF	ion binding	5616	5046	9.32E-09	0.000211992
	GO:0043168	MF	anion binding	2264	2058	6.19E-07	0.006361399
	GO:0000166	MF	nucleotide binding	2010	1831	8.44E-07	0.006361399
	GO:0032559	MF	adenyl ribonucleotide binding	1449	1329	1.45E-06	0.006361399
	GO:0000902	BP	cell morphogenesis	876	816	1.63E-06	0.006361399
	GO:0005524	MF	ATP binding	1395	1280	1.81E-06	0.006361399
	GO:1901265	MF	nucleoside phosphate binding	2011	1831	1.96E-06	0.006361399
	GO:0030554	MF	adenyl nucleotide binding	1546	1415	3.34E-06	0.009488168
	GO:0032553	MF	ribonucleotide binding	1780	1623	4.95E-06	0.012505243
	GO:0017076	MF	purine nucleotide binding	1861	1695	6.51E-06	0.014811088
	Cluster 2						
	GO:0002376	BP	immune system process	2280	1193	1.51E-21	3.44E-17
	GO:0001775	BP	cell activation	1030	596	4.93E-19	5.60E-15
	GO:0006955	BP	immune response	1529	797	9.42E-19	7.14E-15
	GO:0045321	BP	leukocyte activation	889	520	3.70E-18	2.10E-14
	GO:0046649	BP	lymphocyte activation	727	426	1.86E-14	8.45E-11
	GO:0050865	BP	regulation of cell activation	624	368	2.66E-14	9.98E-11
	GO:0002682	BP	regulation of immune system process	1378	729	3.07E-14	9.98E-11
	GO:0002250	BP	adaptive immune response	416	246	3.37E-13	9.15E-10
	GO:0002684	BP	positive regulation of immune system process	946	517	3.62E-13	9.15E-10
	GO:0048583	BP	regulation of response to stimulus	3825	1940	4.30E-13	9.78E-10
	Cluster 4						

	GO:0071944	CC	cell periphery	5469	2699	9.74E-13	2.22E-08
	GO:0098590	CC	plasma membrane region	1226	686	5.78E-09	6.57E-05
	GO:0005886	CC	plasma membrane	5015	2439	9.39E-09	7.12E-05
	GO:1901702	MF	salt transmembrane transporter activity	536	321	1.30E-08	7.37E-05
	GO:0030054	CC	cell junction	2099	1151	2.13E-08	9.70E-05
	GO:0022836	MF	gated channel activity	289	187	7.36E-08	2.39E-04
	GO:0022839	MF	monoatomic ion gated channel activity	289	187	7.36E-08	2.39E-04
	GO:0030312	CC	external encapsulating structure	525	302	9.99E-08	2.84E-04
	GO:0043005	CC	neuron projection	1264	713	1.20E-07	2.87E-04
	GO:0031012	CC	extracellular matrix	524	301	1.26E-07	2.87E-04
	Cluster 5						
	GO:0007399	BP	nervous system development	2374	1778	6.34E-25	1.44E-20
	GO:0048731	BP	system development	3811	2702	9.01E-22	1.02E-17
	GO:0007275	BP	multicellular organism development	4462	3121	1.70E-21	1.29E-17
	GO:0048699	BP	generation of neurons	1368	1061	1.76E-20	7.55E-17
	GO:0022008	BP	neurogenesis	1591	1217	1.92E-20	7.55E-17
	GO:0030182	BP	neuron differentiation	1299	1012	1.99E-20	7.55E-17
	GO:0009653	BP	anatomical structure morphogenesis	2603	1854	2.26E-14	6.71E-11
	GO:1990837	MF	sequence-specific double-stranded DNA binding	1362	1002	2.36E-14	6.71E-11
	GO:0043565	MF	sequence-specific DNA binding	1463	1067	1.31E-13	3.31E-10
	GO:0003690	MF	double-stranded DNA binding	1453	1057	1.72E-13	3.59E-10
	Cluster 6						
	GO:0071944	CC	cell periphery	5469	1833	1.75E-38	3.99E-34
	GO:0005886	CC	plasma membrane	5015	1655	7.35E-30	8.36E-26
	GO:0003008	BP	system process	2125	728	1.25E-17	9.45E-14
	GO:0050877	BP	nervous system process	1373	475	1.58E-14	8.97E-11
	GO:0038023	MF	signaling receptor activity	1307	430	4.43E-13	1.68E-09
	GO:0060089	MF	molecular transducer activity	1307	430	4.43E-13	1.68E-09
	GO:0004888	MF	transmembrane signaling receptor activity	1105	364	1.74E-12	5.65E-09
	GO:0098590	CC	plasma membrane region	1226	456	6.93E-12	1.97E-08
	GO:0007600	BP	sensory perception	863	288	2.24E-11	5.29E-08
	GO:0005576	CC	extracellular region	3985	1159	2.33E-11	5.29E-08
	Cluster 8						
	GO:0005654	CC	nucleoplasm	3871	2094	2.76E-49	6.28E-45
	GO:0031981	CC	nuclear lumen	4528	2256	5.37E-37	6.10E-33
	GO:0005634	CC	nucleus	7132	3398	3.37E-36	2.55E-32
	GO:0031974	CC	membrane-enclosed lumen	5593	2703	1.71E-34	6.47E-31
	GO:0043233	CC	organelle lumen	5593	2703	1.71E-34	6.47E-31
	GO:0070013	CC	intracellular organelle lumen	5593	2703	1.71E-34	6.47E-31
	GO:1902494	CC	catalytic complex	1593	904	1.89E-30	6.13E-27

	GO:0043231	CC	intracellular membrane-bounded organelle	11296	5085	2.53E-27	7.20E-24
	GO:0044260	BP	cellular macromolecule metabolic process	3057	1565	2.72E-26	6.88E-23
	GO:0140513	CC	nuclear protein-containing complex	1128	659	8.40E-26	1.91E-22
	Cluster 10						
	GO:0005654	CC	nucleoplasm	3871	1787	9.14E-28	2.08E-23
	GO:0031981	CC	nuclear lumen	4528	1933	3.28E-22	3.72E-18
	GO:0005634	CC	nucleus	7132	2921	1.70E-20	1.29E-16
	GO:0031974	CC	membrane-enclosed lumen	5593	2305	1.02E-18	3.86E-15
	GO:0043233	CC	organelle lumen	5593	2305	1.02E-18	3.86E-15
	GO:0070013	CC	intracellular organelle lumen	5593	2305	1.02E-18	3.86E-15
	GO:0044260	BP	cellular macromolecule metabolic process	3057	1355	2.41E-18	7.83E-15
	GO:0140513	CC	nuclear protein-containing complex	1128	565	2.99E-17	8.50E-14
	GO:0003676	MF	nucleic acid binding	3812	1615	3.64E-16	9.20E-13
	GO:0044237	BP	cellular metabolic process	9039	3570	4.19E-15	9.52E-12
	Cluster 11						
	GO:0071944	CC	cell periphery	5469	1043	3.94E-15	8.97E-11
	GO:0005886	CC	plasma membrane	5015	940	1.21E-11	1.38E-07
	GO:0098590	CC	plasma membrane region	1226	269	1.61E-06	1.22E-02
	GO:0006816	BP	calcium ion transport	435	109	3.00E-06	1.71E-02
	GO:0031012	CC	extracellular matrix	524	126	9.04E-06	3.30E-02
	GO:0030312	CC	external encapsulating structure	525	126	9.63E-06	3.30E-02
	GO:0019731	BP	antibacterial humoral response	53	19	1.02E-05	3.30E-02
	GO:0030054	CC	cell junction	2099	439	1.16E-05	3.30E-02
	GO:0005576	CC	extracellular region	3985	668	1.39E-05	3.50E-02
	GO:0070588	BP	calcium ion transmembrane transport	344	89	1.58E-05	3.59E-02
	Cluster 15						
	GO:0098687	CC	chromosomal region	371	34	6.25E-07	0.01420229
KEGG pathways	*Cluster 2*						
	hsa04060		Cytokine-cytokine receptor interaction	269	142	7.89E-08	2.81E-05
	hsa04640		Hematopoietic cell lineage	90	57	1.39E-05	2.48E-03
	hsa04061		Viral protein interaction with cytokine and cytokine receptor	96	52	3.74E-05	4.44E-03
	hsa04064		NF-kappa B signaling pathway	95	61	8.98E-05	7.99E-03
	hsa04066		HIF-1 signaling pathway	104	69	1.39E-04	9.92E-03
	hsa04380		Osteoclast differentiation	124	77	1.86E-04	1.10E-02
	hsa05146		Amoebiasis	98	61	5.00E-04	2.54E-02
	hsa04142		Lysosome	127	75	7.87E-04	3.08E-02
	hsa05340		Primary immunodeficiency	32	23	8.22E-04	3.08E-02
	hsa04072		Phospholipase D signaling pathway	144	92	8.64E-04	3.08E-02
	Cluster 3						
	hsa05012		Parkinson disease	241	184	2.36E-06	4.74E-04

	hsa04110		Cell cycle	152	124	2.66E-06	4.74E-04
	hsa05010		Alzheimer disease	353	266	8.56E-06	1.02E-03
	hsa04714		Thermogenesis	212	161	1.97E-05	1.75E-03
	hsa05022		Pathways of neurodegeneration - multiple diseases	443	325	4.29E-05	3.06E-03
	hsa03008		Ribosome biogenesis in eukaryotes	68	56	8.43E-05	5.00E-03
	hsa00190		Oxidative phosphorylation	116	88	2.41E-04	1.05E-02
	hsa05224		Breast cancer	145	118	2.81E-04	1.05E-02
	hsa04150		mTOR signaling pathway	152	121	2.95E-04	1.05E-02
	hsa05014		Amyotrophic lateral sclerosis	336	240	2.96E-04	1.05E-02
	Cluster 7						
	hsa05034		Alcoholism	164	94	3.38447E-05	1.20E-02
	Cluster 8						
	hsa04110		Cell cycle	152	100	4.19E-08	1.49E-05
	hsa03040		Spliceosome	124	78	6.92E-06	8.57E-04
	hsa05220		Chronic myeloid leukemia	74	53	8.16E-06	8.57E-04
	hsa04120		Ubiquitin mediated proteolysis	134	85	9.63E-06	8.57E-04
	hsa05014		Amyotrophic lateral sclerosis	336	181	1.28E-05	9.08E-04
	hsa03013		Nucleocytoplasmic transport	101	64	2.14E-05	1.17E-03
	hsa05210		Colorectal cancer	85	58	2.30E-05	1.17E-03
	hsa05169		Epstein-Barr virus infection	184	107	5.91E-05	2.63E-03
	hsa00020		Citrate cycle (TCA cycle)	28	22	7.45E-05	2.95E-03
	hsa05212		Pancreatic cancer	73	50	1.07E-04	3.50E-03
	Cluster 9						
	hsa03010		Ribosome	125	88	4.54E-10	1.62E-07
	hsa04110		Cell cycle	152	109	4.53E-09	8.06E-07
	hsa03082		ATP-dependent chromatin remodeling	108	77	1.30E-07	1.55E-05
	hsa03040		Spliceosome	124	84	3.34E-06	2.98E-04
	hsa05203		Viral carcinogenesis	186	118	4.60E-06	3.27E-04
	hsa04218		Cellular senescence	152	101	2.36E-05	1.40E-03
	hsa05010		Alzheimer disease	353	209	4.10E-05	2.04E-03
	hsa05012		Parkinson disease	241	144	4.58E-05	2.04E-03
	hsa05216		Thyroid cancer	37	30	1.42E-04	5.61E-03
	hsa05161		Hepatitis B	146	93	1.88E-04	6.71E-03
	Cluster 10						
	hsa03082		ATP-dependent chromatin remodeling	108	64	1.80E-06	0.000640611
	hsa03013		Nucleocytoplasmic transport	101	60	5.22E-06	0.000929616
	hsa04110		Cell cycle	152	84	2.96E-05	0.003517979
	hsa05203		Viral carcinogenesis	186	94	1.84E-04	0.016357335
	hsa03040		Spliceosome	124	66	3.19E-04	0.020724621
	hsa04218		Cellular senescence	152	82	3.89E-04	0.020724621

	hsa03015		mRNA surveillance pathway	84	48	4.08E-04	0.020724621
	hsa05100		Bacterial invasion of epithelial cells	76	46	4.80E-04	0.021352366
	hsa03083		Polycomb repressive complex	75	45	8.94E-04	0.035379627

Table S6. Developmental changes in cortical and subcortical morphology from ages 14 to 19

ROIs	age 19 vs. age 14		
	t	Cohen's *d*	P_{FDR}
Cortical thickness			
Right_rostralmiddlefrontal	**-15.82**	**-1.04**	**0**
Left_inferiorparietal	**-15.43**	**-1.01**	**0**
Left_superiorparietal	**-14.37**	**-0.95**	**0**
Right_superiorfrontal	**-13.97**	**-0.92**	**0**
Right_inferiorparietal	**-13.85**	**-0.91**	**0**
Right_superiorparietal	**-13.46**	**-0.89**	**0**
Left_rostralmiddlefrontal	**-13.45**	**-0.88**	**0**
Right_precuneus	**-13.02**	**-0.86**	**0**
Left_supramarginal	**-12.88**	**-0.85**	**0**
Left_precuneus	**-12.6**	**-0.83**	**0**
Left_superiorfrontal	**-11.71**	**-0.77**	**0**
Right_parstriangularis	**-11.3**	**-0.74**	**0**
Right_supramarginal	**-11.13**	**-0.73**	**0**
Left_lateraloccipital	**-11.11**	**-0.73**	**0**
Right_posteriorcingulate	**-11.08**	**-0.73**	**0**
Right_postcentral	**-10.81**	**-0.71**	**0**
Left_postcentral	**-10.48**	**-0.69**	**0**
Right_paracentral	**-10.22**	**-0.67**	**0**
Left_cuneus	**-10.03**	**-0.66**	**0**
Right_medialorbitofrontal	**-9.9**	**-0.65**	**0**
Right_lateraloccipital	**-9.32**	**-0.61**	**0**
Right_cuneus	**-9.02**	**-0.59**	**0**
Right_caudalmiddlefrontal	**-8.98**	**-0.59**	**0**
Left_paracentral	**-8.87**	**-0.58**	**0**
Left_medialorbitofrontal	**-8.85**	**-0.58**	**0**
Left_lingual	**-8.84**	**-0.58**	**0**
Right_parsopercularis	**-8.84**	**-0.58**	**0**
Right_lingual	**-8.68**	**-0.57**	**0**
Left_posteriorcingulate	**-8.52**	**-0.56**	**0**
Left_frontalpole	**-8.05**	**-0.53**	**0**
Left_parstriangularis	**-7.97**	**-0.52**	**0**
Right_lateralorbitofrontal	**-7.96**	**-0.52**	**0**
Right_frontalpole	**-7.86**	**-0.52**	**0**

Left_parsopercularis	**-7.81**	**-0.51**	**0**
Left_bankssts	**-7.62**	**-0.5**	**0**
Right_parsorbitalis	**-7.4**	**-0.49**	**0**
Left_caudalmiddlefrontal	**-7.25**	**-0.48**	**0**
Right_isthmuscingulate	**-6.89**	**-0.45**	**0**
Left_pericalcarine	**-6.8**	**-0.45**	**0**
Left_isthmuscingulate	**-6.69**	**-0.44**	**0**
Left_lateralorbitofrontal	**-6.54**	**-0.43**	**0**
Right_bankssts	**-6.38**	**-0.42**	**0**
Right_pericalcarine	**-5.81**	**-0.38**	**0**
Right_caudalanteriorcingulate	**-5.58**	**-0.37**	**0**
Right_transversetemporal	**-5.35**	**-0.35**	**0**
Left_transversetemporal	**-5.15**	**-0.34**	**0**
Left_parsorbitalis	**-5.01**	**-0.33**	**0**
Left_insula	**-4.91**	**-0.32**	**0**
Right_precentral	**-4.24**	**-0.28**	**0**
Right_rostralanteriorcingulate	**-4.03**	**-0.26**	**0.0001**
Right_superiortemporal	**-3.89**	**-0.26**	**0.0001**
Left_caudalanteriorcingulate	**-3.73**	**-0.25**	**0.0002**
Left_precentral	**-3.7**	**-0.24**	**0.0003**
Right_insula	**-3.52**	**-0.23**	**0.0005**
Left_rostralanteriorcingulate	**-3.27**	**-0.21**	**0.0013**
Right_middletemporal	**-2.96**	**-0.19**	**0.0035**
Left_superiortemporal	**-2.18**	**-0.14**	**0.0327**
Left_fusiform	-0.9	-0.06	0.3848
Right_fusiform	-0.1	-0.01	0.917
Right_inferiortemporal	0.41	0.03	0.6912
Left_middletemporal	0.66	0.04	0.5254
Left_parahippocampal	0.99	0.07	0.3423
Right_parahippocampal	1.54	0.1	0.1342
Left_inferiortemporal	**3.47**	**0.23**	**0.0006**
Right_temporalpole	**5.35**	**0.35**	**0**
Right_entorhinal	**5.52**	**0.36**	**0**
Left_temporalpole	**5.65**	**0.37**	**0**
Left_entorhinal	**5.99**	**0.39**	**0**
Subcortical volumes			
Right_Caudate	**-5.36**	**-0.35**	**0**
Right_Putamen	**-4.65**	**-0.31**	**0**
Left_Caudate	**-4.29**	**-0.28**	**0.0001**
Left_Putamen	**-3.9**	**-0.26**	**0.0004**
Left_Thalamus_Proper	**-3.83**	**-0.25**	**0.0004**
Left_Accumbens_area	**-3.15**	**-0.21**	**0.0039**

Right_Thalamus_Proper	**-2.44**	**-0.16**	**0.0281**
Right_Accumbens_area	-1.81	-0.12	0.0875
Right_Pallidum	-0.58	-0.04	0.6457
Right_Hippocampus	-0.43	-0.03	0.6691
Left_Hippocampus	0.43	0.03	0.6691
Left_Pallidum	1.81	0.12	0.0875
Right_Amygdala	**1.93**	**0.13**	**0.0787**
Right_Lateral_Ventricle	**2.34**	**0.15**	**0.0313**
Left_Lateral_Ventricle	**2.42**	**0.16**	**0.0281**
Left_Amygdala	**3.17**	**0.21**	**0.0039**

*A t-value greater than 0 indicates an increase from age 14 to 19, while a t-value less than 0 indicates a decrease

Table S7. CCA results: DNAm clusters contributions to the first component

DNAm clusters	r	P_{FDR}
CCA: ΔDNAm–ΔCT		
**C12**	**-0.51**	**1.82E-30**
**C5**	**-0.18**	**1.57E-04**
C4	-0.07	0.1842
C1	-0.04	0.4500
C8	-0.01	0.8583
**C11**	**0.12**	**1.21E-02**
**C9**	**0.23**	**7.53E-07**
**C7**	**0.24**	**2.98E-07**
**C3**	**0.27**	**1.15E-08**
**C6**	**0.44**	**1.29E-22**
CCA: ΔDNAm–ΔSubcortical Volumes		
**C7**	**-0.36**	**4.00E-14**
**C6**	**-0.14**	**5.86E-03**
C5	-0.09	0.1050
C3	-0.04	0.3939
C9	0.05	0.2856
C4	0.05	0.2856
**C8**	**0.19**	**1.64E-04**
**C11**	**0.23**	**2.32E-06**
**C12**	**0.40**	**3.29E-18**
**C1**	**0.44**	**2.53E-21**

Table S8. CCA results: Brain regions contributions to the first component

Brain regions	r	P_{FDR}
CCA: ΔDNAm–ΔCT		
**Left_entorhinal**	**-0.23**	**2.92E-05**
**Right_frontalpole**	**-0.16**	**3.56E-03**
**Left_bankssts**	**-0.15**	**6.48E-03**
**Right_inferiorparietal**	**-0.15**	**7.52E-03**
**Right_isthmuscingulate**	**-0.14**	**1.49E-02**
**Left_lingual**	**-0.13**	**2.27E-02**
**Left_transversetemporal**	**-0.13**	**2.27E-02**
Right_temporalpole	-0.10	0.0844
Right_entorhinal	-0.09	0.1093
Left_temporalpole	-0.09	0.1093
Right_paracentral	-0.09	0.1137
Left_paracentral	-0.07	0.2020
Left_cuneus	-0.07	0.2089
Left_parahippocampal	-0.07	0.2100
Right_caudalanteriorcingulate	-0.06	0.2594
Right_superiorfrontal	-0.06	0.2900
Left_supramarginal	-0.05	0.3964
Right_middletemporal	-0.05	0.3964
Left_middletemporal	-0.05	0.4442
Left_inferiorparietal	-0.04	0.5187
Right_caudalmiddlefrontal	-0.04	0.5187
Left_fusiform	-0.04	0.5344
Right_rostralanteriorcingulate	-0.04	0.5344
Right_transversetemporal	-0.03	0.5895
Right_supramarginal	-0.03	0.5895
Right_precuneus	-0.03	0.5985
Left_precuneus	-0.02	0.7339
Right_superiorparietal	-0.02	0.7509
Left_pericalcarine	-0.01	0.8400
Right_parsorbitalis	-0.01	0.8698
Left_isthmuscingulate	-0.01	0.8698
Right_precentral	0.00	0.9845
Right_bankssts	0.00	0.9694
Left_frontalpole	0.01	0.9029
Left_inferiortemporal	0.02	0.7843
Right_insula	0.03	0.6276
Left_precentral	0.04	0.5187
Left_postcentral	0.05	0.4442

Left_superiorparietal	0.05	0.4440
Left_insula	0.07	0.2345
Right_parahippocampal	0.07	0.2182
Left_superiortemporal	0.07	0.2100
Right_inferiortemporal	0.07	0.2089
Left_caudalanteriorcingulate	0.08	0.1701
Right_rostralmiddlefrontal	0.08	0.1545
Right_cuneus	0.09	0.1093
Right_pericalcarine	0.09	0.1093
Left_lateraloccipital	0.09	0.1093
Right_fusiform	0.09	0.1093
Right_parsopercularis	0.09	0.1049
Right_lateraloccipital	0.10	0.0948
Right_superiortemporal	0.10	0.0948
Right_parstriangularis	0.11	0.0640
Left_superiorfrontal	0.11	0.0639
Right_posteriorcingulate	0.11	0.0506
**Right_postcentral**	**0.11**	**4.69E-02**
**Left_caudalmiddlefrontal**	**0.12**	**2.61E-02**
**Right_lingual**	**0.14**	**1.37E-02**
**Left_parsorbitalis**	**0.15**	**6.48E-03**
**Left_posteriorcingulate**	**0.17**	**2.45E-03**
**Left_rostralanteriorcingulate**	**0.17**	**2.45E-03**
**Right_medialorbitofrontal**	**0.18**	**6.33E-04**
**Left_rostralmiddlefrontal**	**0.18**	**6.33E-04**
**Right_lateralorbitofrontal**	**0.20**	**2.51E-04**
**Left_medialorbitofrontal**	**0.20**	**2.51E-04**
**Left_parsopercularis**	**0.20**	**2.51E-04**
**Left_parstriangularis**	**0.22**	**4.67E-05**
**Left_lateralorbitofrontal**	**0.23**	**2.92E-05**
CCA: ΔDNAm–ΔSubcortical Volumes		
**Right_Amygdala**	**-0.40**	**2.11E-17**
**Left_Amygdala**	**-0.31**	**1.58E-10**
**Left_Accumbens_area**	**-0.27**	**1.38E-08**
**Right_Thalamus_Proper**	**-0.20**	**2.51E-05**
**Right_Hippocampus**	**-0.13**	**9.70E-03**
**Right_Putamen**	**-0.12**	**1.72E-02**
Left_Hippocampus	-0.09	0.0621
Left_Pallidum	-0.03	0.6198
Left_Putamen	-0.01	0.9365
Left_Thalamus_Proper	0.00	0.9992

Right_Pallidum	0.08	0.1348
**Right_Accumbens_area**	**0.21**	**2.26E-05**
**Left_Lateral_Ventricle**	**0.27**	**1.38E-08**
**Right_Caudate**	**0.30**	**4.36E-10**
**Left_Caudate**	**0.38**	**1.10E-15**
**Right_Lateral_Ventricle**	**0.52**	**4.27E-31**

Table S9. Longitudinal associations between mental health, DNAm and MRI

	ΔCT CCA (first component)						ΔSubCortVol CCA (first component)					
	ΔDNAm			ΔCT			ΔDNAm			ΔSubCortVol		
	r	*P*	P_{FDR}	*r*	*P*	P_{FDR}	*r*	*P*	P_{FDR}	*r*	*P*	P_{FDR}
Changes in behaviours or symptoms (age 19 - age 14)												
Cannabis use	**-0.16**	**0.0007**	**0.0063**	**-0.15**	**0.0013**	**0.0063**	**0.11**	**0.0197**	**0.0494**	0.09	0.0765	0.3824
Binge drinking	**-0.16**	**0.0029**	**0.0076**	**-0.13**	**0.0177**	**0.0294**	0.10	0.0743	0.1238	0.03	0.6415	0.7402
Alcohol use	-0.09	0.0490	0.0700	0.00	0.9952	0.9952	0.05	0.3519	0.3519	0.07	0.1638	0.4096
Smoking (tobacco)	-0.09	0.0702	0.0877	-0.04	0.3665	0.4072	0.07	0.1508	0.1885	-0.02	0.7402	0.7402
Depressive symptoms	**-0.12**	**0.0143**	**0.0286**	**-0.14**	**0.0030**	**0.0076**	**0.12**	**0.0192**	**0.0494**	0.04	0.3864	0.6439
Changes in behaviours or symptoms (age 19 - age 14), controlled SES												
Cannabis use	**-0.16**	**0.0008**		**-0.15**	**0.0014**		**0.11**	**0.0217**		0.08	0.0866	
Binge drinking	**-0.16**	**0.0030**		**-0.13**	**0.0179**		0.10	0.0765		0.02	0.6643	
Alcohol use	-0.09	0.0717		0.00	0.9283		0.04	0.4185		0.06	0.2370	
Smoking (tobacco)	-0.08	0.0809		-0.04	0.3827		0.07	0.1661		-0.02	0.6701	
Depressive symptoms	**-0.11**	**0.0204**		**-0.14**	**0.0037**		**0.11**	**0.0238**		0.04	0.4667	

Table S10. Associations between DNAm and psychopathology

DNAm cluster	Associations with substance use																Associations with mental health symptoms					
	Age 14								Age 19								Age 14		Age 19			
	Alcohol use		Binge drinking		Smoking		Cannabis use		Alcohol use		Binge drinking		Smoking		Cannabis use		Depressive symptoms		Depressive symptoms		Negetive Psychotis symptoms	
	r	P_{FDR}	*r*	P_{FDR}	*r*	P_{FDR}	*r*	P_{FDR}	*r*	P_{FDR}	*r*	P_{FDR}	*r*	P_{FDR}	*r*	P_{FDR}	*r*	P_{FDR}	*r*	P_{FDR}	*r*	P_{FDR}
**C1**	-0.06	0.5307	-0.12	0.0595	-0.10	0.1752	-0.09	0.1752	-0.02	0.6868	0.03	0.6047	0.08	0.2147	0.03	0.6304	0.02	0.7138	**0.13**	**0.020**	0.10	0.142
C3	0.02	0.8317	0.01	0.9104	-0.05	0.6235	-0.01	0.8767	-0.07	0.2252	-0.07	0.2385	-0.05	0.3979	-0.09	0.1042	-0.08	0.2473	0.01	0.906	-0.01	0.920
**C4**	0.06	0.5175	**0.15**	**0.0175**	0.09	0.1752	0.07	0.3390	0.03	0.637	-0.01	0.8409	0.02	0.7514	-0.01	0.8062	0.00	0.9209	0.05	0.468	0.06	0.359
**C5**	-0.03	0.6816	-0.02	0.7140	-0.07	0.3390	0.05	0.6235	0.05	0.4021	0.05	0.4445	0.06	0.2904	**0.12**	**0.0324**	-0.03	0.5593	0.06	0.453	0.02	0.896
**C6**	0.01	0.9098	-0.01	0.8767	-0.07	0.3390	-0.07	0.3390	-0.07	0.2252	**-0.13**	**0.0202**	**-0.16**	**0.0032**	**-0.2**	**0.0005**	-0.07	0.2473	-0.03	0.572	-0.04	0.606
**C7**	-0.03	0.7140	0.03	0.7140	0.00	0.9199	-0.05	0.6042	-0.02	0.7514	-0.05	0.4445	-0.07	0.2488	-0.03	0.637	**-0.13**	**0.0409**	**-0.16**	**0.008**	**-0.14**	**0.031**
C8	-0.04	0.6626	-0.04	0.6743	-0.10	0.1752	-0.02	0.7140	-0.1	0.0629	-0.07	0.2385	-0.04	0.4659	0.01	0.8062	-0.06	0.3131	0.06	0.453	0.09	0.195
**C9**	0.03	0.6816	**0.16**	**0.0112**	0.06	0.4481	-0.02	0.7140	**-0.14**	**0.0113**	**-0.12**	**0.0324**	**-0.14**	**0.0107**	**-0.13**	**0.0202**	-0.10	0.1354	-0.04	0.569	-0.01	0.920
**C11**	-0.03	0.6816	-0.09	0.2403	-0.13	0.0595	-0.10	0.1752	-0.1	0.0629	-0.09	0.1393	**-0.11**	**0.0418**	**-0.17**	**0.0031**	-0.06	0.3379	0.01	0.906	0.00	0.920
**C12**	-0.04	0.6816	-0.04	0.6423	0.03	0.6816	0.04	0.6743	0.06	0.2944	0.06	0.3037	**0.16**	**0.0031**	**0.17**	**0.0031**	-0.04	0.5582	0.10	0.086	0.08	0.195
Sensitivity analyses, controlling for smoking																						
	r	*P*	*r*	*P*	*r*	*P*	*r*	*P*	*r*	*P*	*r*	*P*	*r*	*P*	*r*	*P*	*r*	*P*	*r*	*P*	*r*	*P*
**C1**	-0.01	0.8777	-0.08	0.0873			-0.05	0.2833	-0.0658	0.152	6E-05	0.99891			-0.018	0.7005	0.02	0.5945	**0.13**	**0.005**	0.10	0.039
C3	0.04	0.3378	0.05	0.3152			0.02	0.7302	-0.0573	0.2124	-0.054	0.24256			-0.079	0.0842	-0.08	0.0990	0.01	0.823	-0.01	0.853
**C4**	0.01	0.7449	**0.12**	**0.0090**	NA		0.03	0.5080	0.0219	0.6329	-0.019	0.67625	NA		-0.029	0.5215	-0.01	0.8646	0.05	0.301	0.06	0.200
**C5**	0.00	0.9248	0.03	0.5460			0.10	0.0299	0.0248	0.5899	0.0212	0.64383			**0.103**	**0.0245**	-0.03	0.4503	0.05	0.288	0.02	0.651
**C6**	0.05	0.2963	0.04	0.3478			-0.04	0.3804	-0.0019	0.9668	-0.069	0.13374			**-0.131**	**0.0041**	-0.07	0.1113	-0.02	0.628	-0.03	0.523

**C7**	-0.03	0.5551	0.04	0.4072			-0.06	0.2229	0.014	0.7601	-0.018	0.69634			0.014	0.7557	**-0.13**	**0.0044**	**-0.16**	**0.001**	**-0.13**	**0.006**
C8	0.00	0.9193	0.03	0.5739			0.03	0.5287	-0.096	0.0363	-0.057	0.2106			0.046	0.3165	-0.06	0.1643	0.06	0.210	0.09	0.060
**C9**	0.00	0.9351	**0.16**	**0.0004**			-0.06	0.1541	-0.0869	0.0582	-0.065	0.15849			-0.057	0.2132	-0.10	0.0252	-0.03	0.512	0.00	0.922
**C11**	0.03	0.5065	-0.01	0.8225			-0.04	0.3825	-0.0605	0.1876	-0.043	0.34813			**-0.126**	**0.0061**	-0.05	0.2360	0.01	0.760	0.01	0.899
**C12**	-0.06	0.2035	-0.08	0.0778			0.03	0.5742	-0.0132	0.7743	-0.012	0.79927			**0.092**	**0.0453**	-0.04	0.3622	0.09	0.047	0.07	0.139
Sensitivity analyses, controlling for cannabis use																						
	r	*P*	*r*	*P*	*r*	*P*	*r*	*P*	*r*	*P*	*r*	*P*	*r*	*P*	*r*	*P*	*r*	*P*	*r*	*P*	*r*	*P*
**C1**	-0.03	0.5316	-0.09	0.0370	-0.06	0.1599			-0.0384	0.403	0.0227	0.62078	0.07328	0.11032			0.02	0.6889	**0.13**	**0.004**	0.10	0.034
C3	0.02	0.6612	0.01	0.7945	-0.05	0.2837			-0.042	0.361	-0.035	0.44275	0.00382	0.93377			-0.08	0.0915	0.01	0.794	0.00	0.973
**C4**	0.04	0.4126	**0.13**	**0.0035**	0.07	0.1528			0.0356	0.4382	-0.004	0.92557	0.03252	0.47905			0.00	0.9529	0.05	0.279	0.06	0.176
C5	-0.05	0.2959	-0.05	0.2945	-0.11	0.0114			0.0054	0.9057	-0.003	0.94469	-0.0082	0.85756			-0.04	0.4380	0.05	0.307	0.01	0.789
C6	0.03	0.5072	0.02	0.6669	-0.04	0.3665	NA		0.0014	0.9753	-0.055	0.23138	-0.0571	0.21347	NA		-0.08	0.0916	-0.02	0.617	-0.02	0.672
**C7**	-0.01	0.7995	0.05	0.2575	0.02	0.5897			-0.008	0.8614	-0.037	0.41771	-0.0638	0.16441			**-0.13**	**0.0039**	**-0.16**	**0.001**	**-0.13**	**0.006**
C8	-0.04	0.4341	-0.03	0.4825	-0.10	0.0335			-0.1184	0.0097	-0.083	0.07182	-0.0617	0.17927			-0.07	0.1427	0.05	0.244	0.08	0.075
**C9**	0.04	0.3585	**0.19**	**2.55E-05**	0.09	0.0556			-0.1006	0.0281	-0.074	0.10852	-0.0851	0.06366			-0.10	0.0274	-0.03	0.469	0.01	0.855
C11	0.00	0.9287	-0.05	0.2702	-0.09	0.0544			-0.0455	0.3221	-0.021	0.6446	-0.0207	0.652			-0.06	0.1835	0.02	0.732	0.02	0.726
C12	-0.05	0.2755	-0.06	0.1555	0.01	0.7655			-0.002	0.9646	-0.01	0.82779	0.08246	0.07226			-0.04	0.3866	0.10	0.041	0.06	0.178
Sensitivity analyses, controlling for SES (age 14)																						
	r	*P*	*r*	*P*	*r*	*P*	*r*	*P*	*r*	*P*	*r*	*P*	*r*	*P*	*r*	*P*	*r*	*P*	*r*	*P*	*r*	*P*
**C1**	-0.06	0.2162	-0.12	0.0060	-0.10	0.0229	-0.09	0.0379	-0.0251	0.5852	0.033	0.47346	0.07723	0.09272	0.03	0.5078	0.02	0.6641	**0.14**	**0.004**	0.10	0.028
C3	0.01	0.7556	0.01	0.8422	-0.05	0.2710	-0.01	0.7830	-0.0661	0.1504	-0.068	0.13829	-0.0498	0.27919	-0.093	0.0436	-0.07	0.1191	0.01	0.894	-0.01	0.753
**C4**	0.06	0.2110	**0.15**	**0.0010**	0.10	0.0361	0.08	0.0972	0.0199	0.6647	-0.013	0.78355	0.01697	0.71227	-0.015	0.7497	-0.01	0.8053	0.05	0.299	0.06	0.181
**C5**	-0.03	0.5006	-0.02	0.5598	-0.07	0.1067	0.05	0.3018	0.0601	0.1911	0.0502	0.27511	0.06579	0.15224	**0.123**	**0.0074**	-0.04	0.4013	0.06	0.232	0.02	0.606
**C6**	0.01	0.8488	-0.01	0.8425	-0.07	0.1063	-0.07	0.1091	-0.0871	0.0579	**-0.138**	**0.00266**	**-0.165**	**0.0003**	**-0.202**	**9E-06**	-0.07	0.1359	-0.04	0.417	-0.04	0.401
**C7**	-0.02	0.5979	0.03	0.5596	0.00	0.9253	-0.05	0.2687	-0.0062	0.8931	-0.041	0.37082	-0.0657	0.15273	-0.026	0.577	**-0.13**	**0.0046**	**-0.16**	**0.001**	**-0.14**	**0.004**

C8	-0.04	0.3731	-0.04	0.3822	-0.09	0.0370	-0.02	0.5965	-0.1036	0.0239	-0.069	0.13283	-0.0426	0.35478	0.012	0.786	-0.07	0.1492	0.06	0.226	0.09	0.071
**C9**	0.03	0.4789	**0.16**	**0.0003**	0.06	0.1560	-0.02	0.6348	**-0.1366**	**0.0029**	**-0.118**	**0.01008**	**-0.1428**	**0.0018**	**-0.129**	**0.005**	-0.11	0.0165	-0.04	0.376	-0.01	0.866
**C11**	-0.04	0.4369	-0.09	0.0706	-0.13	0.0045	-0.10	0.0269	-0.1115	0.015	-0.092	0.04507	**-0.1156**	**0.01171**	**-0.168**	**0.0002**	-0.05	0.2684	0.00	0.944	0.00	0.990
**C12**	-0.03	0.4975	-0.04	0.3401	0.03	0.4950	0.04	0.4293	0.0671	0.1442	0.0649	0.15802	**0.16592**	**0.00028**	**0.169**	**0.0002**	-0.03	0.5418	0.11	0.020	0.08	0.083
Sensitivity analyses, controlling for pubertal development (age 14)																						
	r	*P*	*r*	*P*	*r*	*P*	*r*	*P*	*r*	*P*	*r*	*P*	*r*	*P*	*r*	*P*	*r*	*P*	*r*	*P*	*r*	*P*
**C1**	-0.05	0.2861	-0.12	0.0083	-0.10	0.0315	-0.09	0.0494	-0.0228	0.6195	0.0337	0.46356	0.08121	0.07702	0.032	0.4848	0.02	0.5945	**0.14**	**0.004**	0.10	0.029
C3	0.02	0.6954	0.01	0.8612	-0.05	0.3088	-0.01	0.8358	-0.0736	0.1089	-0.075	0.10148	-0.0477	0.29945	-0.093	0.0425	-0.08	0.0990	0.00	0.971	-0.02	0.695
**C4**	0.05	0.2754	**0.15**	**0.0014**	0.09	0.0560	0.07	0.1397	0.0332	0.4704	-0.011	0.81681	0.03169	0.49076	-0.007	0.8773	-0.01	0.8646	0.05	0.330	0.05	0.257
**C5**	-0.02	0.6445	-0.02	0.7271	-0.07	0.1489	0.06	0.2167	0.0479	0.2977	0.0454	0.32384	0.05825	0.20502	**0.118**	**0.0101**	-0.03	0.4503	0.06	0.240	0.03	0.575
**C6**	0.01	0.8055	-0.01	0.8566	-0.07	0.1277	-0.07	0.1310	-0.0662	0.1497	**-0.134**	**0.0034**	**-0.1437**	**0.00169**	**-0.192**	**3E-05**	-0.07	0.1113	-0.04	0.378	-0.05	0.260
**C7**	-0.03	0.4889	0.02	0.6196	-0.01	0.8351	-0.06	0.2264	-0.0156	0.7349	-0.045	0.32284	-0.0633	0.16844	-0.025	0.5829	**-0.13**	**0.0044**	**-0.16**	**0.001**	**-0.13**	**0.004**
C8	-0.03	0.5535	-0.03	0.5492	-0.08	0.0670	-0.01	0.7939	-0.1097	0.0168	-0.07	0.12501	-0.0537	0.24248	0.008	0.869	-0.06	0.1643	0.06	0.216	0.09	0.056
**C9**	0.03	0.5581	**0.16**	**0.0004**	0.06	0.2086	-0.03	0.5176	**-0.1404**	**0.0022**	**-0.124**	**0.00692**	**-0.1403**	**0.00218**	**-0.128**	**0.0052**	-0.10	0.0252	-0.05	0.328	-0.01	0.794
**C11**	-0.02	0.6262	-0.08	0.0901	-0.12	0.0103	-0.09	0.0490	-0.0997	0.0299	-0.09	0.04892	**-0.1007**	**0.02828**	**-0.162**	**0.0004**	-0.05	0.2360	0.00	0.975	-0.01	0.840
**C12**	-0.03	0.5707	-0.04	0.4403	0.04	0.3637	0.05	0.2947	0.056	0.223	0.0613	0.18211	**0.15184**	**0.0009**	**0.163**	**0.0004**	-0.04	0.3622	0.11	0.020	0.09	0.057

Part-2: Supplementary Figures

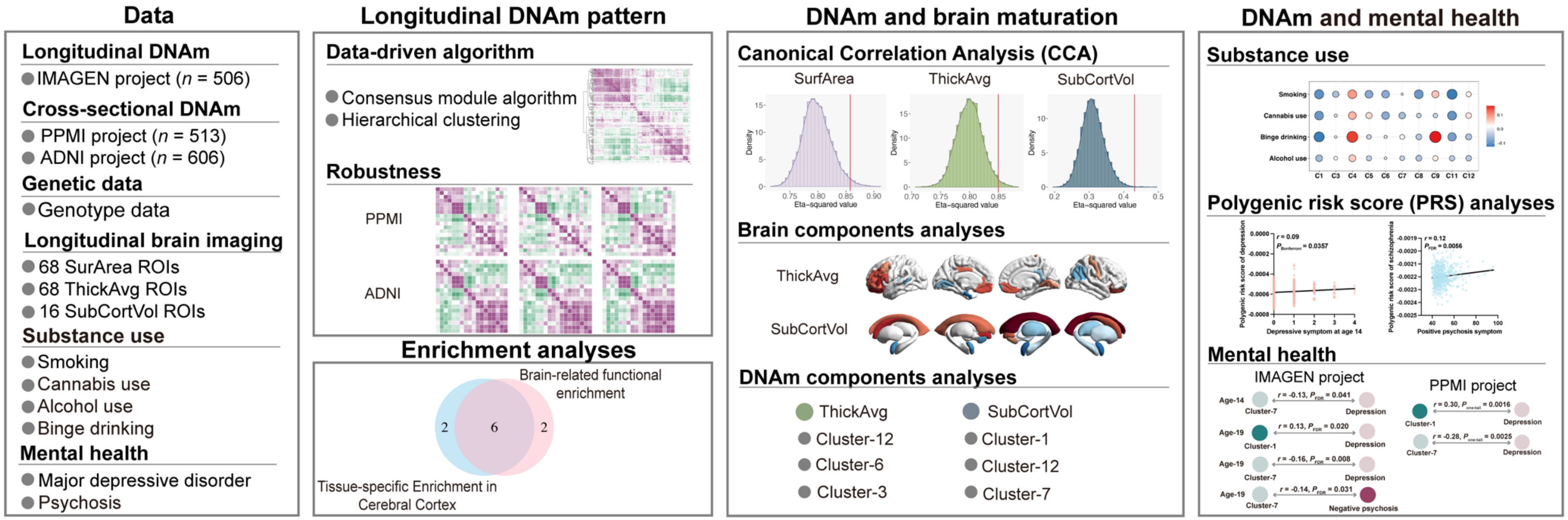

Figure S1. Schematic overview of the study.

This schematic diagram provides a visual representation of the framework for this study.

Cluster_1

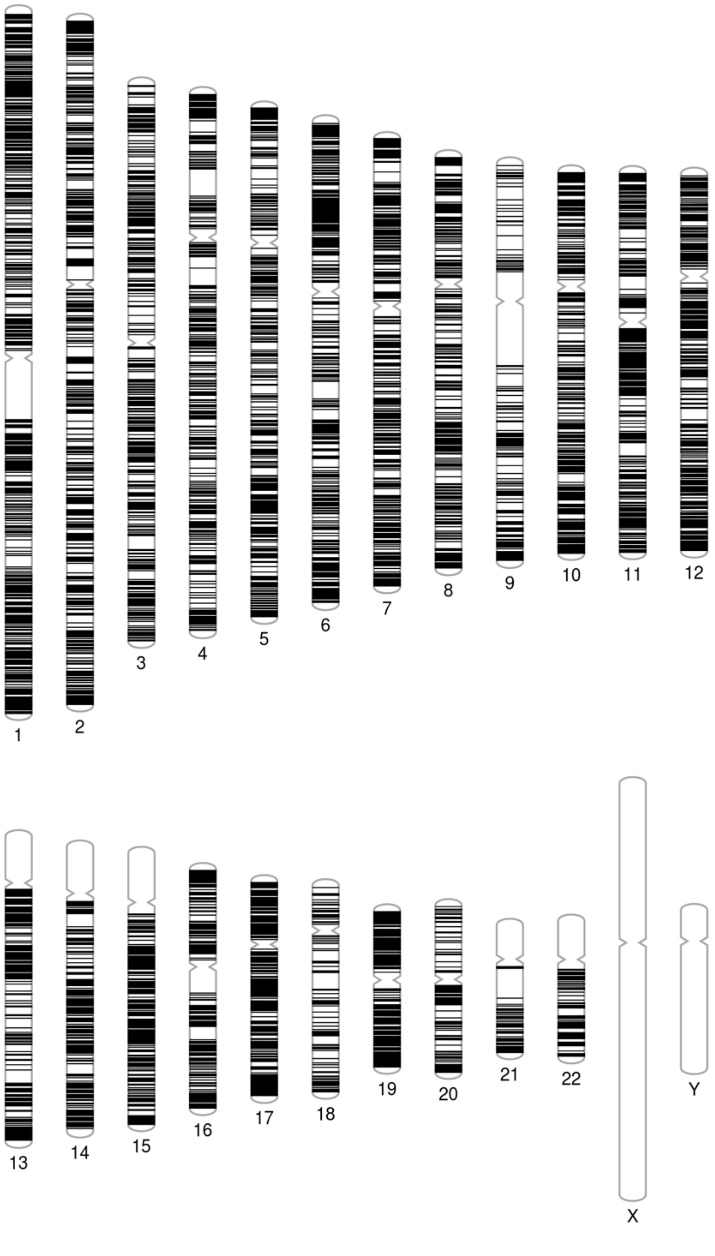

Cluster_2

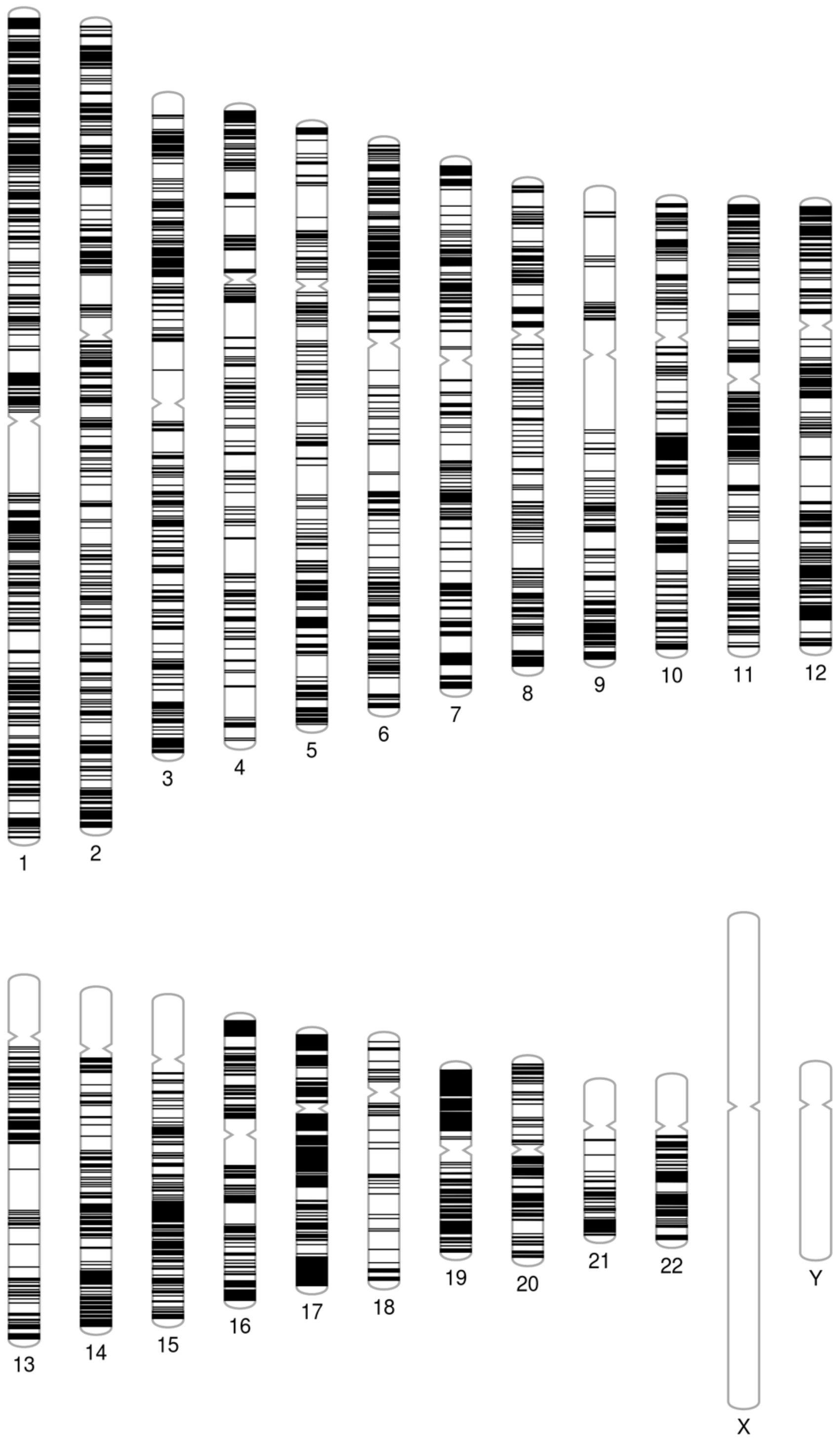

Cluster_3

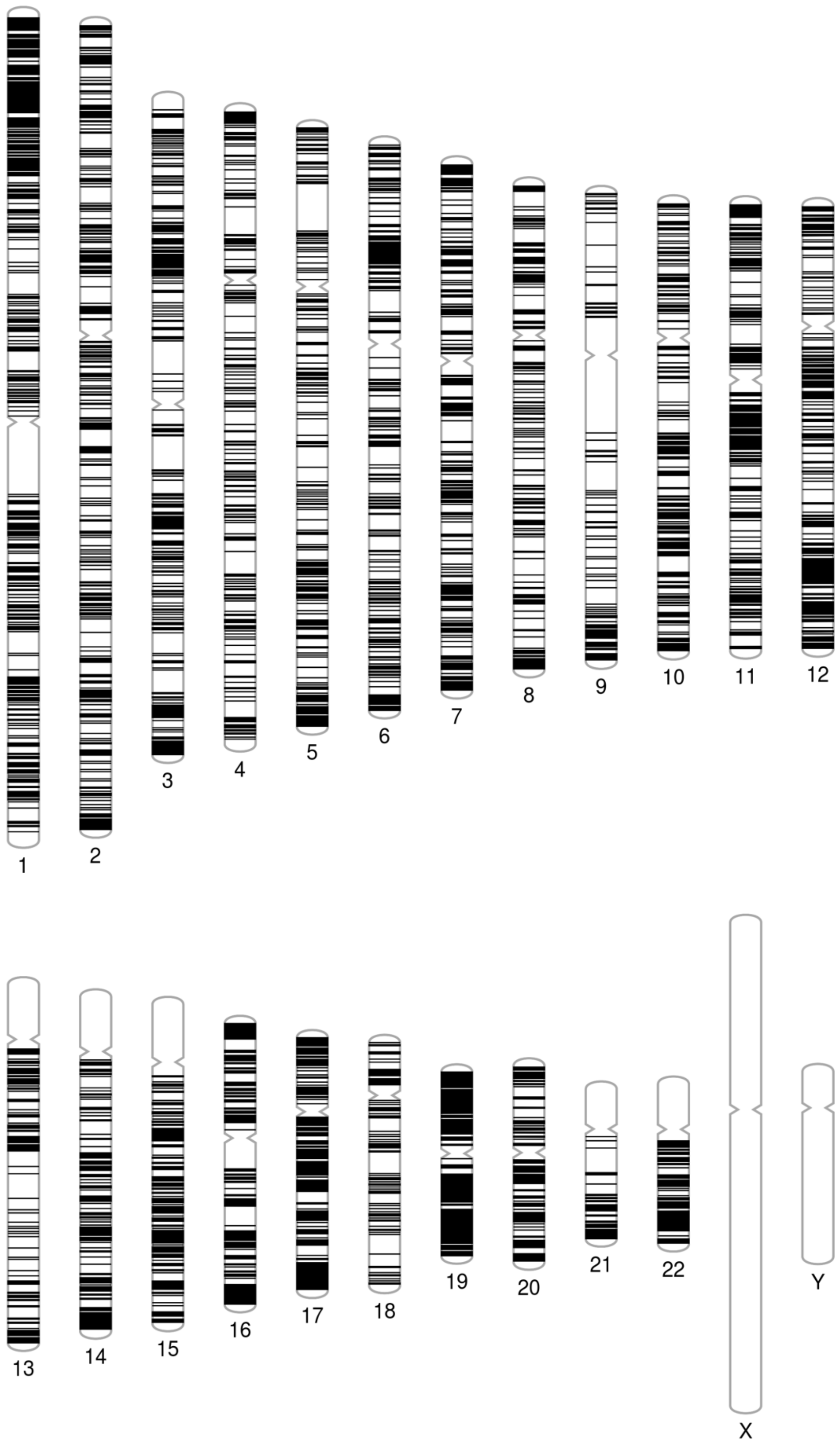

Cluster_4

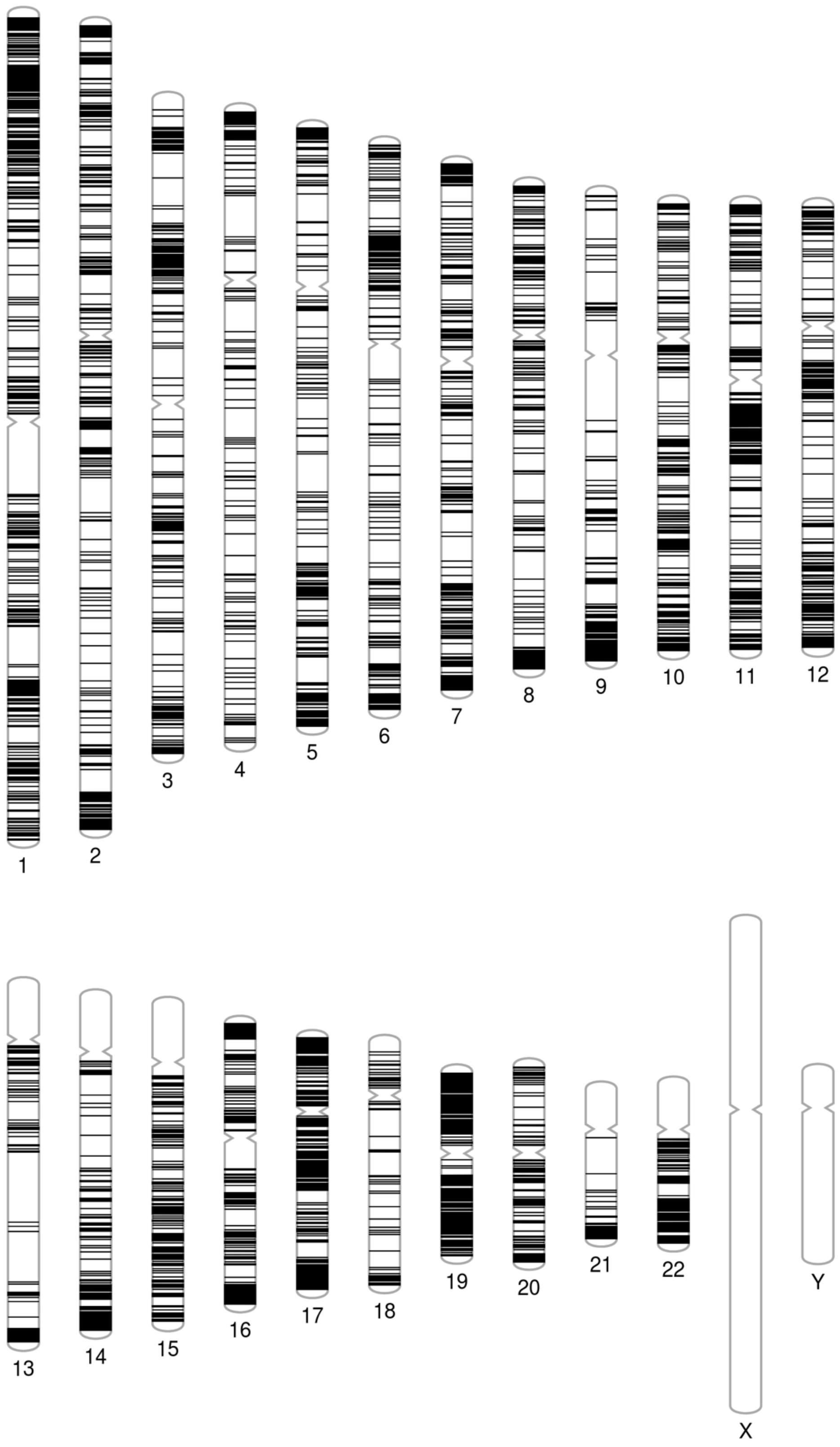

Cluster_5

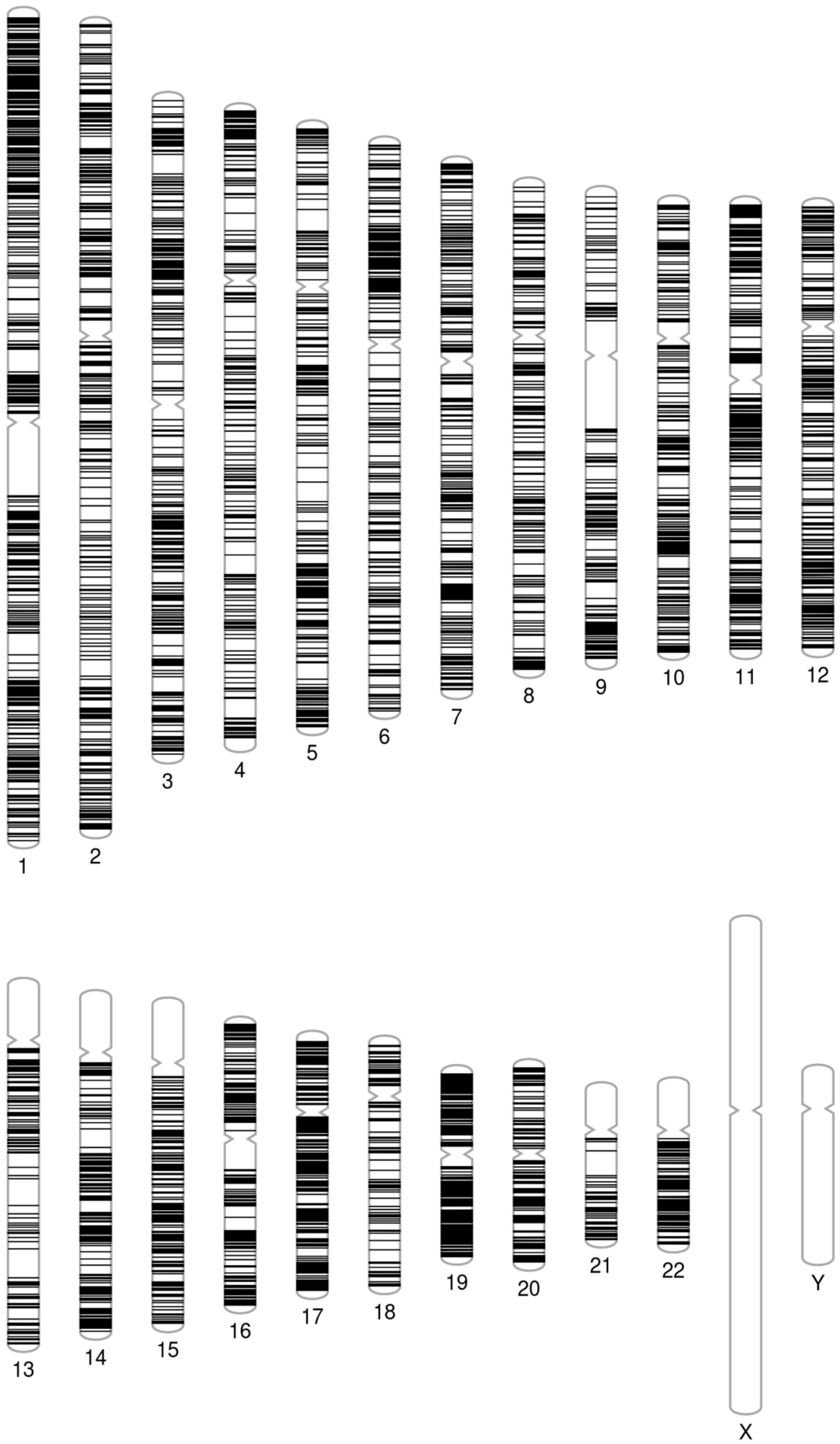

Cluster_6

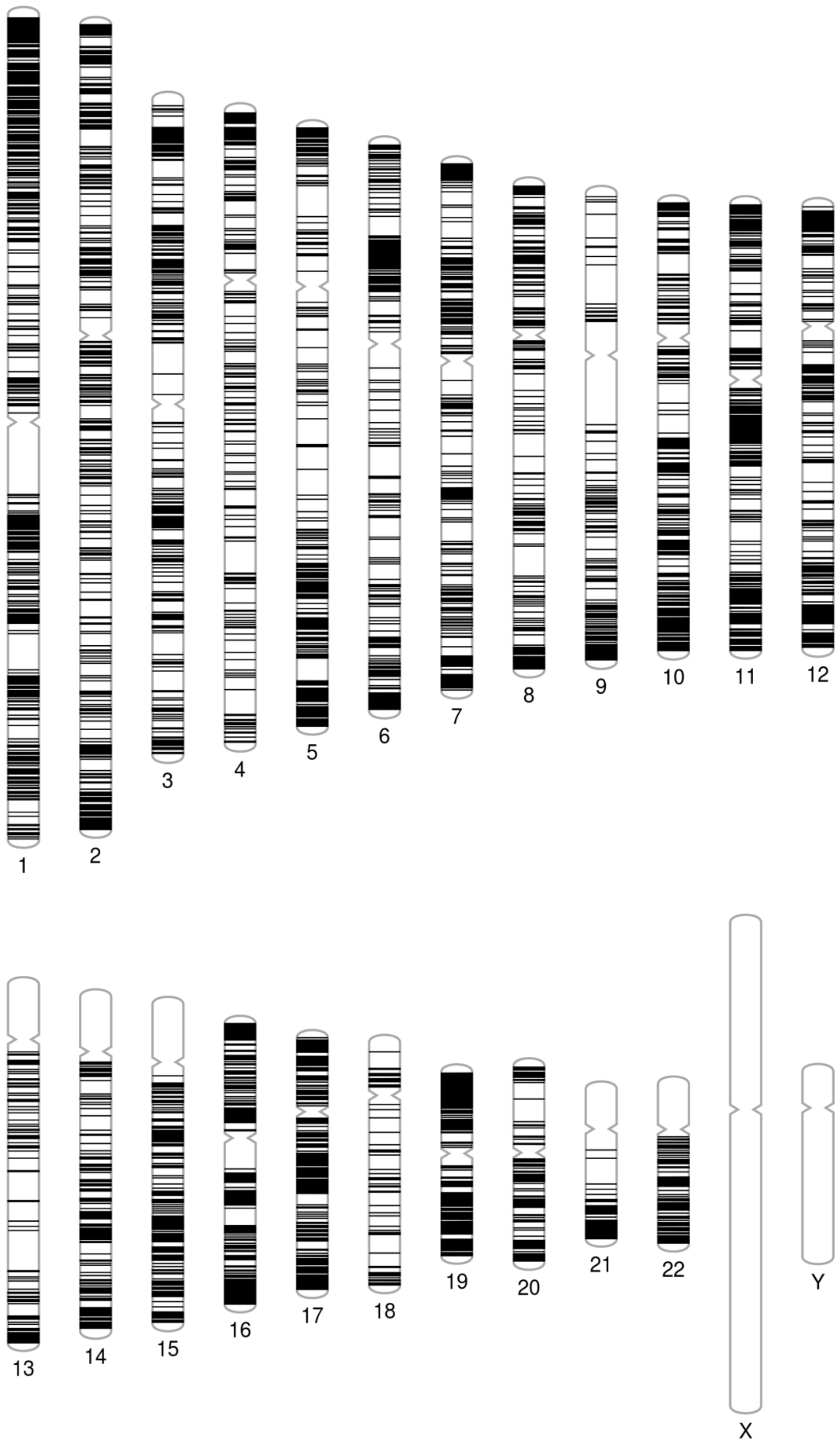

Cluster_7

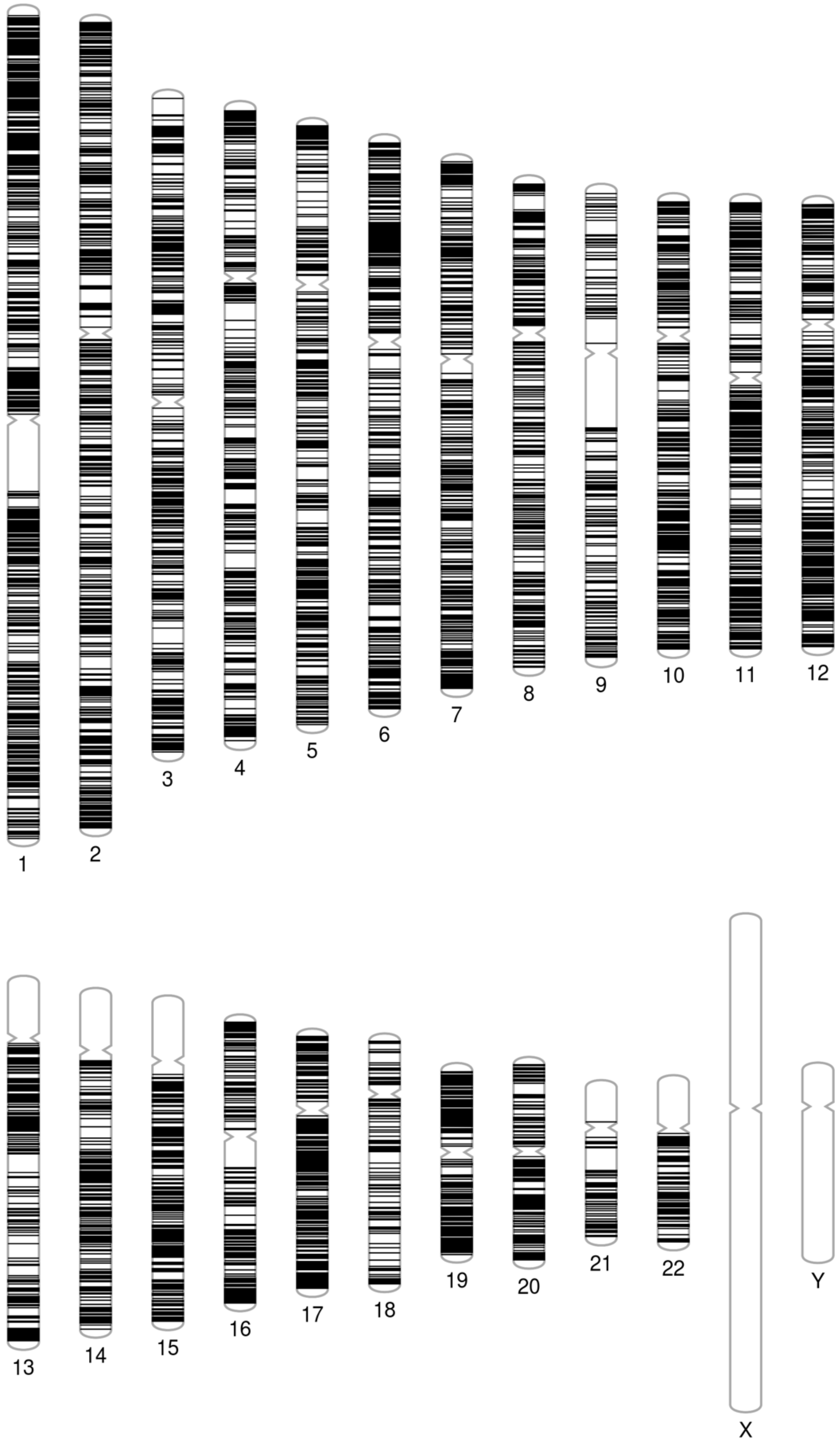

Cluster_8

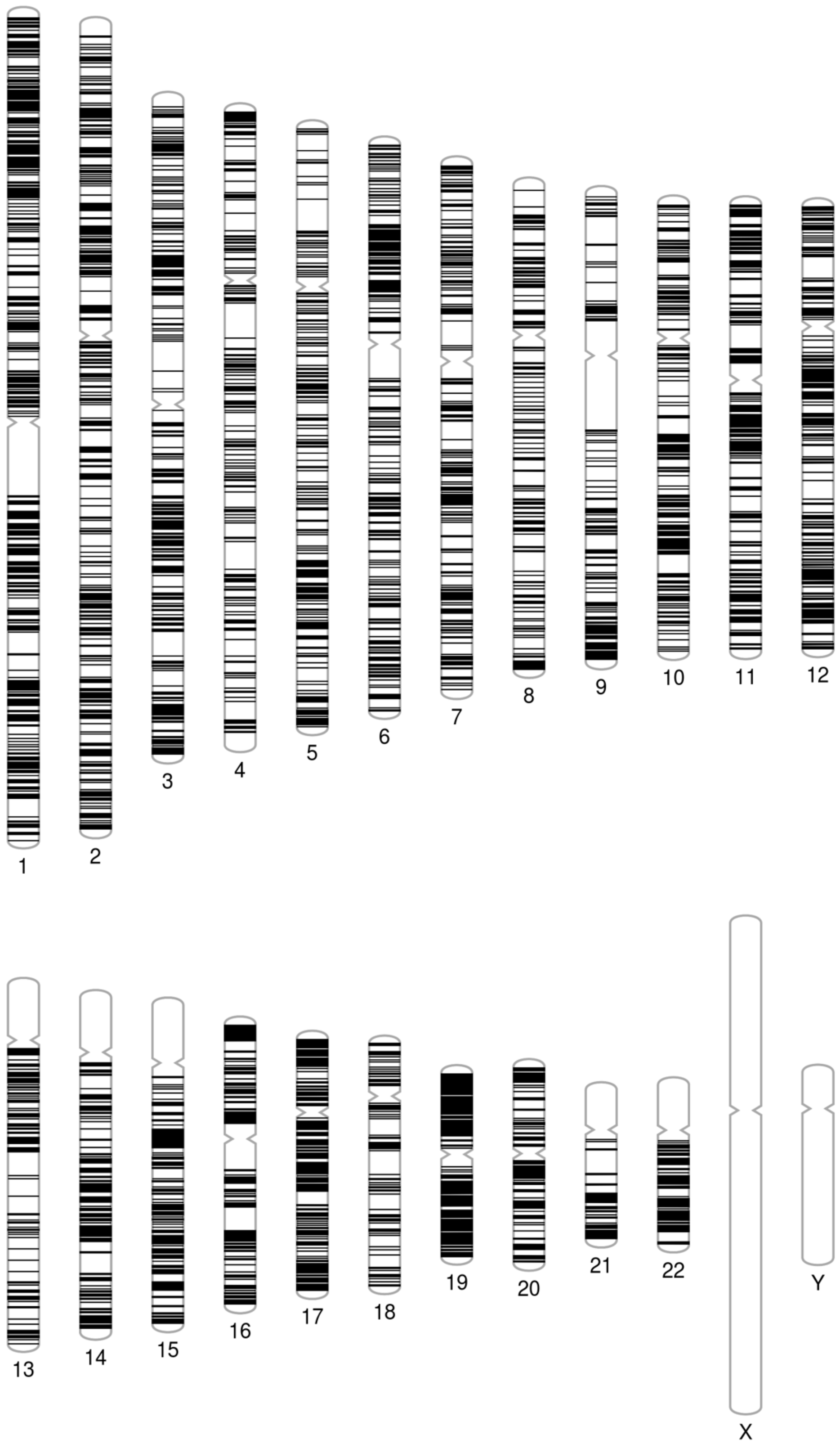

Cluster_9

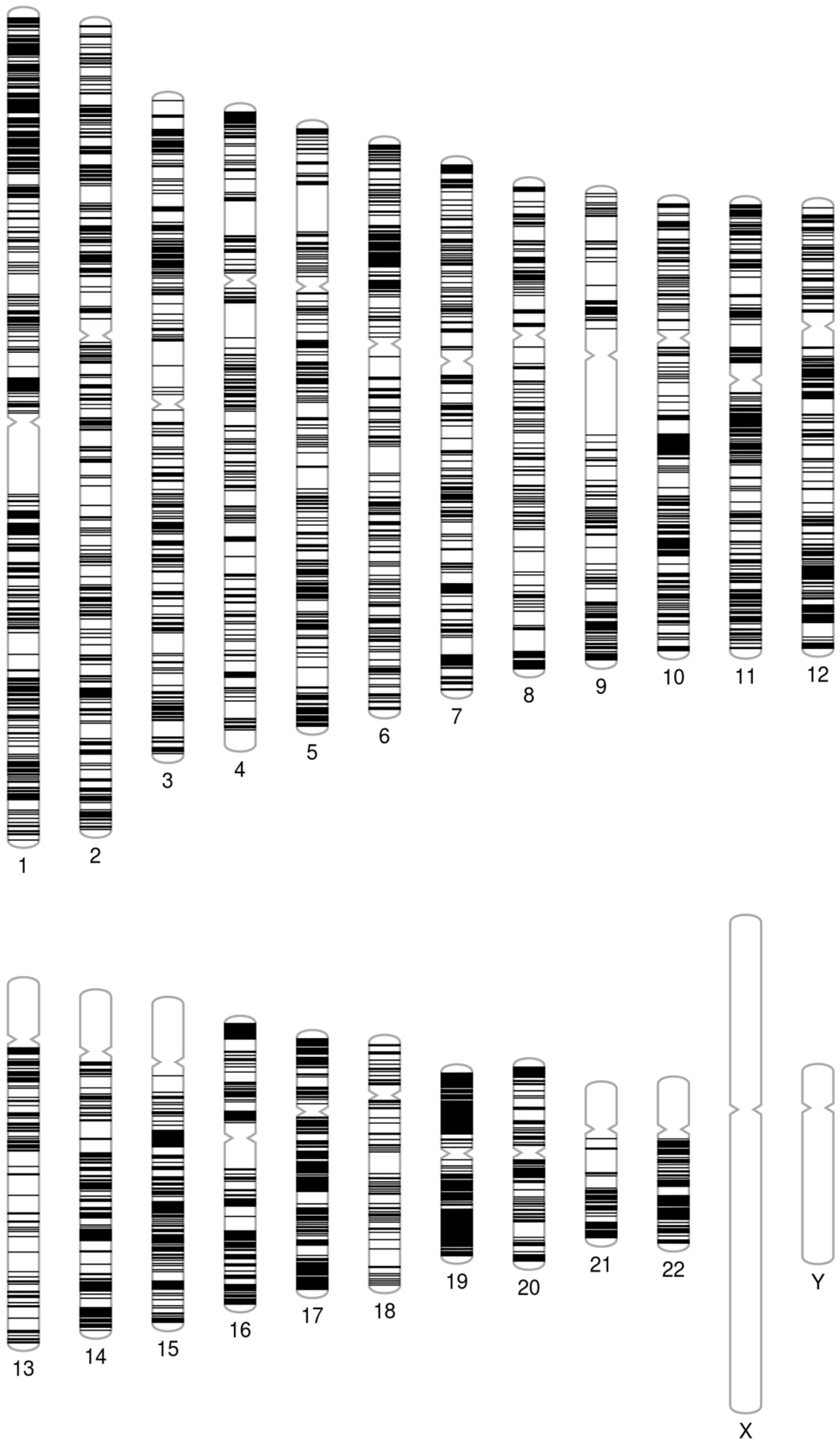

Cluster_10

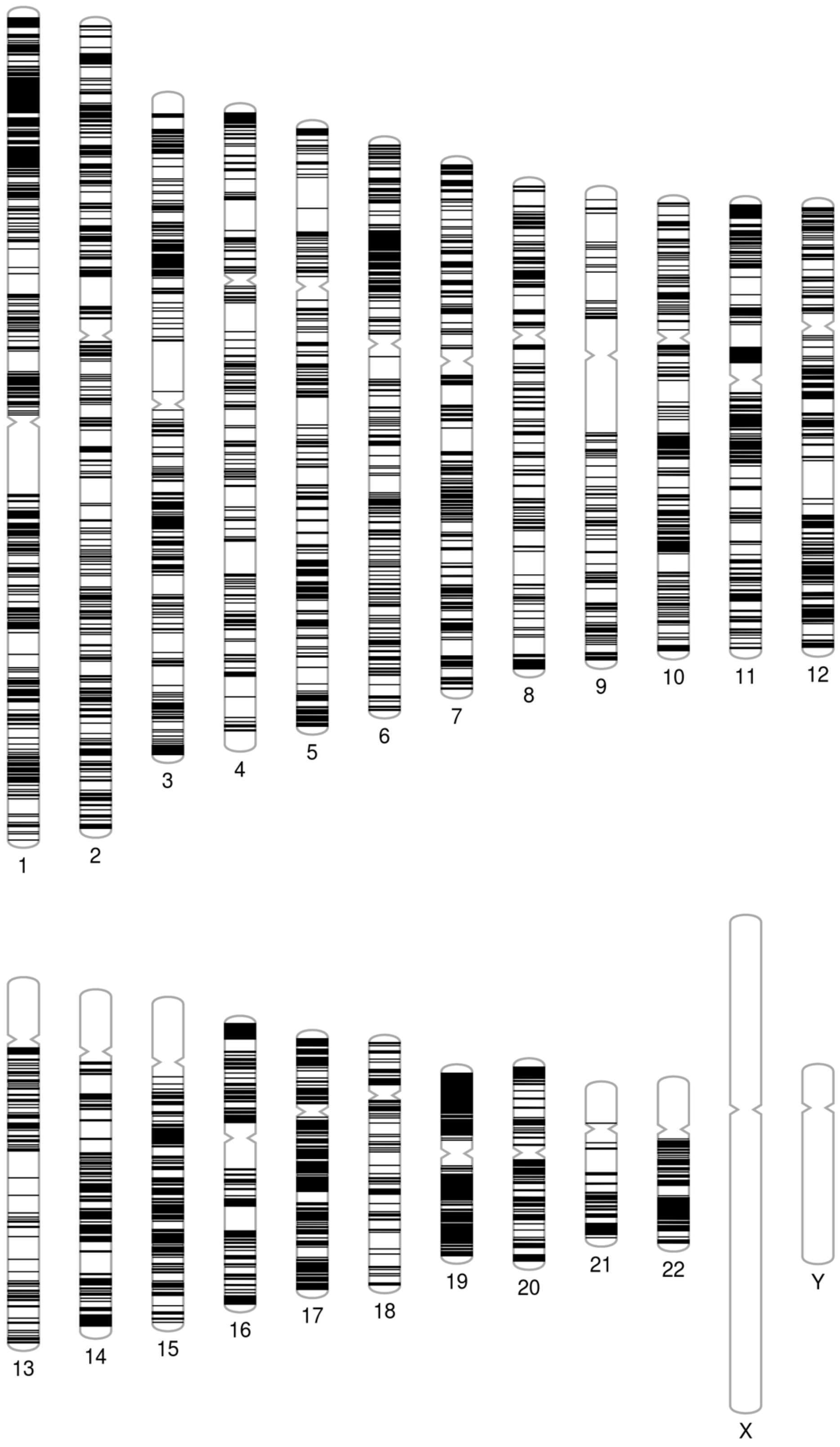

Cluster_11

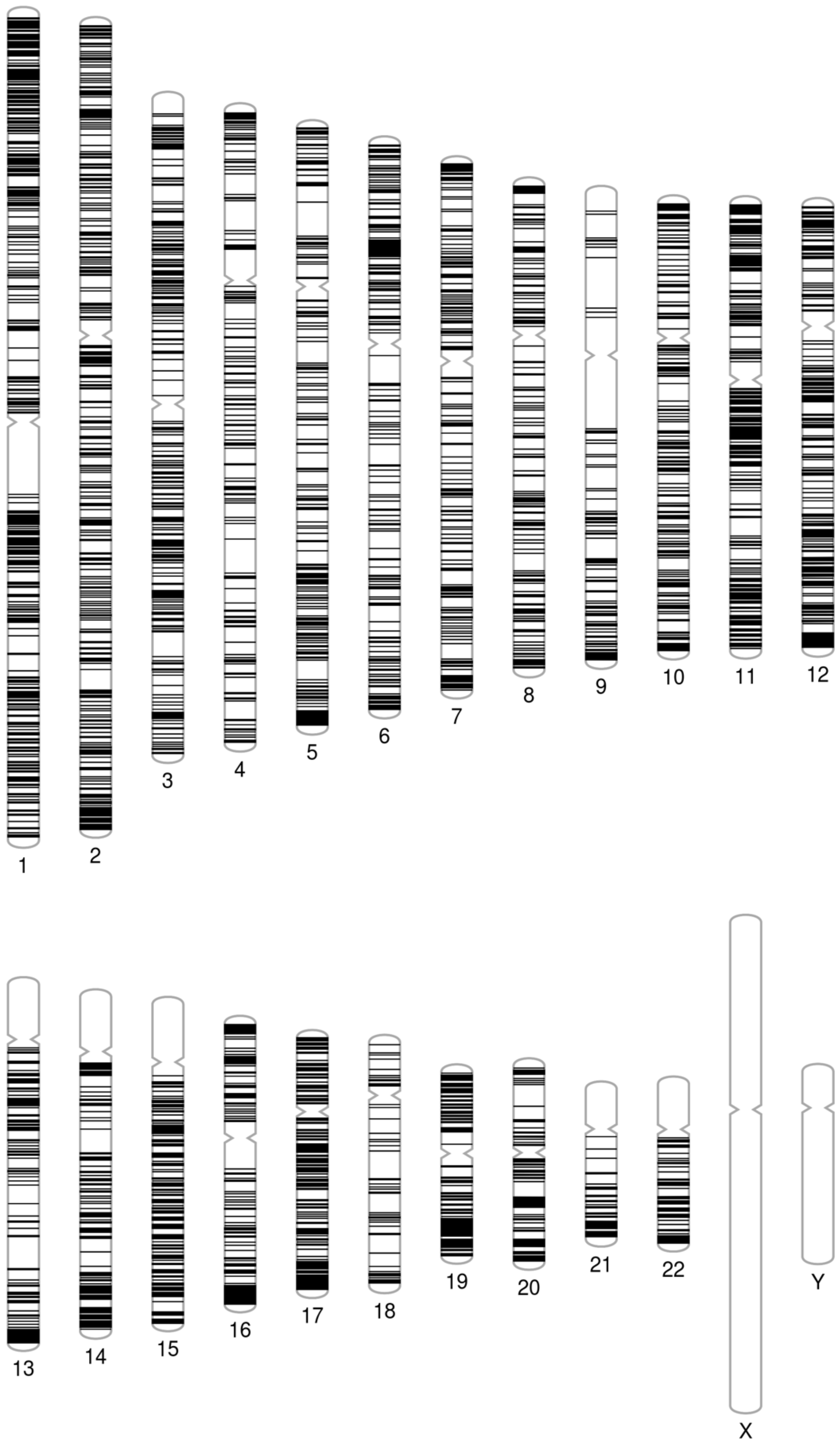

Cluster_12

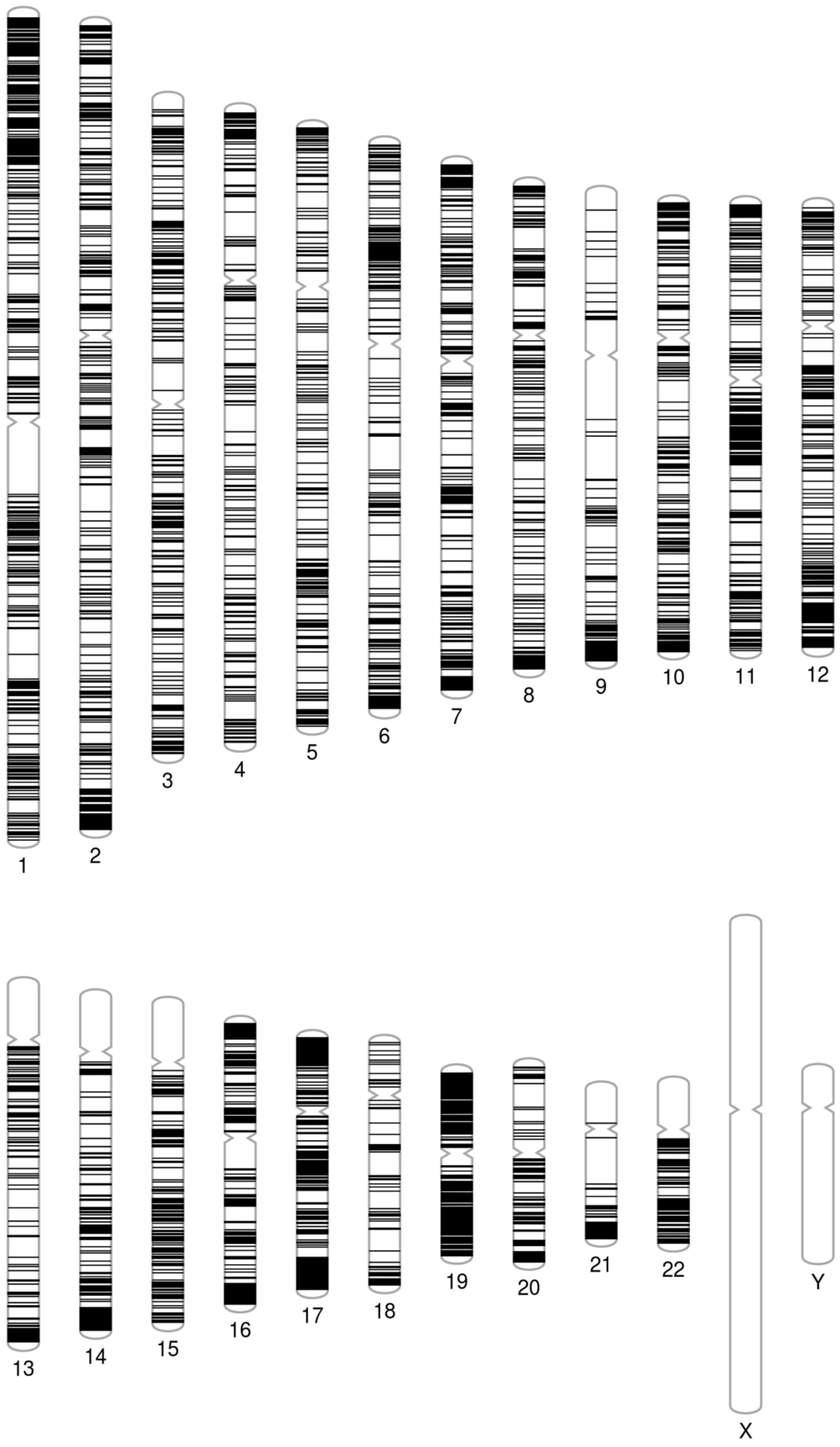

Cluster_13

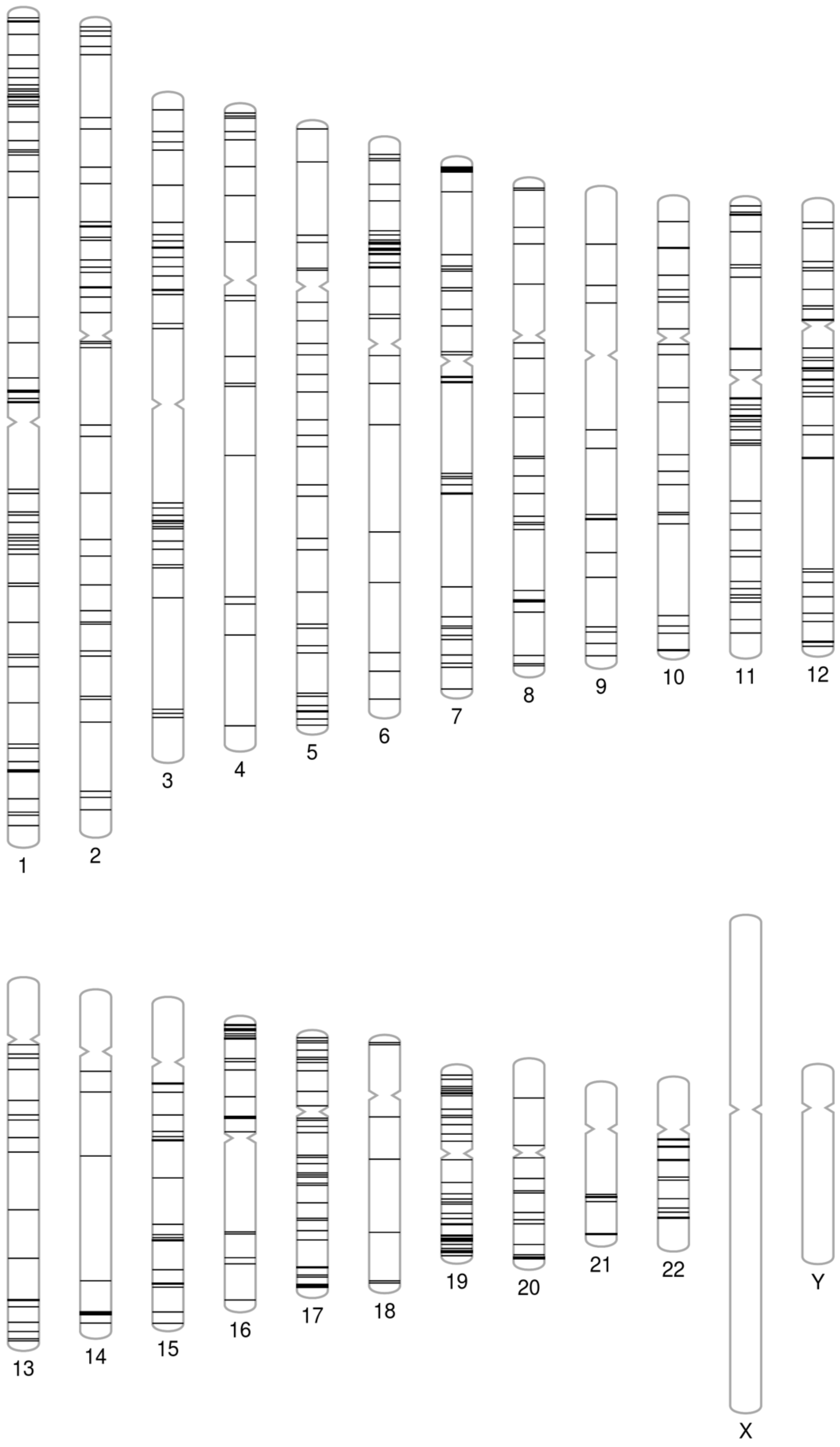

Cluster_14

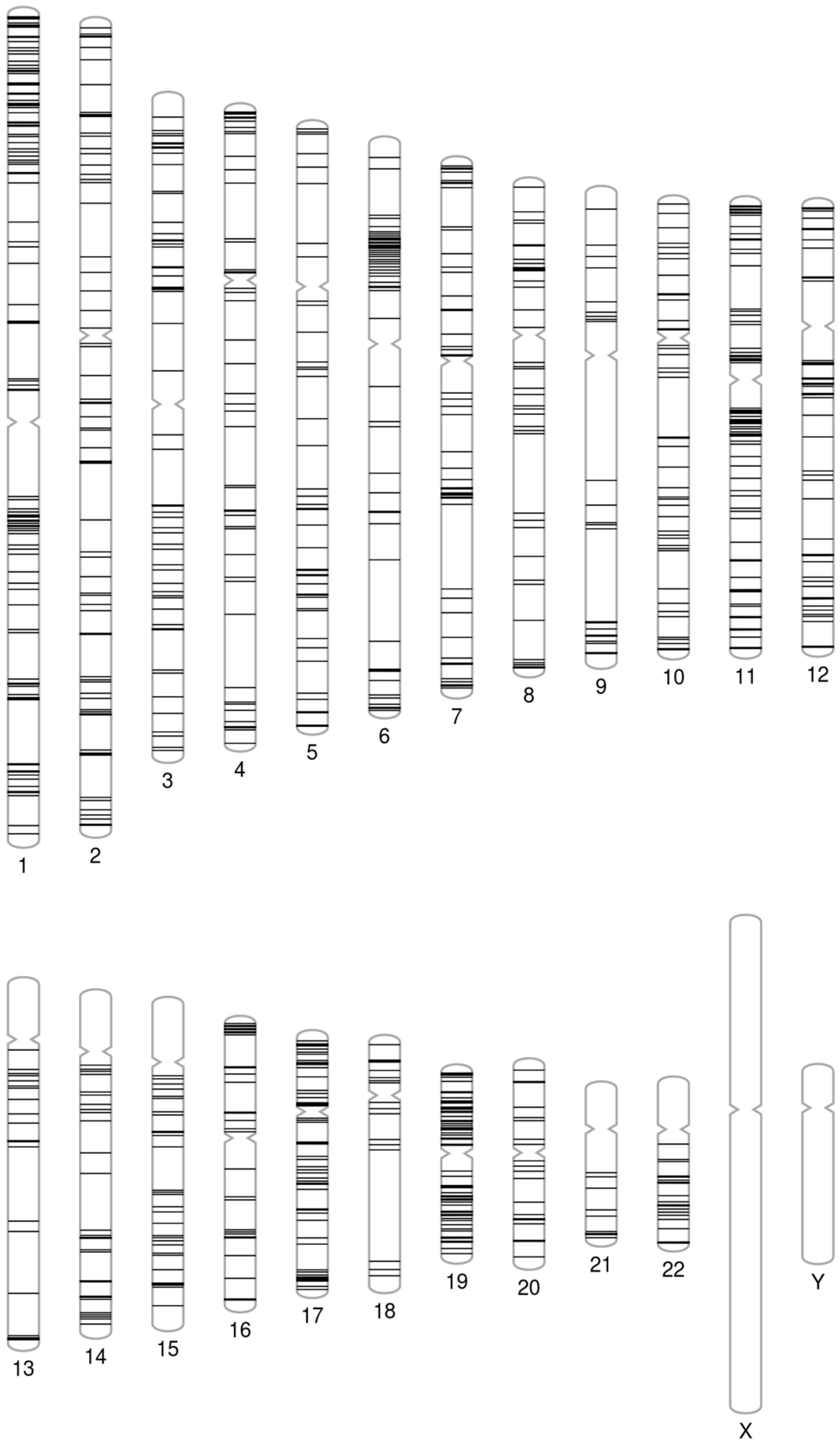

Cluster_15

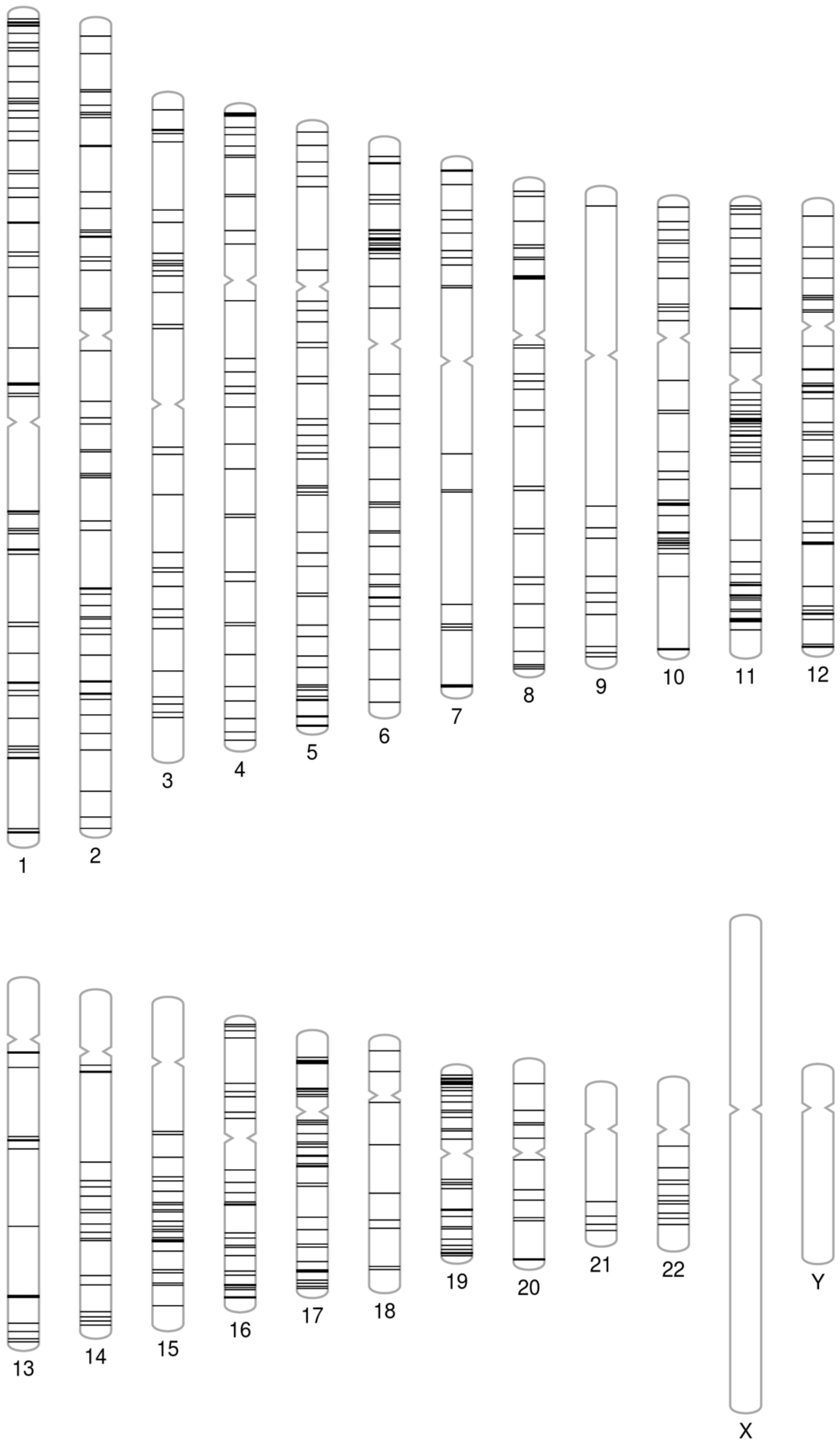

Cluster_16

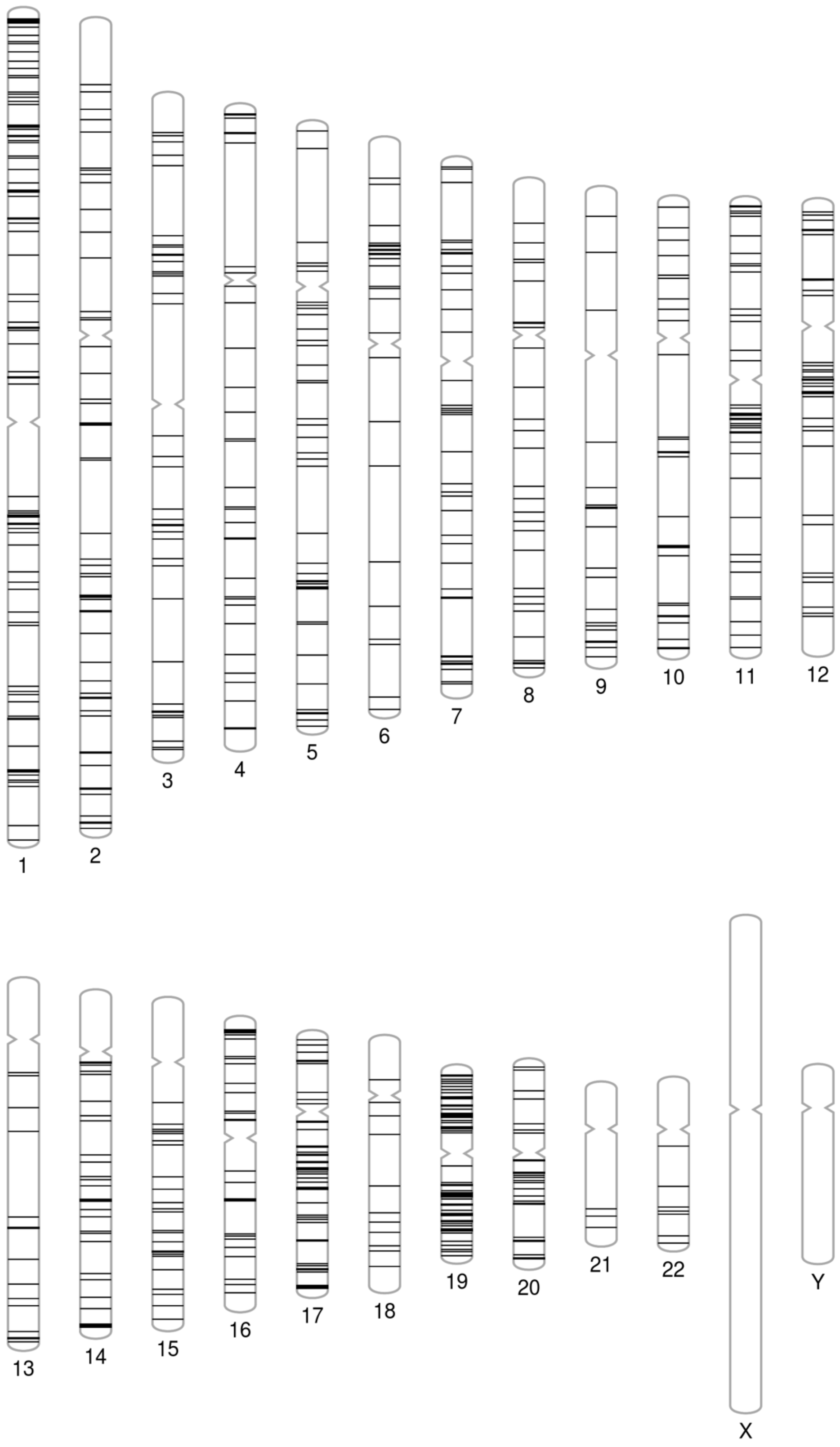

Cluster_17

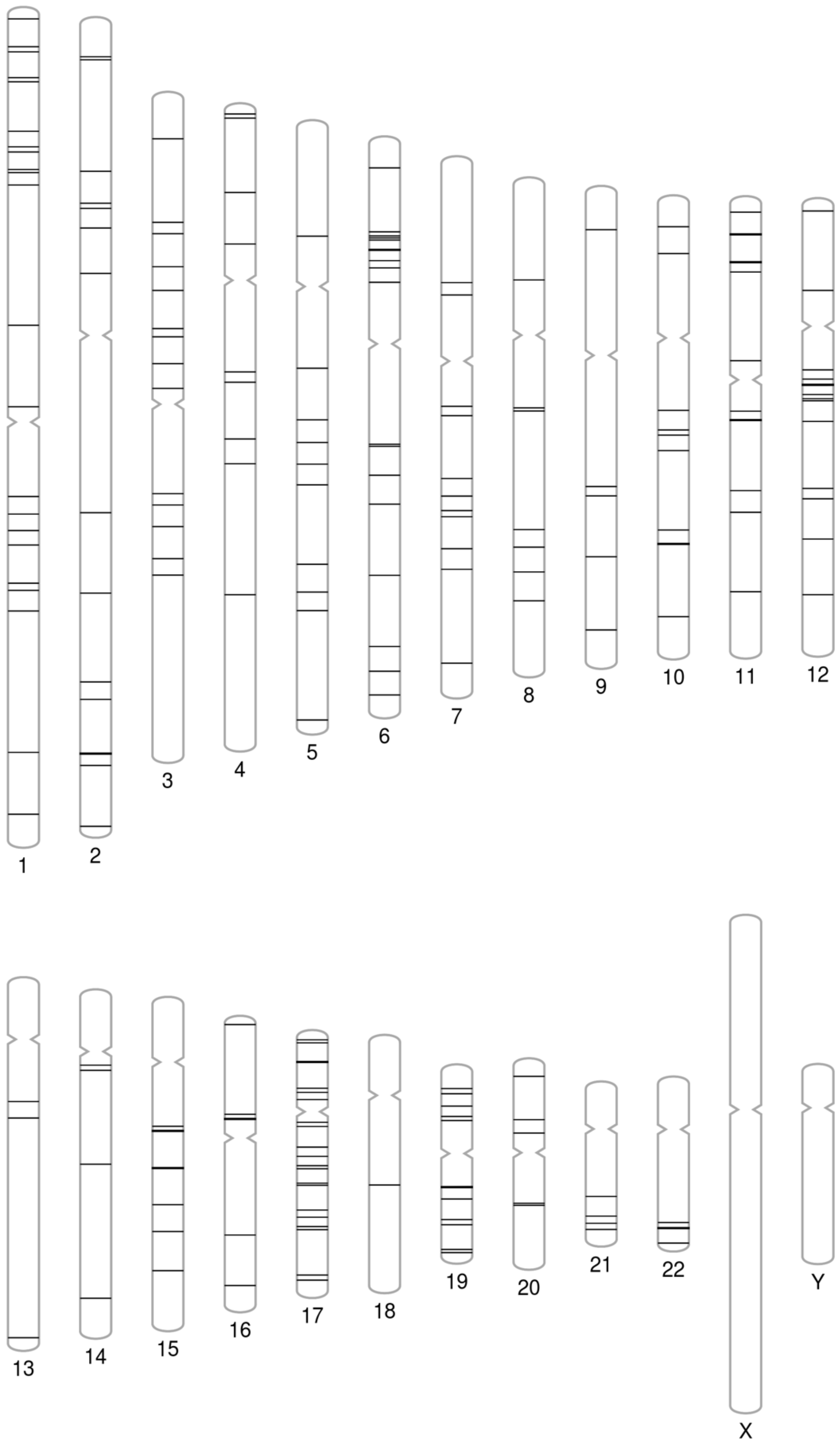

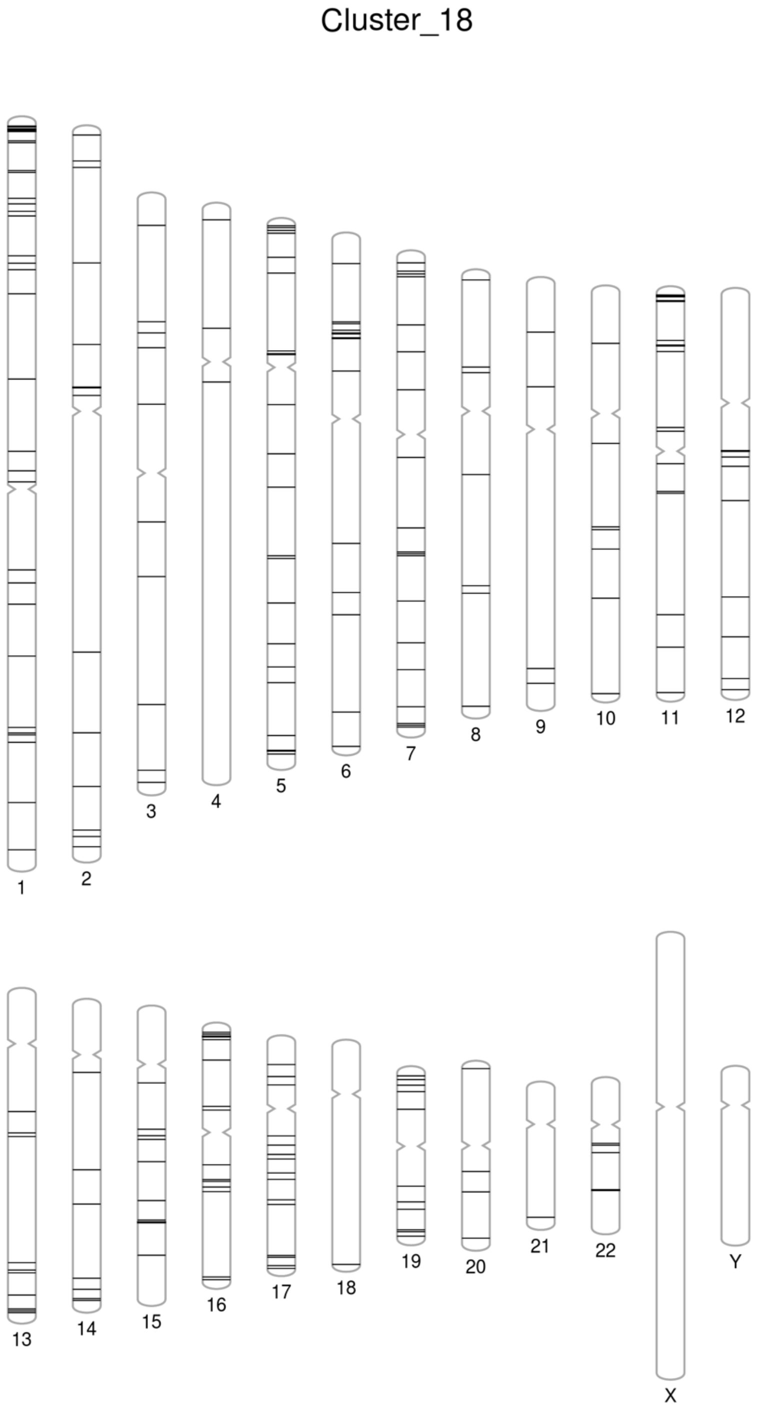

Figure S2. Genomic distribution plot of DNAm clusters.

*The black line denotes the genomic location of a CpG site on a specific chromosome. To facilitate visualization, for clusters with more than 10,000 CpG sites (*i.e.*, cluster-1 to cluster-9), we randomly selected 10,000 sites; for clusters with fewer than 10,000 CpG sites (*i.e.*, cluster-10 to cluster-18), all CpG sites are shown

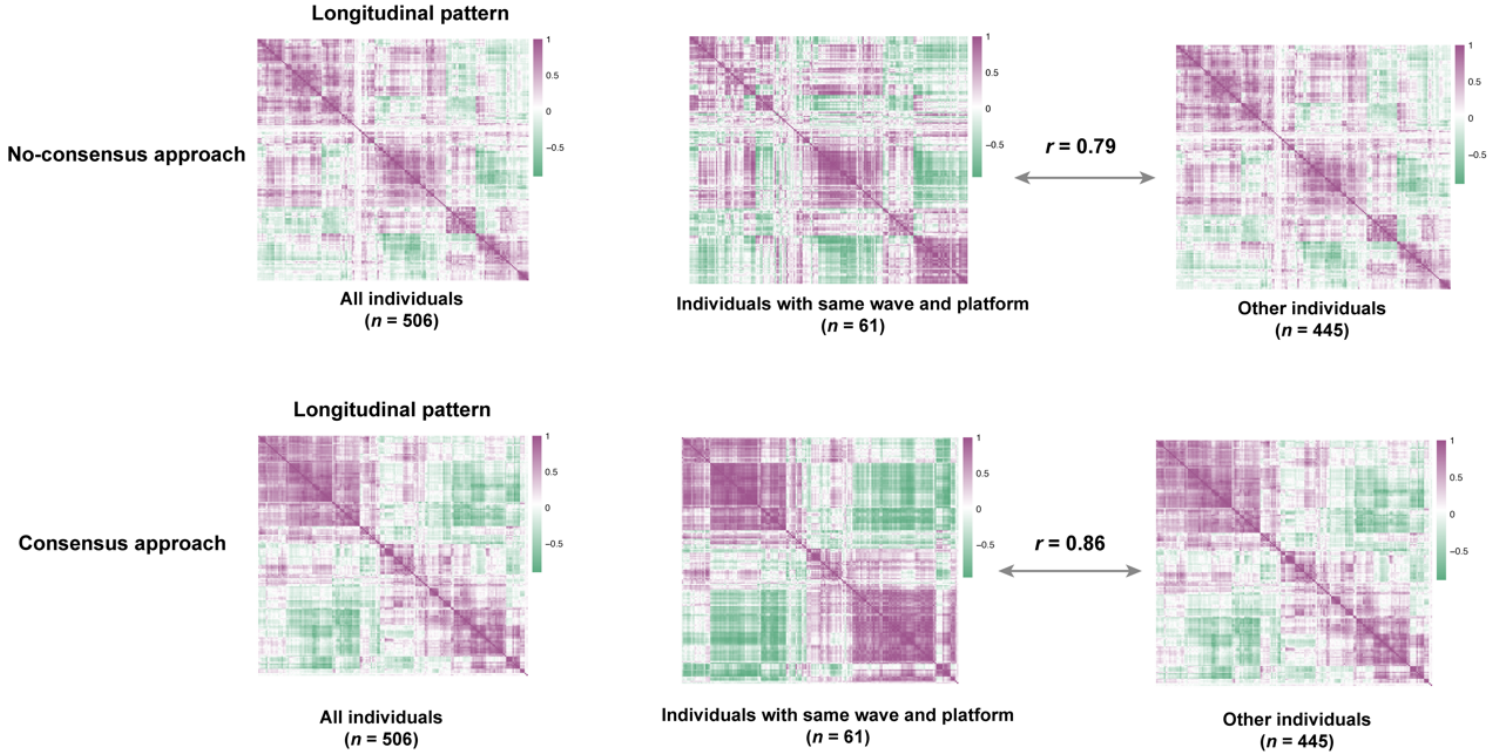

Figure S3. Robustness of the consensus approach.

Comparisons of correlation matrices obtained from the full IMAGEN cohort (450K-to-850K data) with those obtained from the subset of 61 adolescents for whom DNAm at both ages was measured on the same platform and wave (850K-to-850K). The consensus approach demonstrated significantly greater internal consistency ($r_{consensus} = 0.86$) than the non-consensus approach ($r_{non\text{-}consensus} = 0.79$), with the difference statistically supported ($Z_{\mathrm{diff}} = 2.24$, $p_{two\text{-}tail} = 0.025$)

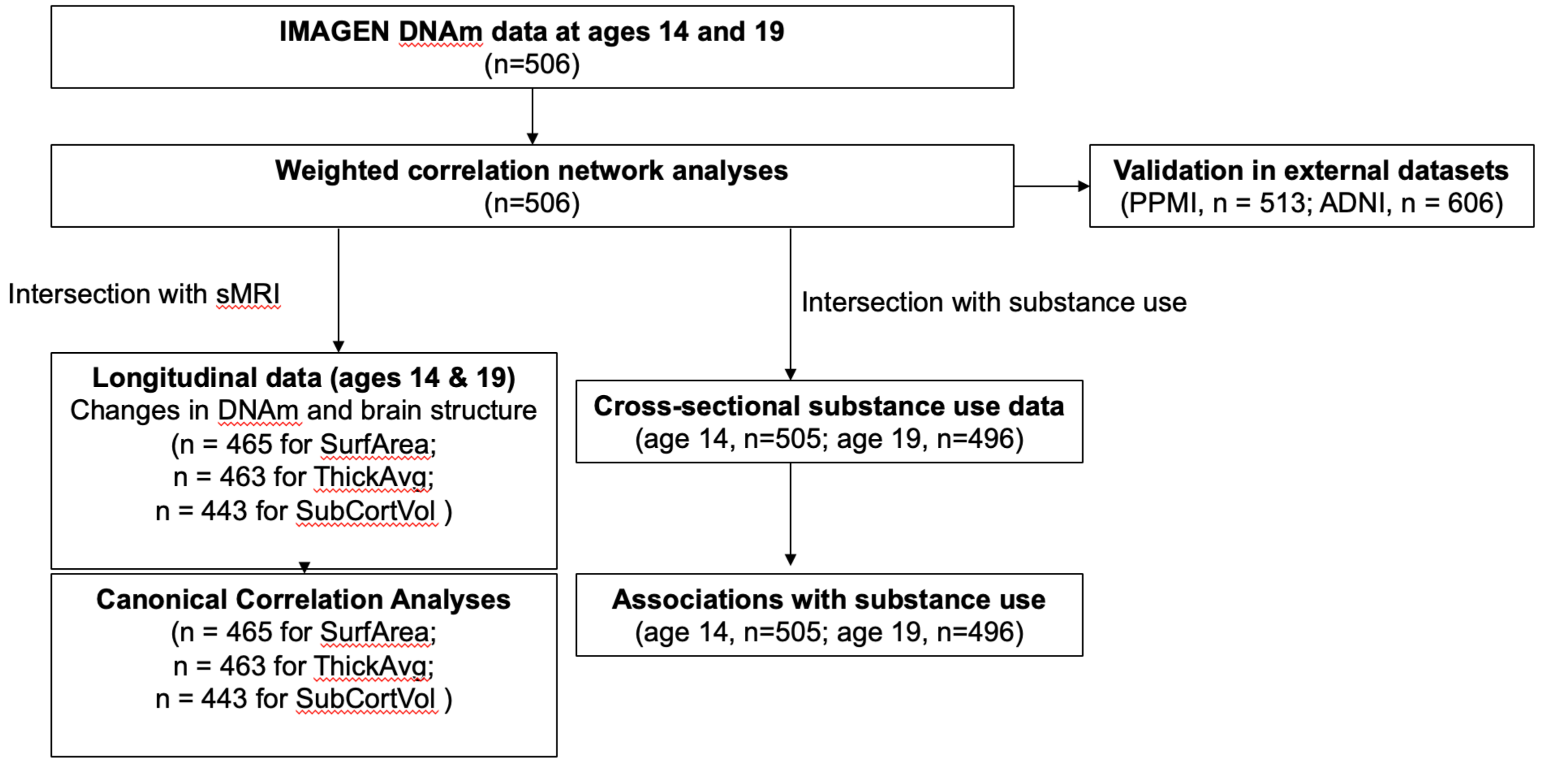

Figure S4. Flowchart of participants included.

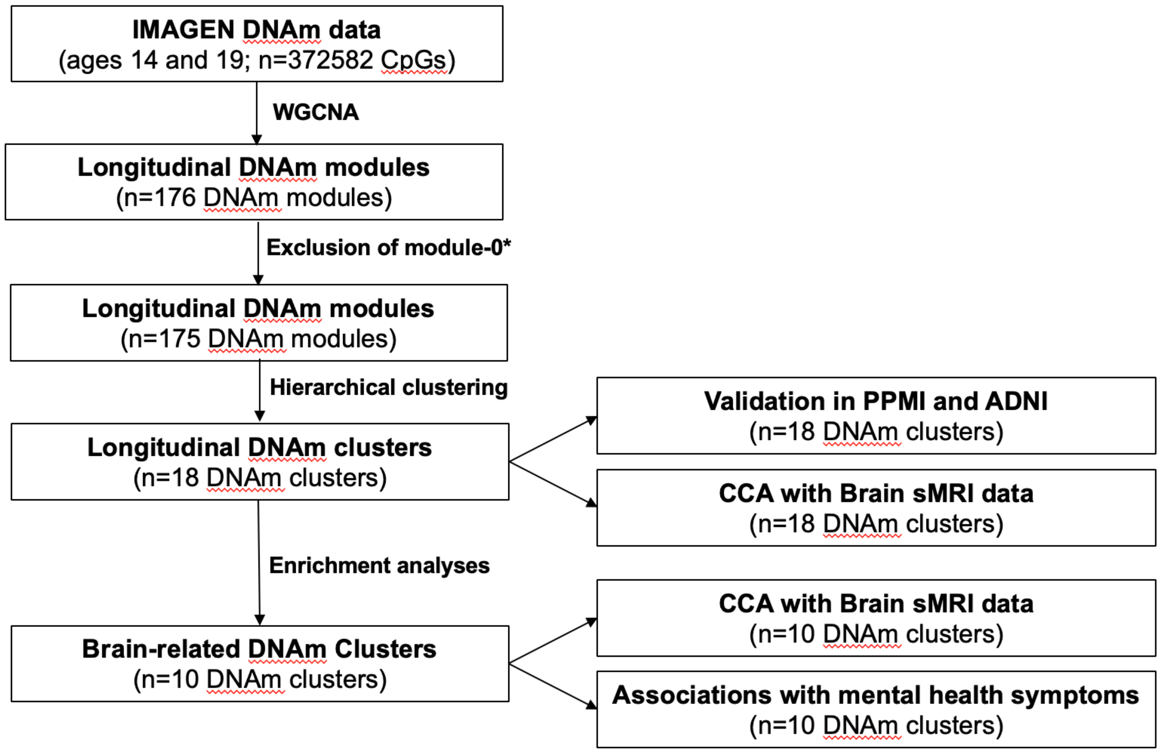

Figure S5. Flowchart highlighting the dimensionality reduction.

*Module-0 was excluded from downstream analyses because it comprised CpGs that did do not correlate with any of the other modules.

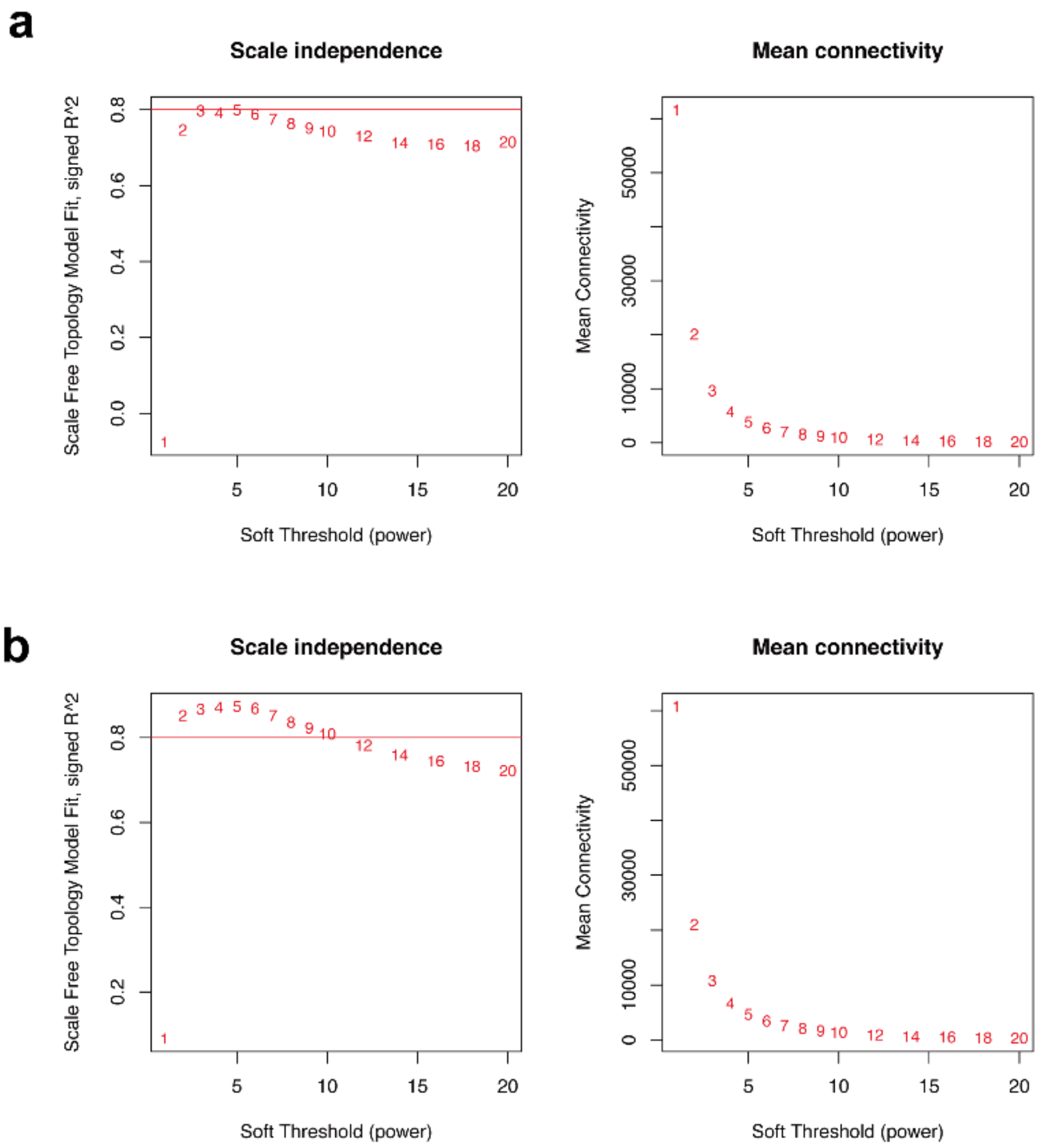

Figure S6. Determination of the soft-threshold parameters in the WGCNA.

Plots represent soft-thresholds for adjacency matrices using DNAm at age 14 **(a)** and age 19 **(b)**. A soft-threshold of 5 represented maximum power in both cases.

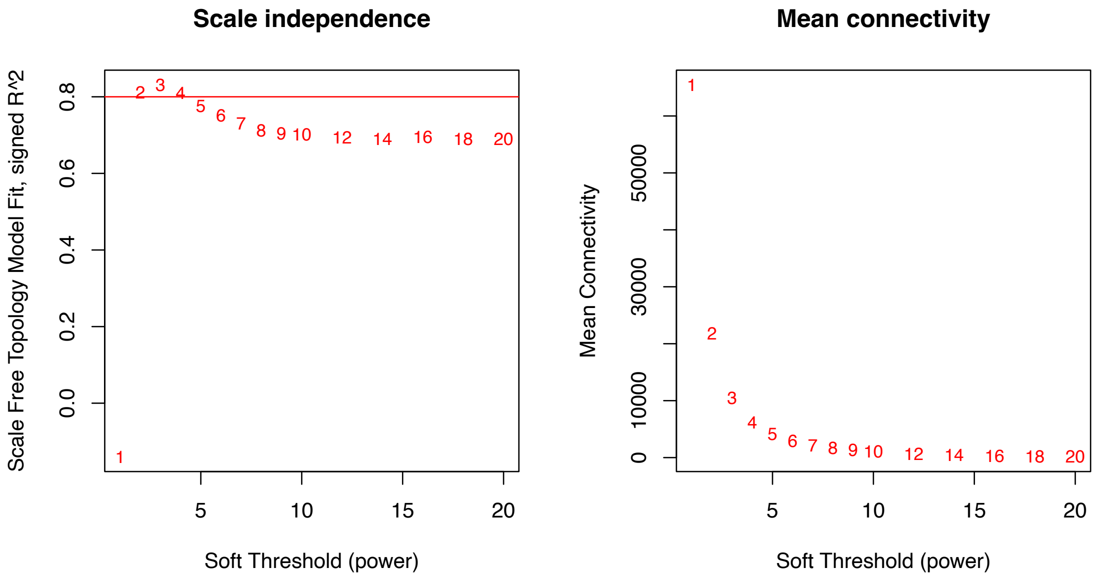

Figure S7. Soft-threshold of DNAm changes for no-consensus approach.

*Hence, we set the soft threshold to three for the no-consensus approach.
